# Inter-organ metabolic feedback via BCAA catabolism regulates glucagon-like hormone secretion in *Drosophila*

**DOI:** 10.1038/s41467-026-72677-1

**Published:** 2026-05-09

**Authors:** Takashi Nishimura, Chisei Arakawa, Yuto Yoshinari

**Affiliations:** 1 Laboratory of Metabolic Regulation and Genetics, Institute for Molecular and Cellular Regulation, Gunma University, Maebashi, Gunma, Japan; 2 Laboratory of Metabolic Regulation and Genetics, Gunma University Graduate School of Medicine, Maebashi, Gunma, Japan; 3 School of Medicine, Faculty of Medicine, Gunma University, Maebashi, Gunma, Japan

**Keywords:** Homeostasis, Insect hormones, Nutrient signalling

## Abstract

Metabolic homeostasis regulated by nutrient-responsive endocrine hormones is essential for organismal survival. In insects, lipid and carbohydrate mobilization is controlled by adipokinetic hormone (Akh), a glucagon-like peptide secreted from neuroendocrine cells. However, whether Akh secretion is subject to negative feedback via its downstream catabolic effects remains unclear. Here, we develop a quantitative assay for Akh using tandem mass spectrometry and show that inter-organ metabolic communication regulates Akh secretion during starvation in *Drosophila*. Metabolic profiling reveals that Akh signaling in the fat body promotes branched-chain amino acid (BCAA) catabolism by inducing BCAA transaminase (Bcat). Loss of Akh signaling impairs clearance of BCAAs derived from fat body autophagy, resulting in Akh hypersecretion. BCAA catabolism is coupled to glutathione biosynthesis and redox homeostasis during nutrient stress. Our findings reveal a feedback mechanism in which Akh signaling regulates its own secretion via amino acid catabolism, linking energy mobilization to redox homeostasis during starvation.

## Abstract (web summary)

Metabolic homeostasis is regulated by nutrient-responsive endocrine hormones. Here, the authors show that in *Drosophila*, a hormone controlling energy use also regulates amino acid breakdown, which in turn feeds back to regulate the hormone’s own secretion during starvation, linking energy mobilization to redox balance.

## Introduction

Metabolic homeostasis is essential for the survival and function of living organisms. Animals maintain energy balance by storing and mobilizing nutrients in response to physiological demands, largely through the action of nutrient-responsive endocrine hormones^1–3^. Among these, insulin and glucagon are key mammalian hormones secreted by pancreatic islets, which regulate anabolism and catabolism, respectively, in target tissues. Insulin is one of the most intensively studied anabolic hormones due to its pivotal role in glucose metabolism and its association with diabetes and obesity^4,5^. Conversely, impaired glucagon secretion or signaling is linked to a variety of metabolic disorders, including hypoglycemia, type 2 diabetes, obesity, metabolic dysfunction-associated steatotic liver disease (MASLD; formerly nonalcoholic fatty liver disease, NAFLD), and hyperaminoacidemia^6^. Insulin/insulin-like growth factor (IGF) signaling is highly conserved between mammals and insects, whereas the glucagon orthologue is not evolutionarily conserved.

In insects, lipid and carbohydrate mobilization is regulated by adipokinetic hormone (Akh), which is considered a functional orthologue of mammalian glucagon due to its analogous catabolic functions^7,8^. In addition, Akh signaling has been implicated in diverse physiological processes, including feeding behavior, locomotion, oxidative stress resistance, and lifespan regulation^8–12^. Akh is produced specifically by the neuroendocrine corpora cardiaca (CC) cells^13,14^. Upon secretion, Akh binds to its cognate G-protein coupled receptor, Akh receptor (AkhR), which is enriched in the fat body, functionally equivalent to mammalian liver and adipocytes^15^. Akh activates the breakdown of triglycerides (TAGs) and glycogen in the fat body through calcium signaling and cAMP/protein kinase A (PKA) pathway^2,7^. In mammals, glucagon signaling in the liver orchestrates a wide array of catabolic processes, including the metabolism of glucose, lipids, and amino acids^6,16,17^. Recent studies have demonstrated that glucagon promotes hepatic amino acid uptake and catabolism via ureagenesis and that amino acids, in turn, stimulate glucagon secretion from pancreatic α-cells^18–20^. Amino acids serve dual roles as both structural components and metabolic fuels, and become increasingly catabolized during starvation to support gluconeogenesis and TCA cycle activity. Maintaining TCA cycle flux through amino acid-derived intermediates is particularly important for sustaining energy production from lipid oxidation. Despite these insights, the role of Akh in amino acid catabolism remains largely unexplored in insects.

The regulatory mechanisms of Akh secretion have been actively studied in the genetically tractable model organism *Drosophila melanogaster*. Akh release is regulated by circulating sugar levels through ATP-sensitive potassium channels^13,21,22^. In addition, multiple humoral signals originating from the midgut and skeletal muscle modulate Akh secretion partly in a sex-dependent manner^8,23^. Moreover, specific neurons in the central brain project directly to the CC cells to regulate Akh secretion^24–26^. While the upstream regulation of Akh release in response to dietary cues has been progressively elucidated, direct measurements of Akh secretion have been limited. Additionally, in mammals, endocrine hormone levels are generally modulated via feedback loops mediated by their downstream effects^27^. Disruption of such feedback mechanisms often underlies endocrine pathologies due to persistent hormone action. However, whether Akh secretion is subject to negative feedback through its own downstream signaling remains unknown.

In this study, we established a robust quantitative method for measuring Akh using liquid chromatography with tandem mass spectrometry (LC-MS/MS) and examined how Akh levels respond to changes in nutrient state. We found that Akh secretion is negatively regulated by Akh signaling in the fat body during starvation. Through comprehensive, time-course analyses of metabolic profiles, we revealed that catabolism of branched-chain amino acids (BCAAs), regulated by Akh signaling, is a key factor that fine-tunes Akh secretion. Furthermore, BCAA catabolism is functionally coupled to glutathione (GSH) synthesis and redox homeostasis, which helps counteract oxidative stress during starvation. These findings uncover a previously unrecognized inter-organ feedback circuit and broaden our understanding of the Akh-BCAA axis in coordinating energy and redox homeostasis in response to nutrient deprivation.

## Results

### Quantitative measurement of *Drosophila* Akh by LC-MS/MS

**Fig. 1:**
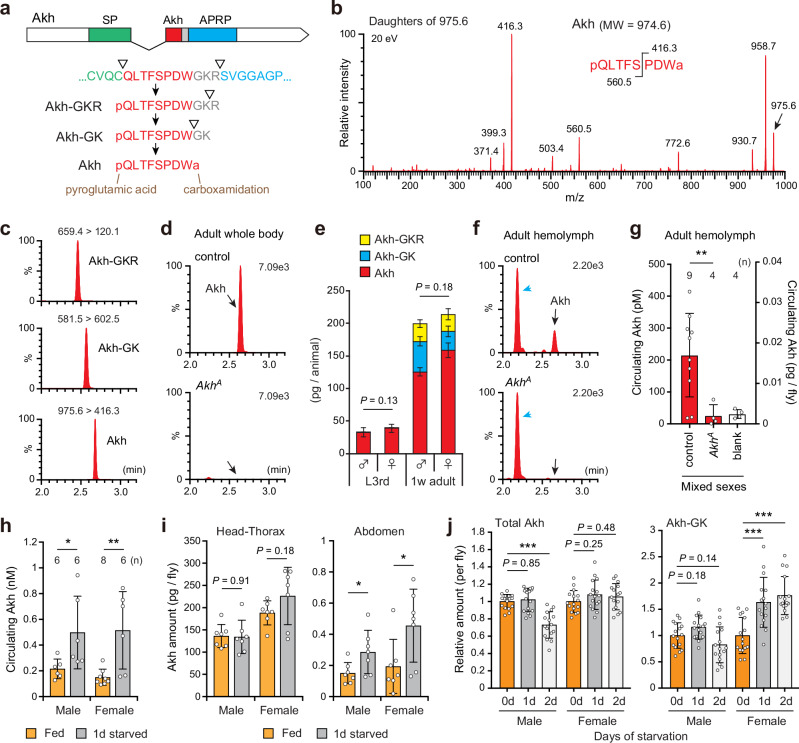
Quantitative measurement of *Drosophila* Akh and its starvation response. **a** Genomic organization of the *Akh* locus and processing of the Akh prepropeptide. The *Akh* gene encodes a prohormone consisting of a signal peptide (green), the Akh octapeptide (red), a linker containing protease cleavage sites (gray), and the APRP (blue). Akh is processed sequentially by a signal peptidase, a prohormone convertase, and carboxypeptidases. Inverted triangles indicate cleavage sites. “pQ” and “Wa” denote pyroglutamic acid and tryptophan carboxamide, respectively. **b** MS2 spectrum of product ions from synthetic Akh generated by low-energy collision-induced dissociation (20 eV). The inset shows the major fragment ions. The arrow indicates residual precursor ions. **c** Representative chromatograms of synthetic Akh peptides analyzed by multiple reaction monitoring (MRM) with the indicated transitions. **d** Representative MRM chromatograms detecting Akh peptides in control flies but not in *Akh* mutants. **e** Quantification of Akh, Akh-GK, and Akh-GKR per animal. L3rd, late third instar larva; 1w adult, one-week-old adult. Statistical analyses were performed using the sum of Akh and Akh precursors. **f** Representative MRM chromatograms detecting circulating Akh in hemolymph of control but not in *Akh* mutants. Blue arrowheads indicate non-specific signals also present in mutants. **g** Circulating Akh concentration in control and *Akh* mutants. Mixed-sex flies were analyzed. **h** Circulating Akh concentration in hemolymph of adult flies maintained on a standard diet (0 d) or starved for 1 day (1 d starved). **i** Total Akh amounts in the head/thorax and abdomen under fed and starved conditions. **j** Relative amounts of total Akh and Akh-GK (per fly) during starvation. 1 d, 1 day starved; 2 d, 2 days starved. Results are presented as mean ± SD (**e**,**g**–**j**); *n* = 6 (**e**), 7 (**i**), or 16 (**j**) batches. Values of n indicate the number of batches (**g**,**h)**. Statistical tests: unpaired two-tailed Student’s *t*-test (**e**,**h**,**i**), unpaired two-tailed Welch’s *t*-test (**g**), one-way ANOVA with Dunnett’s post hoc test (**j**); *p < 0.05; **p < 0.01; ***p < 0.001. Source data are provided as a Source Data file, which also includes exact *P* values.

The *Akh* gene is locally transcribed in CC cells as a prohormone that gives rise to the mature Akh and adipokinetic hormone precursor-related peptide (APRP) after proteolytic processing (Fig. 1a)^28^. The mature Akh is an octapeptide with modifications at both N- and C-terminal ends: pGlu-Leu-Thr-Phe-Ser-Pro-Asp-Trp-NH_2_ (where pGlu is pyroglutamic acid and Trp-NH_2_ is tryptophan carboxamide)^29,30^. To quantify Akh in vivo, we first established an LC-MS/MS–based assay using synthetic mature Akh peptide as a standard (Fig. 1b, Supplementary Fig. 1a-c). Similarly, we optimized detection methods for two Akh precursors, Akh-GK and Akh-GKR (Fig. 1c, Supplementary Fig. 2a, b), which have previously been identified both in vitro and in vivo^28,30–32^.

We examined the total amount of Akh peptides in whole-fly extracts and detected specific signals in controls but not in *Akh*-null mutants (Fig. 1d). The amounts of Akh and Akh-GK differed significantly between males and females in one-week-old adults (Supplementary Fig. 2c). The mature Akh content per fly was approximately 100–120 pg in males and 140–160 pg in females. These values are consistent with earlier estimates obtained by HPLC^29,33^. In contrast, Akh-GK levels were higher in males (~50 pg per fly) than in females (~30 pg per fly). Akh-GKR was less abundant than Akh and Akh-GK, and no significant sex differences were observed (~20 pg per fly in both sexes). Taken together, these results indicate that total Akh peptide levels, including mature and precursor forms, are roughly equivalent between sexes (Fig. 1e). Notably, we detected substantially lower levels of Akh (~40 pg per animal) in late third instar larvae, with Akh-GK and Akh-GKR being nearly undetectable (Fig. 1e, Supplementary Fig. 2d). These results suggest stage-specific regulation of Akh peptide levels.

We next asked whether our LC-MS/MS method could reliably detect circulating Akh in the adult hemolymph under normal feeding conditions. Although the signal was close to our detection limit, a specific Akh signal was detected in control but not in *Akh* mutant hemolymph, with an average concentration of 200 pM in mixed-sex samples (Fig. 1f, g). This value is close to the reported range of half maximal effective concentrations (EC_50_) for AkhR activation, which spans from 230 pM to 800 pM^34–36^, supporting the physiological relevance of our assay. In contrast, we were unable to detect consistent Akh precursor signals in hemolymph. Importantly, the amount of circulating Akh was estimated to be ~0.02 pg per adult, based on a hemolymph volume of 80 nL per fly as reported previously^37,38^. This corresponds to just 0.02% of total Akh in the whole body, suggesting that the vast majority of Akh resides in its intracellular, storage form. Together, these data validate our LC-MS/MS assay as a reliable method for quantifying both stored and circulating Akh peptides. Because the Akh-GKR signal exhibited high background noise in vivo and strong adhesion to plastic surfaces, we focused subsequent analyses on mature Akh and Akh-GK.

### Starvation response in Akh peptide and mRNA levels

Since Akh is a major catabolic hormone^2,7^, we next examined how Akh secretion responds to nutrient deprivation in adult flies. As expected, Akh levels increased in the hemolymph one day after starvation in both males and females (Fig. 1h). Akh is secreted from CC cells located anterior to the gut proventriculus, while AkhR is enriched in the fat body. To further assess Akh distribution, we separately measured Akh levels in the head/thorax (containing the CC) and abdomen (including the abdominal fat body). Under fed conditions, Akh was predominantly detected in the head/thorax, with minimal levels (<0.2%) in the abdomen (Fig. 1i). Upon starvation, Akh levels in the abdomen increased, whereas levels in the head/thorax remained unchanged. Although this method does not distinguish between intra- and extracellular localization, it provides a practical proxy for monitoring Akh secretion without hemolymph collection.

A prior study using ELISA reported that one day of starvation reduced total Akh levels in the head-thorax by more than half in both sexes, likely reflecting secretion and subsequent degradation in the hemolymph or target tissues^39^. In contrast, our LC-MS/MS data showed that total whole-body Akh levels remained unchanged after one day of starvation in both sexes (Fig. 1j). Akh levels moderately declined after two days of starvation in males, but not in females. However, because over half of the male flies were dead at that point, the decline possibly reflects a pre-mortem physiological response. Depending on certain genotypes and conditions, one day of starvation significantly reduced total Akh levels by 20–30% in control males (see below). Interestingly, Akh-GK levels markedly increased in starved females but not in males.

To examine transcriptional responses, we assessed *Akh* and *AkhR* mRNA expression. *Akh* expression levels did not significantly change during starvation, while *AkhR* expression decreased in males (Supplementary Fig. 3a). In contrast, females exhibited starvation-induced upregulation of both *Akh* and *AkhR*, suggesting a sex-specific response. The increase in *Akh* transcript levels in females likely contributes to increased Akh translation, resulting in elevated Akh-GK precursor levels.

### *AkhR* mutants promote Akh secretion and deplete Akh during starvation

**Fig. 2:**
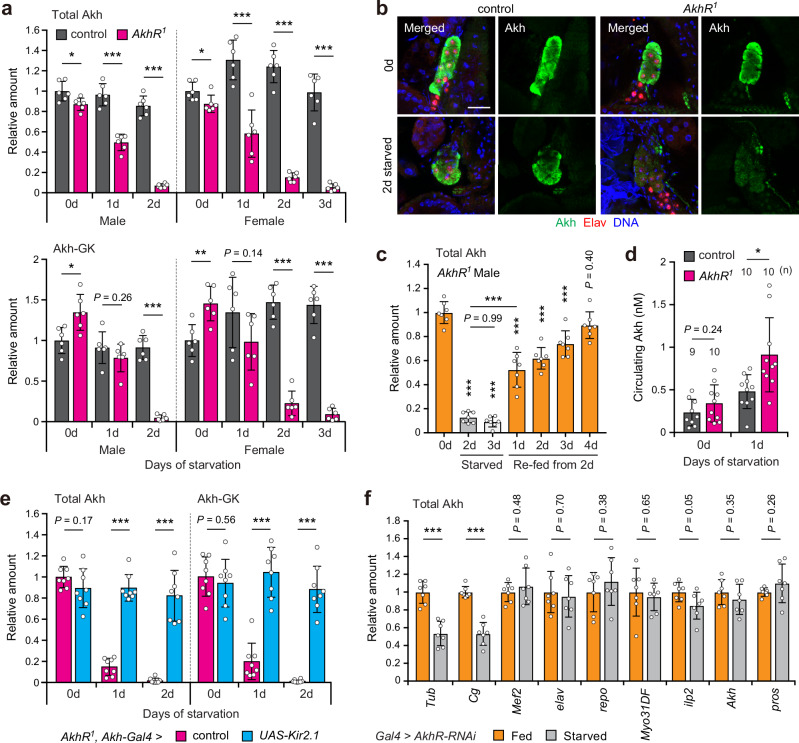
*AkhR* mutants promote Akh secretion and deplete Akh during starvation. **a** Relative amounts of total Akh and Akh-GK per fly during starvation. Adult flies maintained on a standard diet (0 d) were transferred to an agar-only diet and collected at the indicated days (0–3 d). **b** Immunofluorescence of Akh-producing CC cells dissected from female flies of the indicated genotypes and dietary conditions. Samples were stained for Akh (green), Elav (magenta) and DNA (blue). Representative z-stack images are shown. Scale bar: 20 μm. **c** Total Akh levels in *AkhR* mutants after re-feeding. Flies starved for 2 days were returned to a standard diet and collected at the indicated days. **d** Circulating Akh concentrations in hemolymph of control and *AkhR* mutant flies under fed (0 d) and 1-day starved conditions. **e** Total Akh and Akh-GK levels in *AkhR* mutants during starvation with CC-specific Kir2.1 expression to inhibit secretion. **f** Relative amounts of total Akh in *AkhR* knockdown flies using the indicated Gal4 drivers under fed and starved conditions. Results are presented as mean ± SD; n = 6 (**a**), 7 (**c,** **f**), or 8 (**e**) batches. Values of n indicate the number of batches (**d)**. Statistical tests: unpaired two-tailed Student’s *t*-test (**a**,**d**,**e**,**f**), one-way ANOVA with Tukey’s post hoc test (**c**); *p < 0.05; **p < 0.01; ***p < 0.001. Source data are provided as a Source Data file, which also includes exact *P* values.

To further investigate the regulatory mechanisms of Akh secretion, we analyzed Akh levels in *AkhR* mutants. Unexpectedly, total Akh levels were dramatically reduced in both sexes during starvation (Fig. 2a). Akh-GK levels also declined, indicating depletion of both mature and precursor forms of Akh. These findings were confirmed using two independent *AkhR* mutants generated by CRISPR/Cas9 (Supplementary Fig. 3b). Immunofluorescence analysis further confirmed a marked reduction of Akh signals in *AkhR* mutant CC cells upon starvation compared to control flies (Fig. 2b). Notably, under fed conditions, basal Akh levels were slightly lower, whereas Akh-GK levels were higher in *AkhR* mutants than in controls (Fig. 2a, Supplementary Fig. 3b), suggesting that Akh production and secretion dynamics are already altered prior to starvation.

Since *AkhR* mutants are known to exhibit increased starvation resistance^14,15,28,40^, the observed Akh depletion is unlikely to reflect late-stage systemic failure or nonspecific organismal deterioration. *Akh* mRNA remained detectable in *AkhR* mutants at levels comparable to controls under starvation (Supplementary Fig. 3a), indicating that CC cells remain functional. Consistently, Akh levels gradually recovered after refeeding on a standard diet (SD) (Fig. 2c, Supplementary Fig. 3c), suggesting that Akh depletion is reversible and responsive to nutrient state.

If secreted Akh is rapidly degraded in the hemolymph and has a short half-life, then sustained secretion from CC cells could lead to systemic depletion. Given that endocrine hormones are often negatively regulated by their own downstream signals^6,17^, we hypothesized that *AkhR* mutants fail to suppress Akh secretion due to impaired feedback regulation. Supporting this notion, *AkhR* mutants exhibited elevated circulating Akh levels during starvation (Fig. 2d). Furthermore, blocking secretion by expressing the inward-rectifier potassium channel *Kir2.1* in CC cells completely prevented the starvation-induced depletion of both Akh and Akh-GK (Fig. 2e, Supplementary Fig. 3d). Taken together, these findings suggest that Akh depletion in *AkhR* mutants during starvation is due to hypersecretion and subsequent degradation in the hemolymph caused by a lack of negative feedback.

Based on single-cell RNA-seq resources^41^, *AkhR* is enriched in fat body cell populations, while bulk tissue datasets such as FlyAtlas2^42^ report *AkhR* expression across multiple tissues. To identify the tissue responsible for feedback regulation of Akh secretion, we performed tissue-specific knockdown of *AkhR*. Ubiquitous knockdown of *AkhR* by *Tub-Gal4* reduced total Akh levels by half during starvation (Fig. 2f). Fat body-specific knockdown using *Cg-Gal4* recapitulated this reduction, whereas knockdown using other Gal4 drivers showed no obvious effects. Adult-onset knockdown of *AkhR* after eclosion using two independent fat body drivers, *Lpp-Gal4* and *r4-Gal4*, also led to a reduction in total Akh levels during starvation (Supplementary Fig. 3e). These results indicate that AkhR in the fat body is critical for suppressing excess Akh secretion from CC cells during starvation.

### Nutrient and metabolic regulation of Akh secretion in *AkhR* mutants

**Fig. 3:**
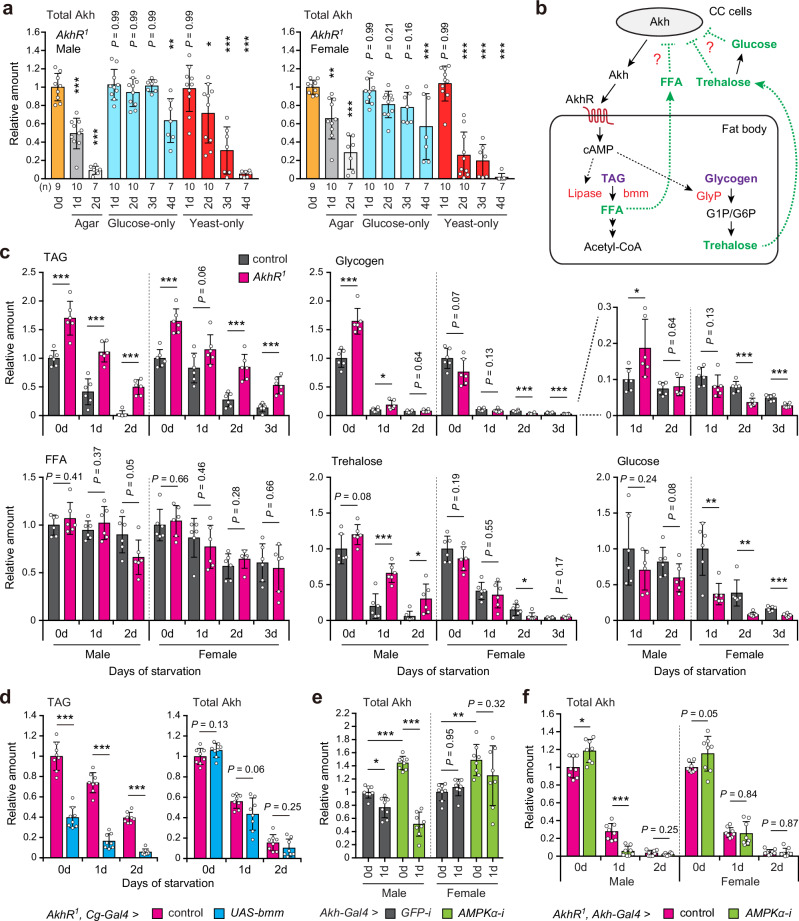
Nutrient and metabolic regulation of Akh secretion in *AkhR* mutants. **a** Relative amounts of total Akh in *AkhR* mutants under different dietary conditions. Flies maintained on a standard diet (0 d) were transferred to vials containing agar-only, glucose-only, or yeast-only diets and collected at the indicated days (1–4 d). **b** Simplified schematic illustrating Akh function in lipid and glycogen mobilization in the fat body. Metabolites derived from stored TAG and glycogen are hypothesized to negatively feedback and suppress Akh secretion from CC cells. FFA, free fatty acids; G1P/G6P, glucose-1- or -6-phosphate. **c** Relative changes in total TAG levels (sum of signal intensities for C38–C52 TAG species), total FFA levels (sum of signal intensities for C12–C20 FFA species), glycogen, trehalose, and glucose in control and *AkhR* mutants during starvation. An enlarged view of glycogen contents is shown on the right. **d** Relative amounts of TAG and total Akh in *AkhR* mutants during starvation with fat body-specific *bmm* overexpression. **e** Relative amounts of total Akh in CC-specific *AMPKα* knockdown flies. *GFP-i*, *GFP-RNAi*; *AMPKα-i*, *AMPKα-RNAi*. **f** Relative amounts of total Akh in *AkhR* mutants during starvation with CC-specific *AMPKα* knockdown. Results are presented as mean ± SD; n = 6 (**c**) or 8 (**d**–**f**) batches. Values of n indicate the number of batches (**a)**. Statistical tests: one-way ANOVA with Dunnett’s post hoc test (**a**), unpaired two-tailed Student’s *t*-test (**c**,**d**,**f**), one-way ANOVA with Tukey’s post hoc test (**e**); *p < 0.05; **p < 0.01; ***p < 0.001. Source data are provided as a Source Data file, which also includes exact *P* values.

To determine which nutrients are essential for suppressing Akh hypersecretion in *AkhR* mutants, we conducted dietary intervention assays using different food compositions. A glucose-only diet effectively suppressed Akh depletion for up to three days, although prolonged feeding eventually reduced total Akh levels (Fig. 3a). In contrast, a yeast-only diet suppressed Akh depletion after one day but caused an earlier decline in Akh levels than the glucose-only diet. Interestingly, females depleted Akh more rapidly than males under yeast-only diet conditions. These results suggest that dietary carbohydrates are particularly important for suppressing Akh hypersecretion in *AkhR* mutants, although multiple nutrients are likely involved in Akh secretion, potentially in a sex-dependent manner.

Akh plays a role in lipid mobilization during starvation, as well as in regulating circulating trehalose levels to maintain systemic energy metabolism^8,12^. Decreases in circulating trehalose or glucose concentrations induce CC cell depolarization and stimulate Akh secretion in *Drosophila* larvae^13^. Consistently, the energy-sensing kinase AMPK has been proposed to promote Akh secretion by modulating membrane potential under low energy conditions^43^. Thus, we hypothesized that the observed Akh hypersecretion in *AkhR* mutants is caused by insufficient energy catabolism resulting in reduced metabolite levels, which would otherwise prevent excessive Akh secretion (Fig. 3b).

To clarify the contribution of lipid and carbohydrate metabolism, we conducted metabolic analysis in whole flies. As reported previously^15^, *AkhR* mutants showed increased basal TAG levels, which decreased gradually during starvation. However, free fatty acid (FFA) levels remained unchanged compared to control flies during starvation in both sexes (Fig. 3c). We further induced fat body lipolysis by overexpressing the triglyceride lipase *brummer* (*bmm*), which acts in parallel with Akh-induced lipolysis (Fig. 3b). Although this manipulation reduced TAG levels in the *AkhR* mutant background, it failed to prevent Akh depletion during starvation (Fig. 3d, Supplementary Fig. 4a). These findings suggest that lipid-derived metabolites are unlikely to be the primary negative regulators of Akh secretion.

Interestingly, carbohydrate metabolism showed distinct sex-specific patterns in *AkhR* mutants. In males, basal glycogen and trehalose levels were higher in *AkhR* mutants under fed conditions and remained elevated compared to controls even after starvation (Fig. 3c). In contrast, *AkhR* mutant females depleted glycogen and trehalose more rapidly than control flies during starvation. Consistently, glucose levels in *AkhR* mutant males decreased to a similar extent as in controls, whereas mutant females showed a significantly greater decrease than controls. These results raise the possibility that reductions in trehalose and glucose levels contribute to excessive Akh secretion in *AkhR* mutant females.

Given the potential link between carbohydrate depletion and Akh secretion, we further investigated whether AMPK is involved in Akh hypersecretion. Supporting previous findings^43^, knockdown of *AMPK α subunit* (*AMPKα*) in CC cells significantly increased total Akh and Akh-GK levels in whole flies under fed conditions (Fig. 3e, Supplementary Fig. 4b), suggesting that AMPK positively regulates basal Akh secretion. Unexpectedly, however, *AMPKα* knockdown led to a marked reduction in total Akh levels during starvation, especially in males. Furthermore, *AMPKα* knockdown failed to prevent Akh depletion in *AkhR* mutants during starvation in both sexes (Fig. 3f). These results indicate that Akh secretion is governed by both AMPK-dependent and -independent mechanisms in response to energy fluctuations. Collectively, we concluded that Akh hypersecretion in *AkhR* mutants cannot be attributed solely to reduced trehalose and glucose levels during starvation, particularly since such reductions were not observed in males.

### Akh promotes BCAA catabolism during starvation

Recent studies of the liver-α cell axis have shown that glucagon signaling is more closely associated with amino acid metabolism than with glucose metabolism^6,17^. Specifically, glucagon stimulates hepatic amino acid uptake and catabolism in part by regulating metabolic gene expression^16^. However, the role of Akh in amino acid metabolism remains unexplored. We therefore examined the physiological function of Akh signaling in this context.

**Fig. 4:**
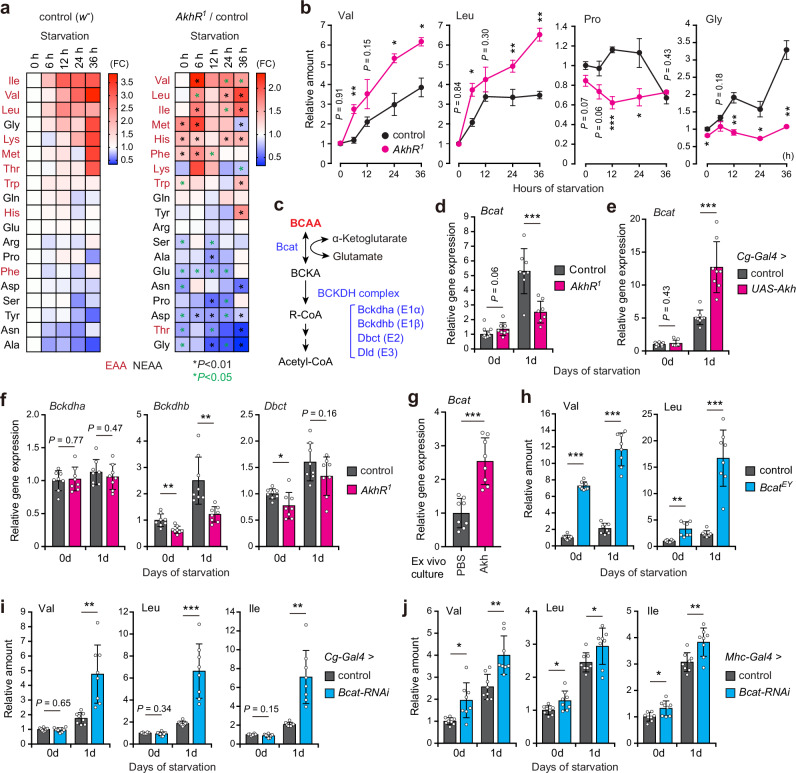
Akh promotes BCAA catabolism during starvation. **a** Heatmaps of amino-acid fold changes during starvation. Control: fold change relative to 0 h in control. *AkhR* mutants: fold change relative to time-matched control. Red and blue indicate increased or decreased metabolites, respectively. FC, fold change; EAA, essential amino acids; NEAA, non-essential amino acids. **b** Relative amounts of indicated amino acids in control and *AkhR* mutants during starvation. Fold changes are shown relative to the average value in control at 0 h (set to 1). **c** Simplified schematic of BCAA catabolism by Bcat and the branched-chain α-keto acid dehydrogenase (BCKDH) complex, which comprises E1α, E1β, E2, and E3. BCKAs, branched-chain keto acids; R-CoA, branched-chain acyl-CoA. **d** Relative *Bcat* mRNA levels in *AkhR* mutants during starvation. 0 d, fed; 1 d, 1 day starved. **e** Relative *Bcat* mRNA levels in *Akh* overexpressing flies during starvation. **f** Relative mRNA levels of BCKDH components in *AkhR* mutants during starvation. **g** Relative *Bcat* expression in abdominal carcasses incubated ex vivo with synthetic Akh peptide for 2 h. **h** Relative amounts of valine and leucine in *Bcat* mutants during starvation. **i** Relative amounts of BCAAs in fat body-specific *Bcat* knockdown flies. **j** Relative amounts of BCAAs in muscle-specific *Bcat* knockdown flies. Results are presented as mean ± SEM (**b**) or mean ± SD (**d**–**j**); *n* = 4 (**a**,**b**) or 8 (**d**–**j**) batches. Statistical tests: unpaired two-tailed Welch’s *t*-test (**a**,**b**,**h**–**j**), unpaired two-tailed Student’s *t*-test (**d**–**g**); *p < 0.05; **p < 0.01; ***p < 0.001 (except in **a**). Source data are provided as a Source Data file, which also includes exact *P* values.

In control male flies, starvation induced widespread changes in whole-body amino acid levels. Many essential amino acids (EAAs), including BCAAs, gradually increased, whereas most non-essential amino acids (NEAAs), except glycine, decreased in a starvation time-dependent manner (Fig. 4a, b). Because these measurements report whole-body amino-acid abundances (i.e., pool sizes), they reflect the net balance between production (e.g., autophagy/protein degradation) and consumption (e.g., protein synthesis and downstream metabolism). Therefore, these data do not directly report metabolic flux. In *AkhR* mutants, amino acid profiles were altered under both fed and starved conditions. Specifically, EAAs, except for threonine, were elevated, while many NEAAs were reduced relative to controls during starvation. Although BCAA levels increased in control flies, the increase was more pronounced in *AkhR* mutants but occurred only after starvation (Fig. 4b, Supplementary Fig. 5a). A time-course analysis further revealed that the timing of Akh reduction (>12 h after starvation) in *AkhR* mutants coincided with increased BCAA levels during starvation (Supplementary Fig. 5b). Female *AkhR* mutants showed a comparable pattern of amino acid changes, with increased EAAs and decreased NEAAs, although sex-specific differences were apparent (Supplementary Fig. 5b-d).

Branched-chain amino acid transaminase (Bcat) catalyzes the first step in BCAA catabolism by converting BCAAs and α-ketoglutarate into branched-chain α-keto acids (BCKAs) and glutamate (Fig. 4c). BCKAs are further processed by the branched-chain α-keto acid dehydrogenase (BCKDH) complex, which comprises Bckdha (E1α), Bckdhb (E1β), Dbct (E2), and Dld (E3), and ultimately yields acetyl-CoA and succinyl-CoA. Whereas BCKDH components are broadly expressed across tissues, *Bcat* shows high expression in the fat body, where *AkhR* is enriched, as well as in muscle and oenocytes (Supplementary Fig. 6a). In addition, the Developmental Proteome database^44^ indicates that Bcat protein abundance is lower than that of BCKDH components in one-week-old adult flies (Supplementary Fig. 6b).

Because starvation-induced changes in BCAA levels were more pronounced in males, we focused subsequent experiments on male flies. *Bcat* expression was strongly upregulated during starvation, but this induction was significantly attenuated in *AkhR* mutants (Fig. 4d), indicating that starvation-dependent Bcat induction is partly mediated by Akh signaling. Consistently, ectopic expression of *Akh* in the fat body further increased *Bcat* expression during starvation, but not under fed conditions (Fig. 4e). By contrast, *Bckdhb* and *Dbct*, but not *Bckdha*, were modestly upregulated by starvation (Fig. 4f), and *AkhR* mutants failed to induce starvation-dependent *Bckdhb*. However, *Akh* overexpression alone was not sufficient to induce *Bckdhb* (Supplementary Fig. 6c), suggesting that Akh signaling is not a primary driver of BCKDH transcription under these conditions. Notably, ex vivo incubation of abdominal carcasses (devoid of thoracic muscle) with Akh peptide increased *Bcat* expression (Fig. 4g). Together, these results support a model in which Akh signaling acts on abdominal tissues to promote Bcat induction during starvation, whereas BCKDH component expression is comparatively stable at the transcriptional level.

While BCAA levels rise in control flies during starvation, this is accompanied by an increase in *Bcat* expression, suggesting that BCAA supply exceeds their catabolic capacity. To test this, we analyzed viable *Bcat* hypomorphic mutants and ubiquitous *Bcat* knockdown flies. These flies showed elevated BCAA levels under fed conditions, with further increases upon starvation (Fig. 4h, Supplementary Fig. 6d), suggesting active BCAA supply during nutrient deprivation. Notably, *Bcat* knockdown in the fat body significantly increased BCAA levels during starvation but had no effect under fed conditions (Fig. 4i), indicating that fat body Bcat contributes to systemic BCAA clearance during starvation. In contrast, *Bcat* knockdown in muscle increased BCAA levels under fed conditions, with only a slight increase under starvation (Fig. 4j). These results suggest that Bcat in the fat body and muscle differentially regulate systemic BCAA levels depending on nutrient status.

### Fat body BCAA catabolism shapes Akh output during starvation

**Fig. 5:**
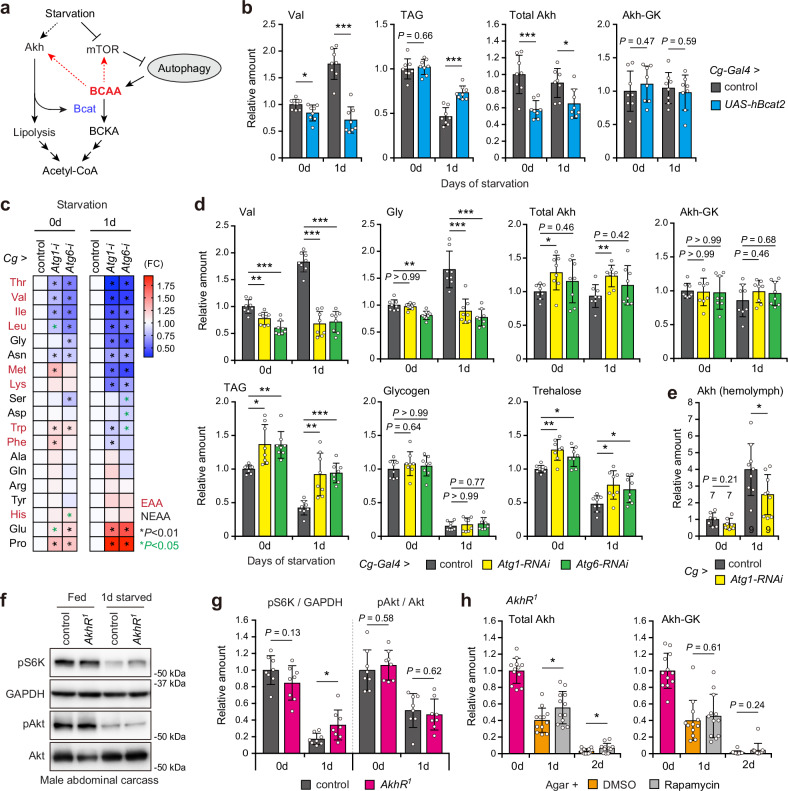
Fat body BCAA catabolism shapes Akh output during starvation. **a** Simplified working model illustrating Akh function in lipolysis and BCAA catabolism in the fat body. Starvation-induced autophagy releases BCAAs, which promotes Akh secretion and can reactivate mTOR to dampen autophagy. Akh-induced Bcat promotes BCAA catabolism, generating branched-chain keto acids (BCKAs) that are further processed into downstream metabolites such as acetyl-CoA, thereby forming negative feedback from the fat body to CC cells. **b** Relative amounts of indicated metabolites and Akh peptides during starvation with fat body-specific overexpression of human Bcat2 (*hBcat2*). 0 d, fed; 1 d, 1 day starved. **c** Heatmaps showing amino acid levels in flies with fat body-specific knockdown of *Atg1* or *Atg6* during starvation. Red and blue indicate increased or decreased metabolites relative to control value at each time point. *Atg1-i*, *Atg1-RNAi*; *Atg6-i*, *Atg6-RNAi*. **d** Relative amounts of indicated metabolites and Akh peptides during starvation. **e** Circulating Akh levels in hemolymph of control and *Atg1* knockdown flies during starvation. **f** Immunoblots showing phospho-S6K (pS6K) and phospho-Akt (pAkt) in control and *AkhR* mutants. Male abdominal carcasses were dissected and used for western blotting. GAPDH and total Akt were used as loading controls. **g** Quantification of relative pS6K and pAkt levels normalized to GAPDH and total Akt, respectively. **h** Relative amounts of Akh peptides in *AkhR* mutants during starvation with rapamycin feeding. Results are presented as mean ± SD (**b,** **d,** **e,** **g,** **h**); *n* = 8 (**b**–**d**,**g**) or 12 (**h**) batches. Values of n indicate the number of batches (**e**). Statistical tests: unpaired two-tailed Welch’s *t*-test (**b**,**e**), unpaired two-tailed Welch’s *t*-test with Bonferroni correction (**c**,**d**), unpaired two-tailed Student’s *t*-test (**g**,**h**); *p < 0.05; **p < 0.01; ***p < 0.001 (except in **c**). Source data are provided as a Source Data file, which also includes exact *P* values.

We hypothesized that circulating BCAAs act as a metabolic cue for Akh secretion, while Akh signaling in the fat body counterbalances this effect by promoting BCAA clearance, thereby maintaining metabolic homeostasis through inter-organ feedback (Fig. 5a). To test this, we next asked whether enhancing BCAA catabolism in a wild-type background is sufficient to dampen Akh pathway output. Expression of human Bcat2 (*hBcat2*) in the fat body significantly reduced whole-body BCAA levels, with a stronger effect under starvation (Fig. 5b). Under these conditions, starvation-induced lipolysis was significantly suppressed, consistent with reduced Akh signaling. However, fat body hBCAT2 overexpression also markedly reduced basal total Akh levels, presumably due to chronically lowered BCAA availability, complicating interpretation of the starvation phenotype specifically in terms of secretion.

During starvation, free amino acids are primarily supplied through autophagy and protein degradation^45^. To test whether autophagy contributes to BCAA availability and Akh-mediated lipolysis, we knocked down the autophagy genes *Atg1* and *Atg6* in the fat body. Knockdown of these genes altered amino acid profiles even under ad libitum feeding (Fig. 5c, d). However, the effects were more pronounced under starvation: levels of many EAAs, including BCAAs, were significantly reduced. Interestingly, inhibition of autophagy increased TAG and trehalose, but not glycogen, under both fed and starved conditions (Fig. 5d), partially recapitulating the phenotype observed in *AkhR* mutants. Moreover, while total Akh levels were slightly increased upon inhibition of fat body autophagy, circulating Akh levels were significantly reduced during starvation (Fig. 5d, e). Together, these data support a model in which fat body autophagy contributes to the starvation-induced rise in BCAA levels, which in turn promotes Akh secretion and downstream lipolysis (Fig. 5a).

Autophagy-derived amino acids can contribute to mTORC1 reactivation, thereby forming a negative-feedback loop that limits autophagy during prolonged starvation^46^. We therefore examined whether increased BCAAs affect mTOR activity in *AkhR* mutants. In control flies, S6K phosphorylation in abdominal carcasses was significantly decreased upon starvation (Fig. 5f, g), indicating reduced mTOR activity. In contrast, *AkhR* mutants exhibited higher pS6K levels than control flies under starved conditions, although these levels remained lower than in fed conditions. Notably, pAkt levels in *AkhR* mutants were comparable to those in control flies. These results suggest that mTOR activity is partially maintained in *AkhR* mutants during starvation, most likely reflecting elevated BCAA levels rather than changes in insulin/PI3K signaling.

Because mTOR could, in principle, provide a mechanistic link between elevated amino acids and Akh secretion, we next performed rapamycin-feeding experiments to acutely suppress mTOR activity during starvation. Rapamycin feeding only slightly suppressed depletion of total Akh levels during starvation in *AkhR* mutants, yet these levels were depleted almost completely after two days of starvation (Fig. 5h). Thus, under our experimental conditions, mTOR inhibition alone does not appear sufficient to account for the Akh hypersecretion phenotype in *AkhR* mutants.

### BCAA catabolism in the fat body remotely suppresses Akh secretion

**Fig. 6:**
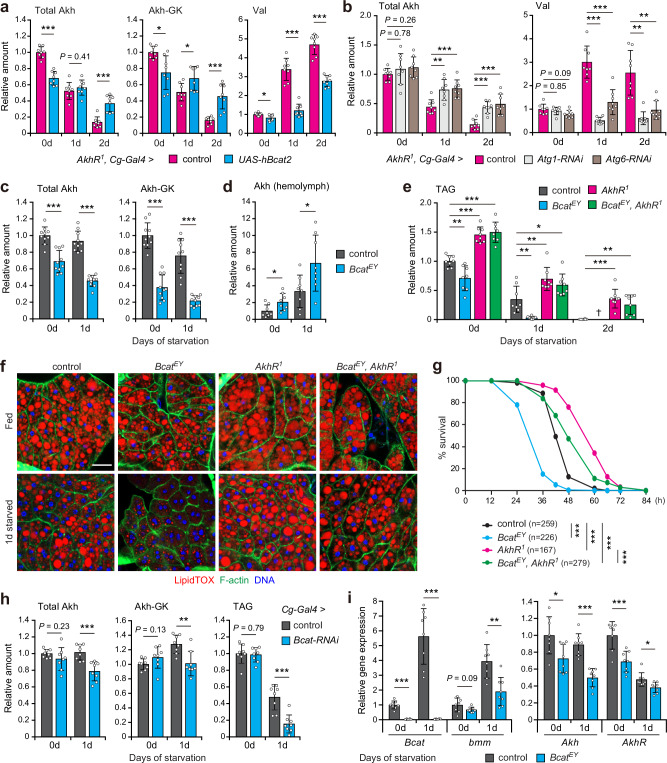
BCAA catabolism in the fat body remotely suppresses Akh secretion. **a** Relative amounts of total Akh, Akh-GK, and valine in *AkhR* mutants during starvation with fat body-specific overexpression of human *Bcat2* (*hBcat2*). 0 d, fed; 1 d, 1 day starved; 2 d, 2 days starved. **b** Relative amounts of total Akh and valine in *AkhR* mutants during starvation with autophagy inhibited in the fat body. **c** Relative amounts of total Akh and Akh-GK in control and *Bcat* mutants. **d** Circulating Akh levels in hemolymph of control and *Bcat* mutants. **e** Relative amounts of TAG in the indicated genotypes during starvation. **f** Lipid storage in the fat body visualized by LipidTOX staining. Abdominal carcasses were dissected and stained for LipidTOX, F-actin, and DNA. Representative images are shown. Scale bar: 20 μm. **g** Survival curves during starvation for the indicated genotypes. **h** Relative amounts of total Akh, Akh-GK, and TAG in fat body-specific *Bcat* knockdown flies. **i** Relative gene expression levels in *Bcat* mutants during starvation. Results are presented as mean ± SD (**a**–**e**,**h**,**i**); *n* = 8 (**a**,**b**,**d**,**e**,**h**,**i**) or 10 (**c**) batches. Values of n indicate the number of flies (**g**). Statistical tests: unpaired two-tailed Welch’s *t*-test (**a**,**d**), unpaired two-tailed Welch’s *t*-test with Bonferroni correction (**b**), unpaired two-tailed Student’s *t*-test (**c**,**h**,**i**), one-way ANOVA with Dunnett’s post hoc test (**e**), log-rank test with Bonferroni correction (**g**); *p < 0.05; **p < 0.01; ***p < 0.001. Source data are provided as a Source Data file, which also includes exact *P* values.

Our data collectively suggest that BCAA levels correlate with Akh secretion during starvation, with increased BCAAs associated with enhanced secretion, and decreased levels linked to reduced Akh secretion. To examine the functional contribution of BCAA metabolism to Akh secretion, we manipulated BCAA levels in *AkhR* mutants. hBCAT2 overexpression in the fat body of *AkhR* mutants reduced BCAA levels under both fed and starved conditions (Fig. 6a). Importantly, *hBcat2* expression largely prevented Akh depletion during starvation, albeit with lower basal Akh levels. Similarly, fat body-specific knockdown of *Atg1* or *Atg6* in *AkhR* mutants significantly, but not completely, suppressed Akh depletion during starvation (Fig. 6b, Supplementary Fig. 7a). These manipulations also normalized starvation-dependent accumulation of BCAA levels. While BCAA levels alone do not fully explain Akh depletion, these findings support a positive regulatory role of BCAAs in Akh secretion.

If elevated BCAAs promote Akh secretion, *Bcat* mutants should display enhanced Akh signaling and starvation sensitivity. Indeed, *Bcat* mutants showed reduced basal Akh levels, with further decline upon starvation in whole-body samples (Fig. 6c). In contrast, circulating Akh was elevated in *Bcat* mutants, consistent with increased Akh secretion (Fig. 6d). This enhanced secretion led to decreased TAG stores under fed conditions and accelerated TAG depletion during starvation, resulting in starvation sensitivity (Fig. 6e–g). These phenotypes were largely rescued by introducing *AkhR* mutations, indicating that enhanced lipolysis and starvation sensitivity in *Bcat* mutants are Akh-dependent. Similar phenotypes, including reductions of total Akh and TAG levels during starvation, were observed in ubiquitous *Bcat* knockdown flies, as well as in fat body-specific knockdown flies (Fig. 6h, Supplementary Fig. 7b). In contrast, CC-specific *Bcat* knockdown did not affect Akh levels (Supplementary Fig. 7c), suggesting that Bcat influences Akh secretion in a non-cell-autonomous manner. Interestingly, the expression of *Akh*, *AkhR*, and *bmm* was reduced in *Bcat* mutants (Fig. 6i), likely reflecting adaptive responses to excessive lipolysis. These findings demonstrate that BCAA catabolism in the fat body remotely suppresses Akh secretion from the CC, acting as a key metabolic feedback mechanism.

### BCAA catabolism links to glutathione synthesis and redox homeostasis

What is the physiological significance of Akh in promoting BCAA catabolism in the fat body during starvation? BCAA catabolism is intimately associated with the production of NEAAs and the critical antioxidant GSH via glutamate^47^. Consequently, defective BCAA catabolism has been shown to increase oxidative stress in several organisms^48,49^. Akh is required for resistance to oxidative stress in several insects, including *Drosophila*^9,28^; however, the underlying mechanisms remain elusive.

**Fig. 7:**
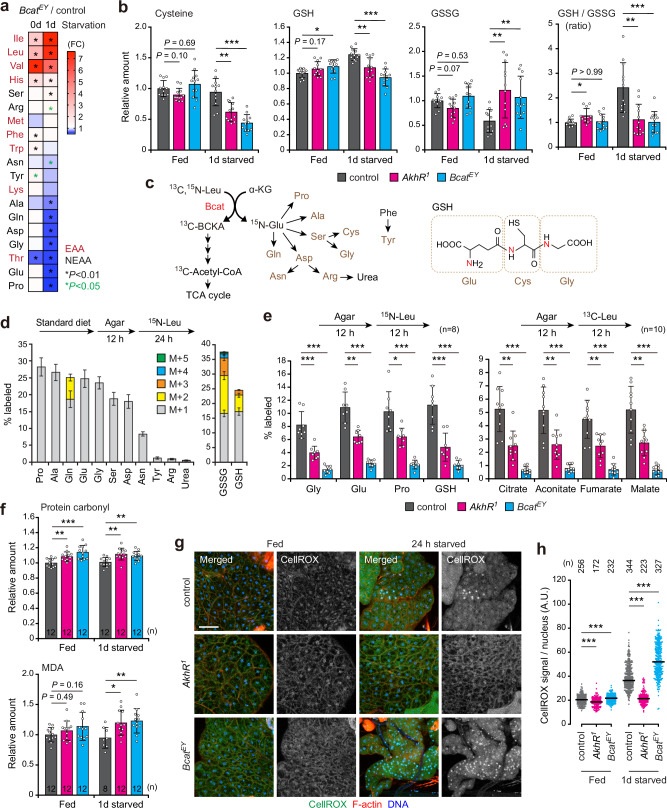
BCAA catabolism links to glutathione synthesis and redox homeostasis. **a** Heatmap of amino acid levels in *Bcat* mutants during starvation. Red and blue indicate increased and decreased metabolites relative to the control value at each time point. FC, fold change; EAAs, essential amino acids; NEAAs, non-essential amino acids. 0 d, fed; 1 d, 1 day starved. **b** Relative amounts of indicated metabolites and GSH/GSSG ratio in *AkhR* and *Bcat* mutants during starvation. **c** Simplified schematic of leucine catabolism mediated by Bcat and downstream metabolic pathways leading to glutathione production. A schematic illustration of the tripeptide GSH is shown on the right, with ^15^N-labeled nitrogen atoms highlighted in red. α-KG, α-ketoglutarate; BCKA, branched-chain keto acids. **d** Percent isotope labeling of metabolites in control flies fed 2 mM ^15^N-Leu for 24 h. **e** Percent isotope labeling of metabolites in *AkhR* and *Bcat* mutants fed 1 mM ^15^N-Leu or 1 mM ^13^C-Leu for 12 h. **f** Relative amounts of protein carbonyls and MDA in *AkhR* and *Bcat* mutants during starvation. **g**,**h** Oxidative conditions in the fat body measured with the oxidation-sensitive fluorescence probe CellROX. Abdominal carcasses of the indicated genotypes were stained for CellROX, F-actin, and DNA (**g**). Representative images are shown. Scale bar: 40 μm. Quantification of CellROX signal intensity per nucleus is shown in **h**, where horizontal lines represent group means. Results are presented as mean ± SD (**b**,**d**–**f**); *n* = 8 (**a**), 12 (**b**), or 7 (**d**) batches. Values of n indicate the number of batches (**e,** **f**) or nuclei (**h**) from multiple flies. Statistical tests: unpaired two-tailed Welch’s *t*-test (**a**), unpaired two-tailed Welch’s *t*-test with Bonferroni correction (**b**,**e**,**f**), Kruskal-Wallis test followed by Dunn’s *post hoc* test (**h**); *p < 0.05; **p < 0.01; ***p < 0.001 (except in **a**). Source data are provided as a Source Data file, which also includes exact *P* values.

To investigate the importance of BCAA catabolism, we analyzed amino acid profiles in *Bcat* mutants. While BCAAs were drastically increased in *Bcat* mutants, several NEAAs, such as proline, glutamate, glycine, aspartate, and glutamine, were significantly decreased only under starvation conditions (Fig. 7a, Supplementary Fig. 8a). Notably, glycine levels increased during starvation in control flies, but were most strongly reduced amino acid in *AkhR* mutants following starvation (Fig. 4a, b). These results support the hypothesis that BCAA catabolism regulated by Akh signaling is required to maintain NEAA levels during starvation.

The tripeptide GSH (γ-L-glutamyl-L-cysteinyl-glycine) consists of glutamate, cysteine, and glycine. While cysteine levels did not change during starvation in control flies, they significantly decreased in both *AkhR* and *Bcat* mutants (Fig. 7b). In control flies, GSH levels slightly but significantly increased during starvation, whereas oxidized GSH (GSSG) levels decreased, resulting in an elevated GSH/GSSG ratio. In contrast, both *AkhR* and *Bcat* mutants displayed decreased GSH and increased GSSG levels during starvation, leading to a lower GSH/GSSG ratio. These changes were not observed under fed conditions, suggesting that defects in GSH synthesis and redox homeostasis are specifically induced by starvation.

To directly examine whether BCAAs can contribute to GSH synthesis during starvation, we performed stable isotope tracing using ^15^N-labeled leucine (Fig. 7c). Transient feeding of ^15^N-leucine during starvation led to incorporation of the label into several NEAAs as well as into GSH and GSSG (Fig. 7d), indicating that leucine can serve as a nitrogen donor for NEAA and GSH synthesis under the experimental conditions used for isotope tracing. Importantly, ^15^N labeling in arginine and urea was limited under these conditions, suggesting that BCAA-derived nitrogen is not predominantly routed through arginine/urea-related pathways but instead contributes to NEAA and GSH synthesis. The incorporation of ^15^N into NEAAs and GSH was reduced in *AkhR* mutants and was further diminished in *Bcat* mutants (Fig. 7e), suggesting impaired BCAA-dependent GSH biosynthesis. Consistently, the expression of GSH synthesis genes was also reduced in both *AkhR* and *Bcat* mutants (Supplementary Fig. 8b), reinforcing the role of Akh signaling in promoting GSH biosynthesis. Furthermore, ^13^C-labeled leucine tracing revealed that the fraction of ^13^C-labeled TCA cycle intermediates was decreased in *AkhR* mutants and was more severely reduced in *Bcat* mutants (Fig. 7e). Together, these results provide functional support for the conclusion that Akh-dependent regulation of *Bcat* expression is important for overall BCAA catabolism during starvation.

To further assess redox homeostasis in the context of altered BCAA catabolism, we measured oxidative stress markers in *AkhR* and *Bcat* mutants. *AkhR* mutants showed significantly elevated levels of protein carbonyls and the lipid peroxidation marker malondialdehyde (MDA), particularly during starvation (Fig. 6f). Similar increases were observed in *Bcat* mutants, indicating that Akh signaling and BCAA catabolism impact systemic redox balance. Consistently, fat body staining with CellROX, an oxidation-sensitive fluorescence probe, showed that *Bcat* mutants exhibited significantly higher signals during starvation, while CellROX signals also increased in control flies upon starvation (Fig. 6g, h). Notably, *Bcat* mutants showed slightly elevated CellROX signals even under fed conditions, suggesting that Bcat is required for maintaining redox homeostasis. Unexpectedly, *AkhR* mutants exhibited lower CellROX signals under fed conditions and showed only a marginal increase during starvation, highlighting that tissue-level oxidation readouts do not necessarily mirror whole-organism oxidative damage markers.

To better define the differences between the two genotypes, we analyzed the expression of oxidative stress-related genes. Under fed conditions, *AkhR* mutants showed decreased expression of all superoxide dismutases (*Sod1–3*) and catalase (*Cat*), whereas under starvation only *Sod2* was significantly decreased (Supplementary Fig. 8c). In contrast, *Bcat* mutants exhibited a stronger and broader reduction of antioxidant gene expression: *Sod2*, *Sod3*, and *Cat* were significantly decreased under both fed and starved conditions. In line with this, *Bcat* mutants strongly up-regulated *GstD1* and *GstD2*, canonical oxidative stress-response genes. Together, these expression patterns suggest that *Bcat* mutants experience a more pronounced impairment in antioxidant capacity and oxidative stress responses than *AkhR* mutants. Given that Akh signaling regulates metabolism and behavioral responses to starvation^8–12^, the redox phenotype of *AkhR* mutants may reflect a combination of altered antioxidant gene regulation and reduced endogenous reactive oxygen species (ROS) production associated with decreased overall metabolic activity.

### Diet- and stage-dependent regulation of Akh secretion and feedback

**Fig. 8:**
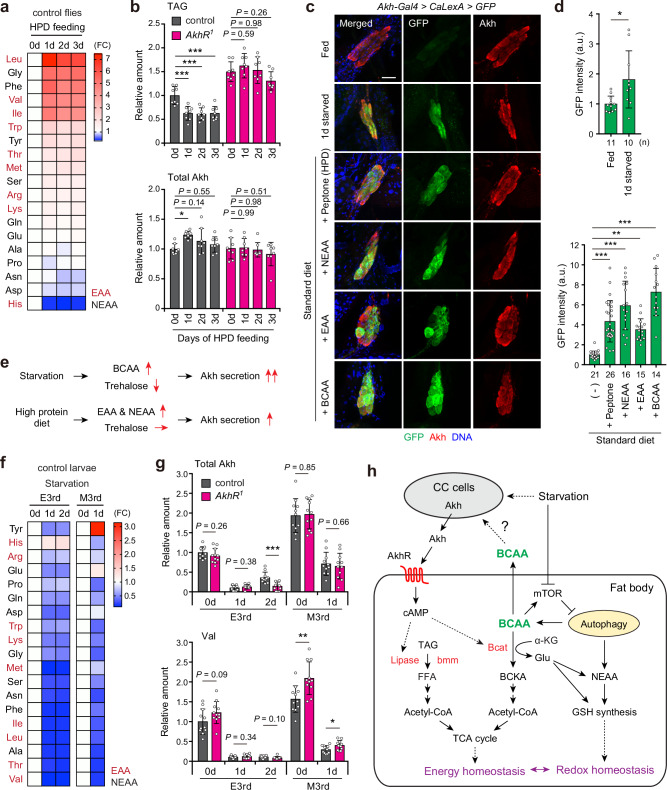
Diet- and stage-dependent regulation of Akh secretion and feedback. **a** Heatmap of amino acid levels in control flies during high-protein diet (HPD) feeding. Red and blue indicate increased or decreased metabolites relative to 0 day (0 d). FC, fold change; EAAs, essential amino acids; NEAAs, non-essential amino acids. **b** Relative amounts of TAG and total Akh in control and *AkhR* mutants during HPD feeding. **c,** **d** Calcium activity in CC cells measured using the GFP-based CaLexA reporter after 1-day starvation or feeding on a standard diet supplemented with peptone (HPD) or amino acids. Representative GFP, Akh, and DNA images are shown in (**c**). Quantification of mean GFP fluorescence intensity per CC cell cluster is shown in (**d**). Scale bar: 20 μm. **e** Simplified schematic of how amino acid and trehalose levels regulate Akh secretion under starvation or HPD feeding. **f** Heatmaps of amino acid levels in control larvae during starvation. Red and blue indicate increased or decreased metabolites relative to 0 d at each larval stage. E3rd, early third instar; M3rd, mid third instar (24 h after second ecdysis). **g** Relative amounts of total Akh and valine in control and *AkhR* mutant larvae during starvation. **h** Proposed model of inter-organ communication between CC and the fat body via BCAAs. During starvation, Akh promotes lipolysis and the breakdown of autophagy-derived BCAAs. BCAA catabolism supports energy and redox homeostasis by maintaining TCA cycle flux and glutathione synthesis, partly through NEAA production. Increased BCAAs are proposed to promote Akh secretion, although the site and mechanism remain unresolved. Akh signaling and bmm act in parallel and compensate for each other to support lipid mobilization during starvation. Results are presented as mean ± SD (**b**,**d**,**g**); *n* = 8 (**a**,**b**), 11 (**f**,**g**) batches; n indicates fly number (**d**). Statistical tests: one-way ANOVA with Dunnett’s post hoc test (**b**), two-sided Mann–Whitney U-test or Kruskal-Wallis test followed by Dunn’s *post hoc* test (**d**), unpaired two-tailed Welch’s *t*-test (**g**); *p < 0.05; **p < 0.01; ***p < 0.001. Source data are provided as a Source Data file, which also includes exact *P* values.

Recent studies have reported that several amino acids can activate CC cells in larvae^50^. However, how adult CC cells respond to amino acids remains unclear. To address this, we tested whether elevated amino acid levels are sufficient to promote Akh secretion in adults. Transient feeding with a high-protein diet (HPD) markedly increased amino acid levels, particularly EAAs including BCAAs, in control flies (Fig. 8a). *AkhR* mutants displayed several alterations in amino acid levels following HPD feeding; however, the increase in BCAA levels was comparable to that in controls (Supplementary Fig. 9a, b). HPD caused a sustained reduction in TAG stores from day 1 onward without further decreases, whereas TAG levels in *AkhR* mutants remained unchanged (Fig. 8b). Notably, total Akh levels remained largely unchanged overall, whereas Akh-GK levels showed a slight increasing trend in both control and *AkhR* mutants following HPD feeding (Fig. 8b, Supplementary Fig. 9c). These results suggest that HPD-induced lipolysis requires Akh signaling, whereas excessive Akh secretion does not occur in *AkhR* mutants.

To visualize CC cell activity in response to dietary changes, we used CaLexA, an NFAT-based calcium-dependent GFP sensor^51^. GFP expression in CC cells was upregulated by one day starvation (Fig. 8c, d). Similarly, HPD feeding for one day significantly upregulated GFP expression, consistent with Akh-dependent lipolysis. Furthermore, supplementation with NEAAs, EAAs, or BCAAs each triggered CC cell activation, suggesting that CC cell activity is not exclusively responsive to BCAAs but is broadly activated by elevated amino acid availability. Together, these results suggest that BCAAs mediate physiologically relevant regulation of Akh secretion during starvation, whereas multiple amino acids can trigger Akh secretion during HPD feeding (Fig. 8e). Given that HPD did not reduce trehalose levels in either control or *AkhR* mutants (Supplementary Fig. 9c), Akh hypersecretion in *AkhR* mutants likely requires a combination of decreased carbohydrate levels and elevated BCAA levels.

We examined amino acid profiles and Akh dynamics during starvation in larvae. In contrast to adults, starvation markedly decreased levels of many amino acids, including BCAAs, in control larvae (Fig. 8f). Similar reductions were observed at both early and mid-third instar stages (i.e., before and after pupal commitment). *AkhR* mutant larvae showed altered amino acid profiles, partially recapitulating those observed in adults, such as increased EAAs and decreased NEAAs (Supplementary Fig. 9a). However, total Akh levels in whole larvae significantly decreased during starvation in both control and *AkhR* mutants (Fig. 8g). These results suggest that larval Akh secretion is not regulated by increased amino acid levels, nor is it modulated by AkhR-dependent feedback during starvation.

Together, these results raise the possibility that the BCAA-linked regulation of Akh secretion and lipolysis is primarily an adult-stage phenomena. To test whether these phenotypes arise during adulthood rather than development, we analyzed newly eclosed flies. In newly eclosed *AkhR* mutants, total protein, total Akh, and Akh-GK levels were comparable to controls (Supplementary Fig. 10a), suggesting that the decreased total Akh levels and increased Akh-GK levels observed in one-week-old adults (Fig. 2a) arise during adulthood. Similarly, newly eclosed *Bcat* mutants showed normal total protein and Akh-GK levels, whereas total Akh was elevated (Supplementary Fig. 10a); this contrasts with the marked reduction of total Akh and Akh-GK in adults (Fig. 6c), again supporting an adult-onset phenotype. Notably, TAG levels in newly eclosed *AkhR* and *Bcat* mutants were indistinguishable from controls (Supplementary Fig. 10a), indicating that the increased TAG in *AkhR* mutants and decreased TAG in *Bcat* mutants observed in adults (Fig. 6e) represent adult-onset metabolic phenotypes. BCAA levels were already elevated in newly eclosed *Bcat* mutants (Supplementary Fig. 10a), suggesting that elevated BCAAs alone are not sufficient to alter TAG levels during development. Together, these results support our conclusion that the AkhR-dependent feedback regulation is primarily adult-specific. Importantly, CC cell number and cell size in *AkhR* and *Bcat* mutants were indistinguishable from controls in one-week-old adults (Fig. S10b, c), indicating that these genotypes do not cause detectable developmental defects in CC cells.

Lastly, we performed ex vivo calcium imaging in adult CC cells to test whether BCAAs directly evoke an acute activation signal. While high K^+^ robustly increased GCaMP signals, we detected no significant calcium response to elevated BCAAs under our adult ex vivo CC imaging conditions (Supplementary Fig. 11a–d). We also detected no significant responses to glycine or threonine, which have been reported to stimulate larval CC cells^50^. These results indicate that, under our assay conditions, adult CC cells do not show an acute calcium response to these amino acids. This difference could reflect stage-specific properties of CC cells and/or limitations of the current ex vivo conditions for capturing amino-acid-evoked responses in adults.

In conclusion, our study reveals that Akh signaling not only responds to energy demands but also orchestrates amino acid catabolism and redox homeostasis via inter-organ feedback, highlighting its pivotal role in metabolic adaptation during starvation (Fig. 8h).

## Discussion

Akh signaling is a central regulator of energy mobilization and systemic physiology in insects^8,12^. While genetic and physiological studies have highlighted its diverse roles, direct measurement of Akh secretion has remained technically challenging. We here established a sensitive LC-MS/MS-based assay for quantifying both mature Akh and its precursors. Our results indicate that more than 99% of Akh detected in adult flies is stored within the CC, with only a small fraction present in the hemolymph, even under starvation. By integrating this Akh quantification system with *Drosophila* genetics, we demonstrated that *AkhR* mutants exhibit a marked reduction in both mature and precursor Akh peptides during starvation, likely due to hypersecretion followed by degradation. Our rationale for using *AkhR* mutants throughout the study was to unmask the physiological negative-feedback architecture that operates in wild-type flies. In wild-type conditions, the feedback loop between the fat body and CC cells buffers both Akh secretion and downstream metabolite levels, making it difficult to observe large effect sizes. In line with the idea that secreted Akh is rapidly cleared from circulation, we note that glucagon-like peptide 1 (GLP1), a product of the proglucagon gene in mammals, is rapidly degraded by the protease DPP-4 and exhibits a short half-life in circulation^52^. Although the half-life of Akh in insect hemolymph remains unknown, it is plausible that Akh is similarly degraded by extracellular peptidases within a defined time window.

Previous work has suggested that starvation triggers Akh secretion via decreased sugar availability, sensed through ATP-sensitive potassium channels in larval CC cells^13,21,22^. Starvation also induces autophagy, which mobilizes amino acids to maintain tissue integrity and energy balance. Given that the levels of several amino acids, particularly BCAAs, increase in an autophagy-dependent manner in the fat body, we propose a model in which amino acid signals, together with decreased circulating sugars, cooperatively regulate adult Akh secretion during starvation. The increase in BCAAs was more rapid and apparent in wild-type males than in females, consistent with previous reports showing higher Akh secretion and lipolysis activity in males^53,54^. We note that changes in whole-body amino-acid abundances reflect the net balance between autophagy-driven production and metabolic utilization; therefore, similar qualitative trends in pool sizes do not necessarily exclude genotype-dependent differences in catabolic flux. We showed that amino acid levels strongly correlate with feeding status in larvae, whereas they remain relatively stable in adults, suggesting that amino acid homeostasis is regulated differently between developmental stages, presumably reflecting the distinct physiological priorities of growth versus reproduction and maintenance. In addition, Akh signaling acts in the nervous system to modulate starvation-associated behaviors (e.g., activity/foraging)^10,11^, and thus whole-body metabolic phenotypes in *AkhR* mutants can reflect both tissue-autonomous metabolic changes and indirect behavioral effects.

Our results suggest that defects in BCAA clearance mediated by Akh signaling result in excessive Akh secretion. Although genetic manipulation of BCAA metabolism affected Akh secretion, changes in BCAA levels alone could not fully account for the severe reduction in Akh levels observed in *AkhR* mutants. We also found sex-specific differences in carbohydrate metabolism in *AkhR* mutants, which potentially influence Akh secretion. Thus, multiple nutrient-derived signals appear to regulate Akh secretion to prevent excessive hormonal action during starvation in a sex-dependent manner. Moreover, the relative contribution of individual amino acids to Akh secretion likely depends on both the nutritional context and developmental stage. In fact, BCAAs have only minor effects on larval CC activation compared to other amino acids^50^. In mammals, glucagon secretion from α-cells is regulated by multiple signals, including glucose, free fatty acids, ketone bodies, and amino acids. However, the role of individual nutrient signals remains controversial^6^. For example, in vitro studies suggest that fatty acids promote glucagon secretion via GPCR-mediated signaling^55–57^, whereas some clinical studies have reported that elevated plasma fatty acids suppress glucagon secretion^58^. Similarly, several amino acids such as arginine, alanine, and glutamine can stimulate glucagon release^6^. BCAAs have recently emerged as potent activators of glucagon secretion in diabetic models, while their stimulatory effects are undetectable in non-diabetic mice^59^. BCAA catabolism by Bcat2 within diabetic α-cells is responsible for BCAA-induced hypersecretion of glucagon. In contrast, our results suggest that Bcat in CC cells does not contribute to Akh hypersecretion in *AkhR* mutants. Together, these observations suggest that Akh secretion in insects is governed by redundant, stage-specific, and context-dependent mechanisms, paralleling the complex regulatory logic of mammalian glucagon secretion. This complexity likely reflects the necessity for precise regulation of catabolic hormones to maintain metabolic homeostasis and survival during nutritional challenge.

Our ex vivo imaging results do not support acute BCAA induced calcium activation in adult CC cells under the conditions tested. We note that ex vivo calcium imaging has been extensively performed in larval CC cells^13,24,43,50,60^, whereas there are no established protocols or published studies demonstrating robust stimulus-evoked calcium responses in adult CC cells. Because CC cell responses to starvation are likely stage-dependent, the absence of detectable amino acid induced calcium responses in our adult ex vivo assay could reflect stage-specific properties of CC cells and/or limitations of the current assay conditions. Importantly, the absence of an acute calcium signal does not by itself rule out direct regulation via other signaling modalities. In mammals, several amino acids such as arginine and glutamine can stimulate hormone secretion, potentially through effects on membrane potential or mTOR signaling^61,62^. Yet, the precise molecular mechanisms underlying amino acid-triggered hormone release remain poorly understood. Further studies will be required to fully understand the molecular and cellular mechanisms by which BCAAs modulate Akh secretion, including the identification of nutrient sensors and downstream signaling cascades. In addition, our data are consistent with an indirect route in which BCAAs influence Akh secretion via intermediate metabolic and/or neural pathways.

We demonstrated that Akh signaling regulates amino acid catabolism, although the magnitude of amino acid changes in *AkhR* mutants is relatively modest compared to those observed in glucagon receptor (*Gcgr*) knockout mice^18,19,63^. In particular, starved *AkhR* mutants exhibit increases in all three BCAAs, consistent with a failure to upregulate *Bcat* expression. Although Bcat-catalyzed transamination is freely reversible in vitro, our data suggest that starvation-induced upregulation of *Bcat* increases the capacity of the first step of BCAA catabolism and promotes BCAA catabolic flux through downstream BCKDH-dependent steps. In mammals, glucagon signaling through the hepatic glucagon receptor upregulates the expression of multiple amino acid transporters and enzymes involved in amino acid catabolism^18–20^. Akh signaling may similarly regulate a broader set of amino acid metabolic genes during starvation. Identifying the signaling and transcriptional components connecting AkhR activation to *Bcat* expression will be an important direction for future work.

The differences between flies and mammals in glucagon/Akh-dependent amino acid regulation likely reflect tissue specialization. In mammals, the liver is the main glucagon target organ, but it expresses only low levels of Bcat and contributes minimally to BCAA oxidation^64,65^. Instead, skeletal muscle and adipose tissue serve as the major sites of BCAA catabolism. Accordingly, BCAA elevation in *Gcgr* knockout mice is relatively mild compared to changes in other amino acids. In contrast, the *Drosophila* fat body performs the dual functions of both mammalian hepatocytes and adipocytes. Thus, the observed species-specific differences in amino acid metabolism reflect the distinct metabolic architecture of the liver versus the fat body.

Akh-dependent control of amino acid catabolism may also interface with lipid mobilization pathways. Fat body lipolysis is regulated by two distinct mechanisms: Akh-dependent signaling and the starvation-induced lipase bmm pathway^53,66^. Although these systems function redundantly, they also communicate in a compensatory manner to ensure lipid mobilization^15^. Interestingly, *bmm* knockdown in the fat body specifically increases all three BCAAs in starved flies^67^. While it remains uncertain whether BCAA accumulation in *bmm* mutants is dependent on impaired Akh signaling, our findings support the notion that increased BCAA levels stimulate Akh secretion to compensate for defective lipolysis. Further investigation will be required to unravel the mechanistic crosstalk between lipolysis and amino acid catabolism in coordinating energy mobilization during starvation.

Numerous studies in insects have demonstrated that Akh confers protection against oxidative stress, at least in part via the transcription factor FoxO^28,68^. Our findings provide an additional mechanism by which Akh promotes redox homeostasis through induction of BCAA catabolism, thereby supporting the synthesis of NEAAs and GSH. These results do not exclude additional nitrogen donors that could also support NEAA synthesis and redox buffering. Because lipolysis-induced β-oxidation generates mitochondrial ROS, maintaining redox balance becomes particularly critical during prolonged starvation, when exogenous antioxidants are unavailable. In this context, the lack of a starvation-induced increase in the fat body CellROX signal in *AkhR* mutants may, at least in part, reflect reduced lipolysis-driven ROS production, whereas the elevated ROS in *Bcat* mutants is consistent with impaired redox-buffering capacity in combination with elevated lipolysis due to excess Akh secretion. Thus, the coordinated regulation of lipolysis and GSH biosynthesis by Akh signaling represents a physiologically relevant adaptation that limits oxidative stress during nutrient deprivation.

In mammals, glucagon signaling promotes amino acid catabolism and subsequent ureagenesis in the liver. In contrast, our data show that nitrogen derived from BCAAs is redirected toward NEAA and GSH synthesis rather than urea or arginine production. However, it is possible that Akh signaling also facilitates nitrogen disposal via ureagenesis under specific dietary conditions such as HPD feeding. In mammals, the liver is a major regulator of inter-organ GSH homeostasis and serves as a primary source of circulating GSH^69^. This raises the possibility that glucagon, like Akh, modulates redox homeostasis through GSH metabolism, balancing catabolic output with oxidative stress management in a nutrient-dependent manner.

In conclusion, our study reveals that Akh-BCAA axis acts as a metabolic integrator that coordinates amino acid metabolism, redox homeostasis, and inter-organ communication during starvation. The identification of a feedback mechanism linking Akh signaling in the fat body to Akh secretion via BCAA metabolism provides new insight into how nutrient sensing and hormonal output are balanced under nutrient stress. These findings establish a conceptual framework for understanding endocrine regulation of metabolism in insects and suggest potential parallels with mammalian glucagon systems.

## Methods

### Drosophila strains

The following *Drosophila melanogaster* strains were used: *w*^*1118*^ (used as a control), *Akh*^*A*^, *AkhR*^*1*^, *UAS-bmm* (gifts from Dr. Ronald P. Kühnlein), and *UAS-His2A-mRFP*^70^. The following stocks were obtained from the Bloomington *Drosophila* stock center (BDSC): *Tub-Gal4* (5138), *UAS-Kir2.1* (6596), *Cg-Gal4* (7011), *Tub-Gal80ts* (7108), *repo-Gal4* (7415), *elav-Gal4* (8765), *P{EPgy2}Bcat*^*EY20842*^ (23109)*, Akh-Gal4* (25684), *AMPKα-RNAi* (25931), *Mef2-Gal4* (27390), *r4-Gal4* (33832), *GFP-RNAi* (35786), *ilp2-Gal4* (37516), *Bcat-RNAi* (38363), *AkhR-RNAi* (51710), *Mhc-Gal4* (55133), *UAS-CaLexA* (66542), *pros*^*v1*^*-Gal4* (84276), *Lpp-Gal4* (84317), *UAS-hBcat2* (84835), and *UAS-GCaMP8f* (92588). *UAS-CD8-GFP* (108068) and *P{GawB}Myo31DF*^*NP0001*^ (112001) were obtained from the Kyoto *Drosophila* Genetic Resource Center (DGRC). *Bcat-RNAi* (1673R-2), *Atg6-RNAi* (5429R-3), *Atg1-RNAi* (10967R-1), *AkhR*^*SK1*^ (M2L-2825), and *AkhR*^*SK4*^ (M2L-2826) were obtained from the National Institute of Genetics (NIG) *Drosophila* Stock Center. *Bcat-RNAi* (38363) was used for ubiquitous knockdown, and *Bcat-RNAi* (1673R-2) was used for fat body- and muscle-specific knockdown. *Bcat*^*EY20842*^ was backcrossed four times to the *w*^*1118*^ control strain, and the resulting backcrossed line (*Bcat*^*EY*^) was used in all experiments.

Genotypes that contained temperature-sensitive *Tub-GAL80ts* were raised at 20 °C and maintained as adults for 3 days after eclosion, then shifted to 29 °C for 5–6 days before experiments. Adults were maintained under mixed-sex conditions, and males and females were collected from these vials (females under mated conditions). Unless otherwise indicated, experiments were performed using males; when females were used, this is specified in the corresponding figure panels or legends.

### Fly Food

Flies were reared on a standard diet (SD) containing 8 g agar, 100 g glucose, 40 g dry yeast, 40 g corn flour, 4 mL propionic acid, and 3 mL of 15% butylparaben (in 100% ethanol) per liter. Yeast paste was not used in any experiments. Flies were maintained under uncrowded conditions at 25°C. For transient starvation experiments, five- to seven-day-old adults were transferred to vials containing 0.8% agar and collected at the indicated time points. For rapamycin feeding, flies were placed in vials containing 0.8% agar supplemented with 100 µM rapamycin (R0097, Tokyo Chemical Industry) or an equivalent volume of DMSO for the indicated periods. For isotope tracing, starved flies were placed in vials containing either ^15^N-leucine (1 mM or 2 mM; NLM-142, Cambridge Isotope Laboratories, Inc.) or ^13^C_6_,^15^N-leucine (1 mM; HY-N0486S8, MedChemExpress) in 0.8% agar for the indicated period. High-protein diet (HPD) was prepared by supplementing SD with 10% peptone (#211677, Thermo Fisher Scientific). Amino acid-supplemented diets were prepared by adding amino acids to SD; ingredient details are provided in Supplementary Table 1.

### Starvation survival assay

For starvation assays, five- to seven-day-old adults were transferred to vials containing 0.8% agar, and the number of dead flies was recorded. For each condition, at least three independent cohorts of flies were analyzed, and all data were summed. Kaplan–Meier survival analysis was performed, and *P*-values were calculated using log-rank statistical analysis.

### qRT-PCR analysis

qRT-PCR was performed as previously described^71^. Total RNA was prepared using TRIzol (Invitrogen) and reverse transcription was performed using PrimeScript RT Master Mix (Takara Bio). qRT-PCR was conducted on an ABI PRISM 7500 (Thermo Fisher Scientific) using TB Green Premix Ex Taq II (Takara Bio). Target gene expression was normalized to *RpL32* mRNA levels. Primer sequences are listed in Supplementary Table 2.

### Akh measurement by LC-MS/MS

To quantify total Akh levels, two third-instar larvae (mixed sex) or two adult flies (male or female, as indicated) were collected and frozen in 1.5 mL plastic tubes per sample. For regional analysis, fifteen males and ten females were dissected by micro scissors, and heads/thoraxes and abdomens were collected separately. For hemolymph measurements, 40–50 flies were perforated with a tungsten needle and placed in a 0.5 mL tube perforated at the bottom with a 27 G needle. These tubes were placed inside 1.5 mL tubes and centrifuged at 5000 *g* for 5 min at 4 °C to collect hemolymph. A 5 µL aliquot of hemolymph was used per sample.

Frozen samples were homogenized in 300 µL cold methanol with a φ3 mm zirconia bead using a freeze crusher (TAITEC) at 33.3 Hz for 2 min. Homogenates were mixed with 200 µL methanol, 200 µL H_2_O, and 200 µL CHCl_3_, vortexed for 20 min at room temperature, and centrifuged at 15,000 rpm (20,000 *g*) for 15 min at 4 °C. The supernatant was mixed with 350 µL H_2_O, vortexed for 10 min, and centrifuged again. The aqueous phase was collected and dried down using a vacuum concentrator. Dried extracts were reconstituted in 15 µL of 10% methanol prior to LC-MS/MS. For extraction under acidic or basic conditions, the initial homogenization was performed in methanol containing 0.2% formic acid or 0.1% NH_4_OH.

Chromatography was performed on an ACQUITY BEH C18 column (50 ×2.1 mm, 1.7 µm particles, Waters) using an ACQUITY UPLC H-Class System (Waters). Mobile phases were 0.1% formic acid in acetonitrile (A) and 0.1% formic acid in H_2_O (B). A linear gradient was applied at 0.5 mL/min at 40 °C: 5% A (0–0.5 min), ramp to 50% A (0.5–2.5 min), hold at 50% A (2.5–3 min), and 5% A (3–5 min). Mass spectrometric analysis was performed using a Xevo TQD triple quadrupole mass spectrometer (Waters) with an electrospray ionization source in the positive ion mode. Peak areas were analyzed using MassLynx 4.1 software (Waters). Multiple reaction monitoring (MRM) transitions were: Akh (m/z 975.6 > 416.3 and 958.7); Akh-GK (m/z 581.5 > 120.1 and 602.5); Akh-GKR (m/z 659.6 > 120.1). Parameters were optimized using synthetic peptides (GenScript Japan). Quantification was performed using standard curves from serial dilution of each peptide.

### Metabolite measurement by LC-MS/MS

Metabolite measurement was performed as described previously^72,73^. Briefly, frozen samples in 1.5 mL tubes were homogenized in 300 µL cold methanol with a φ3 mm zirconia bead using a freeze crusher (TAITEC) at 33.3 Hz for 2 min. The homogenates were mixed with 200 µL methanol, 200 µL H_2_O, and 200 µL CHCl_3_, vortexed for 20 min at room temperature, and centrifuged at 15,000 rpm (20,000 *g*) for 15 min. The supernatant was mixed with 350 µL H_2_O, vortexed for 10 min, and centrifuged again. The aqueous and organic phases were separately dried using a vacuum concentrator. The aqueous extracts were reconstituted in 2 mM ammonium bicarbonate (pH 8.0) containing 5% methanol, while organic extracts were reconstituted in a mixture of acetonitrile and isopropanol (50:50). The insoluble pellets were washed with 70% ethanol, solubilized in 400 μl of 0.2 N NaOH, heat-denatured, and used for total protein quantification with a BCA Protein Assay Kit (Thermo Fisher Scientific). For cysteine and GSH/GSSG analysis, samples were homogenized in methanol containing 0.2% formic acid to prevent oxidation, then reconstituted in 0.1% formic acid containing 5% methanol prior to LC-MS/MS.

Chromatographic separation was performed using an ACQUITY UPLC H-Class System (Waters). ACQUITY UPLC HSS T3 column (100 × 2.1 mm, 1.8 µm particles, Waters) and ACQUITY UPLC BEH Amide column (100 × 2.1 mm, 1.7 µm particles, Waters) were used for aqueous metabolites, while an ACQUITY BEH C8 column (50 × 2.1 mm, 1.7 µm particles, Waters) was used for TAG and FFA analysis. Ionized compounds were detected using a Xevo TQD triple quadrupole mass spectrometer coupled with an electrospray ionization source (Waters). Peak area of each target metabolite was quantified using MassLynx 4.1 software (Waters). Metabolite signals were normalized to total protein level of the corresponding sample. Statistical significance was assessed using unpaired two-tailed Welch’s *t*-test in Microsoft Excel.

### Immunohistochemistry

Fly tissues were dissected in 1× PBS, fixed in 4% formaldehyde in PBS for 30–60 min at room temperature, and then washed three times in 0.1% PBST (PBS + 0.1% Triton X-100). Samples were blocked in blocking solution (0.1% PBST + 2% bovine serum albumin) for 1 h at room temperature, and then incubated with primary antibodies in the blocking solution at 4 °C overnight. After washing, samples were incubated with secondary antibodies for 2 h at room temperature in blocking solution. Primary antibodies included: rabbit anti-Akh antibody (1/500, gift from Dr. Jae H. Park), mouse anti-Akh antibody (1/500) ^74^, mouse anti-Elav (1/50, 9F8A9, DSHB), and chicken anti-GFP (1/4000, ab13970, Abcam). Secondary antibodies included: Alexa Fluor 488-conjugated goat anti-rabbit IgG antibody (1/500, A-11034, Thermo Fisher Scientific), Alexa Fluor 488-conjugated goat anti-chicken IgY (1/500, A-11039, Thermo Fisher Scientific), Alexa Fluor 555-conjugated goat anti-rabbit IgG antibody (1/500, A-21428, Thermo Fisher Scientific), and Alexa Fluor 555-conjugated goat anti-mouse IgG (1/500, A-21424, Thermo Fisher Scientific). Nuclei and F-actin were stained with DAPI (1/2000, D523, Dojindo Laboratories) and Alexa Fluor 488-Phalloidin (1/500, A12379, Thermo Fisher Scientific). Imaging was performed using an LSM800 confocal microscope, and images were processed and analyzed using Fiji software (version 2.16.0). CC cell size and number were quantified from confocal z-stacks spanning the entire CC cell cluster. Cell size was quantified as the cross-sectional area of cell bodies based on CD8-GFP signals, and cell number was counted as the number of His2A-mRFP+ cells.

For lipid droplet staining, abdominal carcasses were fixed and stained with LipidTOX (1:500 in 0.1% PBST, H34476, Thermo Fisher Scientific) for 2 h at room temperature.

### Ex vivo calcium imaging

Adult tissue samples were dissected in a modified adult hemolymph-like solution (AHL buffer; 103 mM NaCl, 3 mM KCl, 4 mM MgCl_2_, 1.5 mM CaCl_2_, 10 mM glucose, 10 mM trehalose, 1 mM NaH_2_PO_4_, 10 mM HEPES, pH7.2). This preparation consisted of the head capsule with the attached esophagus and CC (with surrounding tissue), together with the proventriculus and an anterior portion of the midgut. Dissected samples were embedded in a drop of 1% low-melting-temperature agarose (#5805A, Takara Bio) in AHL buffer on a 35 × 10 mm glass-bottom dish (#3910-035, IWAKI). Two fluorescence signals, calcium-dependent GCaMP8f and calcium-independent nuclear marker His2A-mRFP, were recorded with a 10× objective lens using a Zeiss LSM 800 confocal microscope. Samples in AHL buffer were first recorded for 5–6 min. The solutions were then changed to AHL buffer containing either 5 mM glycine, 5 mM threonine, a BCAA mixture (5 mM each), or 22 mM KCl (all pH-adjusted to 7.2–7.3). Fluorescence intensity in the CC cell cluster was quantified at each time point using a region of interest (ROI) in Fiji. To correct for axial drift and other fluctuations, a ratiometric trace was calculated as *R(t)* = *F*_G_*(t)*/*F*_R_*(t)*, where *F*_G_*(t)* is the GCaMP intensity and *F*_R_*(t)* is the His2A-mRFP intensity. Fluorescence changes were normalized as *ΔR/R*_0_ = [*R(t)* – *R*_0_]/*R*_0_, where *R*_0_ was defined as the mean of *R(t)* during the 0–0.5 min window immediately following medium change. For summary quantification, a single *ΔR/R*_0_ value was calculated for each sample as the mean *ΔR/R*_0_ during the 5–5.5 min window, and these per-sample values were pooled across animals for statistical analysis.

### Western blotting analysis

Abdominal carcasses from adult males were dissected in PBS and homogenized in 60 µL of 1× SDS sample buffer containing 1× Phosphatase Inhibitor Cocktail Solution I (#167-24381, FUJIFILM Wako) using a pellet pestle, then boiled for 10 min. The samples were centrifuged at 15,000 rpm (20,000 *g*) for 5 min at room temperature, and the supernatants were subjected to SDS–PAGE followed by western blotting using a PVDF membrane (Immobilon-P, Merck Millipore). Primary and secondary antibodies used were rabbit polyclonal anti-phospho-S6K (1:500, #9209, Cell Signaling Technology), rabbit monoclonal anti-phospho-Akt (1:500, #4060, Cell Signaling Technology), rabbit monoclonal anti-Akt (1:500, #4691, Cell Signaling Technology), goat polyclonal anti-GAPDH (1:500, IMG-3073, Imgenex), HRP-linked anti-rabbit IgG (1:2,000, #7074, Cell Signaling Technology), and HRP-linked anti-goat IgG (1:2,000, ab97110, Abcam). Proteins were visualized using Western BLoT Quant HRP Substrate (T7102, Takara) and detected with a LuminoGraph II imaging system (ATTO). Images were processed and analyzed using Fiji.

### CellROX staining

Abdominal carcasses were dissected in Schneider’s *Drosophila* Medium (#21720024, Thermo Fisher Scientific), transferred to 100 μl of Schneider’s Medium containing 1:100 CellROX Green reagent (C10444, Thermo Fisher Scientific), and incubated for 30 min at room temperature. Samples were then rinsed twice with 1x PBS, fixed in 4% formaldehyde in PBS for 30 min, and washed with 0.1% PBST. Tissues were incubated in 0.1% PBST containing Alexa Fluor 555-Phalloidin (1/500, A34055, Thermo Fisher Scientific) and DAPI (1/2000, D523, Dojindo Laboratories) for 30 min. After staining, samples were mounted on glass slides and imaged using a LSM800 confocal microscope (Zeiss). To quantify CellROX fluorescence, nuclear regions were automatically segmented based on DAPI signals, and the mean fluorescence intensity was analyzed using Fiji software.

### Ex vivo culture

Abdominal carcasses were dissected in Schneider’s *Drosophila* Medium (#21720024, Thermo Fisher Scientific) and incubated in 150 μl of Schneider’s Medium supplemented with 10% fetal bovine serum (#12483, Gibco), penicillin/streptomycin (#26253-84, Nacalai Tesque), and either 20 nM of synthetic mature Akh peptide or an equal volume of PBS (vehicle control). Samples were maintained at room temperature for 2 h on an orbital shaker, then collected for RNA extraction.

### Protein carbonyl assay

Protein oxidative damage was assessed by measuring protein carbonyl content. For each condition, three male flies were collected in 1.5 mL tubes and stored at –80°C until use. Frozen samples were homogenized in 300 µL cold methanol with a φ3 mm zirconia bead using a freeze crusher (TAITEC) at 33.3 Hz for 2 min. Homogenates were mixed with 700 µL methanol, vortexed for 10 min at room temperature, and centrifuged at 15,000 rpm (20,000 *g*) for 10 min at room temperature. The supernatant was discarded, and the pellet was washed with 1 mL of 70% ethanol, resuspended in 100 µL of 50 mM Tris/HCl (pH 8.0), and treated with 10 µL of 10% streptozotocin (#197-15153, FUJIFILM Wako) for 30 min at 37°C. After adding 500 µL of 2 N HCl, samples were boiled at 95°C for 10 min, then treated with 100 µL of 0.3% 2,4-dinitrophenylhydrazine hydrochloride (DNPH) in HCl (A5531, Tokyo Chemical Industry). After 30 min at room temperature, 600 µL of 33% trichloroacetic acid (TCA) was added to precipitate proteins. The precipitates were collected by centrifugation at 15,000 rpm (20,000 *g*) for 10 min at 4°C, and pellets were washed twice with 1 mL of cold acetone to remove free DNPH. The resulting protein pellets were dissolved in 200 µL of 8 M urea in 50 mM Tris/HCl (pH 8.0) and heat-denatured at 95°C for 10 min. After centrifugation, 150 µL of the supernatant was transferred to a 96-well plate, and absorbance at 370 nm was measured using a Varioskan LUX microplate reader (Thermo Fisher Scientific). Protein amounts were subsequently determined using a BCA protein assay kit (Thermo Fisher Scientific), and carbonyl content was normalized to total protein levels for each sample.

### Lipid peroxidation (MDA) assay

Lipid oxidative stress was determined by measuring malondialdehyde (MDA) levels using a commercial kit (ab118970, Abcam). In brief, ten male flies per group were collected into 1.5 mL tubes and stored at –80°C until use. Frozen samples were homogenized in 150 µL of H_2_O containing 3 µL of butylated hydroxytoluene (BHT) using a φ3 mm zirconia bead in a freeze crusher (TAITEC) at 33.3 Hz for 2 min. Homogenates were mixed with 150 µL of 2 N perchloric acid, vortexed for 10 min at room temperature, and centrifuged at 15,000 rpm (20,000 *g*) for 10 min at room temperature. The supernatant was used to generate thiobarbituric acid (TBA) adducts according to the manufacturer’s protocol. Fluorescence was measured at Ex/Em = 532/553 nm using a Varioskan LUX multimode microplate reader (Thermo Fisher Scientific). The remaining pellets were washed with 500 µL of 70% ethanol, re-dissolved in 500 µL of 0.2 N NaOH, heat-denatured, and used for total protein quantification with a BCA Protein Assay Kit (Thermo Fisher Scientific). MDA levels were normalized to total protein content in each sample.

### Statistics & Reproducibility

Experiments were independently repeated at least twice using separately reared populations, with similar results. Unless otherwise stated, data were pooled from independent repeats. The definition of n (e.g., cells, individual flies, or biological batches) is provided in the figure legends. No statistical method was used to predetermine sample size. Sample sizes were determined based on technical considerations, prior experience, and consistency with previous studies, and are indicated in the figure panels or legends. No data were excluded from the analyses. The experiments were not randomized, and the investigators were not blinded to allocation during experiments and outcome assessment.

Statistical analyses were performed using Microsoft Excel or GraphPad Prism 7. Normality of data distribution and homogeneity of variance were not formally tested. For comparisons between two groups, we used a two-tailed Student’s t-test (when variance was assumed to be comparable), a two-tailed Welch’s t-test (when variances were expected to differ, e.g., amino acid measurements), or a two-tailed Mann–Whitney U-test (for nonparametric comparisons). For comparisons among three or more groups, we performed one-way ANOVA followed by Dunnett’s (compared each group with the control) or Tukey’s (all pairwise comparisons) post hoc test, or the Kruskal–Wallis test followed by Dunn’s multiple comparisons test (when distributional assumptions were unclear). For survival assays, Kaplan–Meier survival analysis was performed, and *P* values were calculated using the log-rank test. Data were considered statistically significant at *P * <  0.05. Where applicable, *P* values were adjusted for multiple comparisons using the Bonferroni correction. Specific statistical tests are described in the figure legends.

### Reporting summary

Further information on research design is available in the Nature Portfolio Reporting Summary linked to this article.

## Supplementary information

Supplementary Information

Reporting Summary

Transparent Peer Review file

## Source data

Source Data

Source Data (uncropped blots)

## Data Availability

The source data underlying all main and Supplementary Figs. are provided as a Source Data file. Sequences of oligonucleotides used in this study are included in Supplementary Table 2. This study used targeted LC–MS/MS (MRM-based) analysis for metabolite quantification. The quantitative data underlying these analyses are provided in the Source Data file. Source data are provided with this paper.
