# Supplementary material for: Inter-organ metabolic feedback via BCAA catabolism regulates glucagon-like hormone secretion in Drosophila
Source: Nat Commun. 2026 May 9;17:6278. doi: 10.1038/s41467-026-72677-1 (PMC13376891; doi:10.1038/s41467-026-72677-1)
Supplement: Supplementary file 1 — Supplementary Information [file 41467_2026_72677_MOESM1_ESM.pdf]

Supplementary Information

Inter-organ metabolic feedback via BCAA catabolism regulates glucagon-like hormone secretion in *Drosophila*

Takashi Nishimura[1,2*], Chisei Arakawa[1,3,4], and Yuto Yoshinari[1,2,4]

[1]Laboratory of Metabolic Regulation and Genetics, Institute for Molecular and Cellular Regulation, Gunma University, Maebashi, Gunma 371-8512, Japan
[2]Laboratory of Metabolic Regulation and Genetics, Gunma University Graduate School of Medicine, Maebashi, Gunma 371-8512, Japan
[3]School of Medicine, Faculty of Medicine, Gunma University, Maebashi, Gunma 371-8512, Japan

[4]These authors contributed equally
[*]Correspondence: t-nishimura@gunma-u.ac.jp

Supplementary Fig. 1–11
Supplementary Tables 1–2

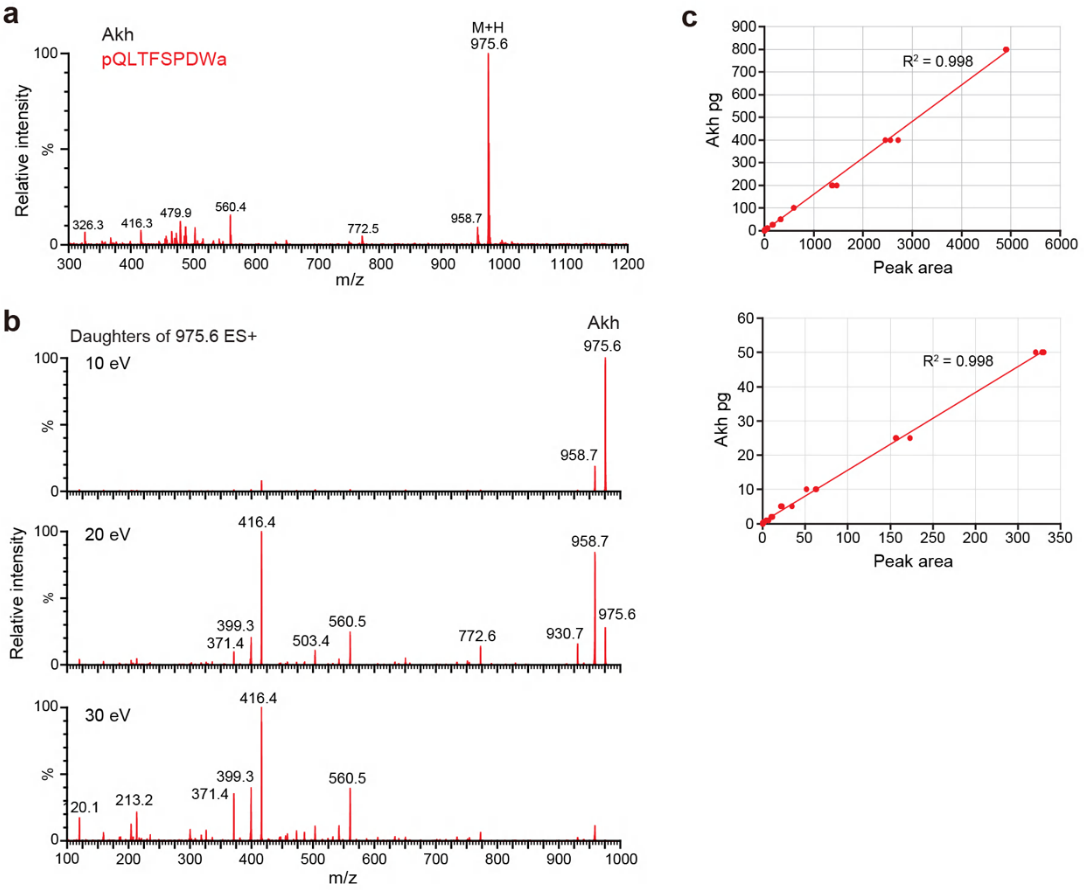

Supplementary Fig. 1: LC-MS/MS detection of Akh, related to Fig. 1.

a MS1 spectrum of the synthetic Akh peptide. **b** MS2 spectra of Akh acquired under three different collision energies (10, 20, 30 eV). **c** Calibration curve for Akh generated using method of multiple reaction monitoring (MRM). The bottom panel shows a magnified view of the low concentration range.

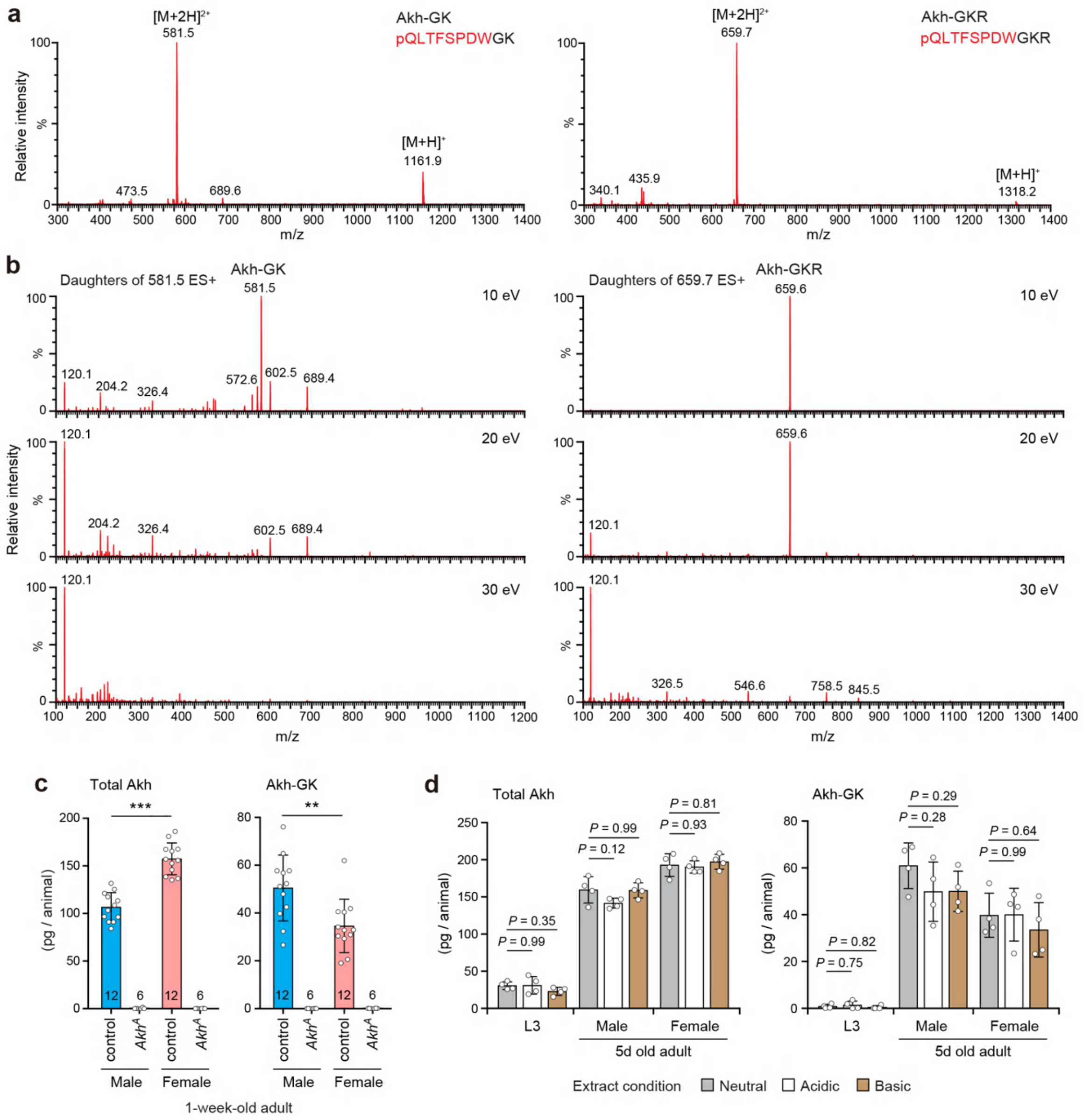

Supplementary Fig. 2: Detection of Akh and its precursors by LC-MS/MS, related to Fig. 1.

a MS1 spectra of synthetic Akh-GK and Akh-GKR peptides. Insets show the amino acid sequences of each precursor. Akh-GK and Akh-GKR were mainly detected as doubly charged ions at m/z 581.5 and 659.7, respectively, with a smaller proportion of singly charged ions at m/z 1161.9 and 1318.2. **b** MS2 spectra of Akh-GK and Akh-GKR obtained at collision energies of 10, 20, and 30 eV. **c** Quantification of total Akh and Akh-GK in control and *Akh* mutant males and females under fed conditions. **d** Levels of total Akh and Akh-GK in control animals extracted under neutral, acidic (0.2% formic acid), or basic (0.1% NH_4OH) conditions. Results are presented as mean ±

SD (**c**, **d**); n = 4 (**d**) batches. Values of n indicate the number of batches (**c**). Statistical tests: unpaired two-tailed Student's *t*-test (**c**), one-way ANOVA with Dunnett's post hoc test (**d**); **p < 0.01; ***p < 0.001. Source data are provided as a Source Data file, which also includes exact P values.

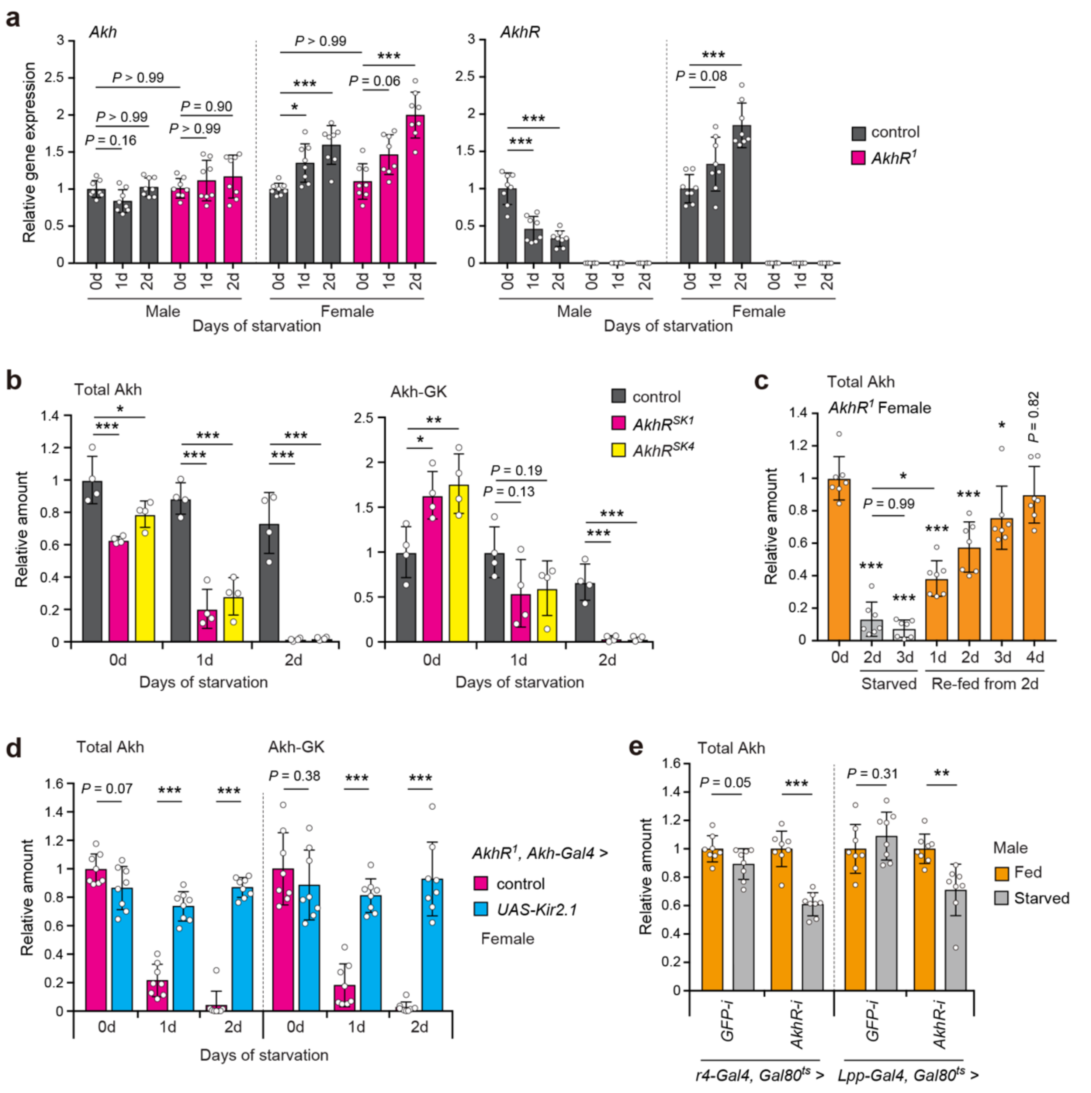

Supplementary Fig. 3: *AkhR* mutants deplete Akh during starvation, related to Fig. 2.

a Relative gene expression levels of *Akh* and *AkhR* in control or *AkhR* mutants during starvation. Adult male flies maintained on a standard diet (0d) were transferred to agar-only vials and collected at the indicated days. 1d, 1 day starved; 2d, 2 days starved. **b** Relative changes in total Akh and Akh-GK levels during starvation. **c** Relative amounts

of total Akh in *AkhR* mutant females after re-feeding. Two days starved flies (2d) were re-fed on a standard diet. **d** Relative changes in total Akh and Akh-GK levels in *AkhR* mutant females during starvation, in which secretion was inhibited by expressing Kir2.1 in CC cells. **e** Relative changes in total Akh levels in adult-stage, fat body-specific knockdown of *AkhR* during starvation. Flies were raised at 20 °C and maintained as adults for 3 days after eclosion, then shifted to 29 °C for 5–6 days before experiments. Results are presented as mean ± SD; n = 8 (**a**, **d**, **e**), 4 (**b**), or 7 (**c**) batches. Statistical tests: unpaired two-tailed Student's *t*-test with Bonferroni correction (**a**), one-way ANOVA with Dunnett's post hoc test (**b**), one-way ANOVA with Tukey's post hoc test (**c**), unpaired two-tailed Student's *t*-test (**d**, **e**); *p < 0.05; **p < 0.01; ***p < 0.001. Source data are provided as a Source Data file, which also includes exact P values.

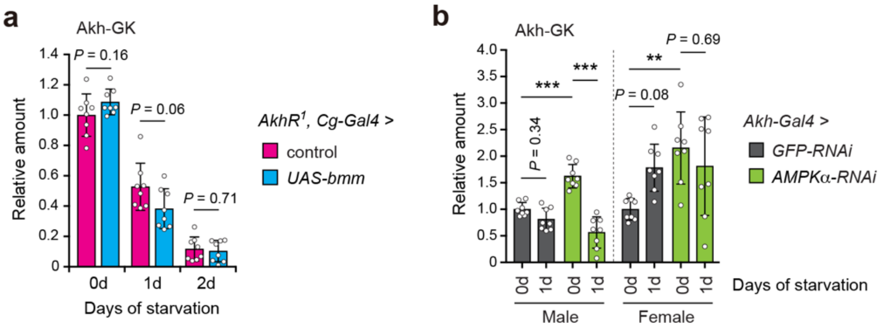

Supplementary Fig. 4: Metabolic regulation of Akh secretion, related to Fig. 3.
a Relative amounts of Akh-GK in *AkhR* mutants during starvation with overexpression of *bmm* in the fat body. 0d, fed; 1d, 1 day starved; 2d, 2 days starved. **b** Relative amounts of Akh-GK in flies with *AMPKα* knockdown in CC cells. Results are presented as mean ± SD; n = 8 (**a**, **b**) batches. Statistical tests: unpaired two-tailed Student's *t*-test (**a**), one-way ANOVA with Tukey's post hoc test (**b**); *p < 0.05; **p < 0.01; ***p < 0.001. Source data are provided as a Source Data file, which also includes exact P values.

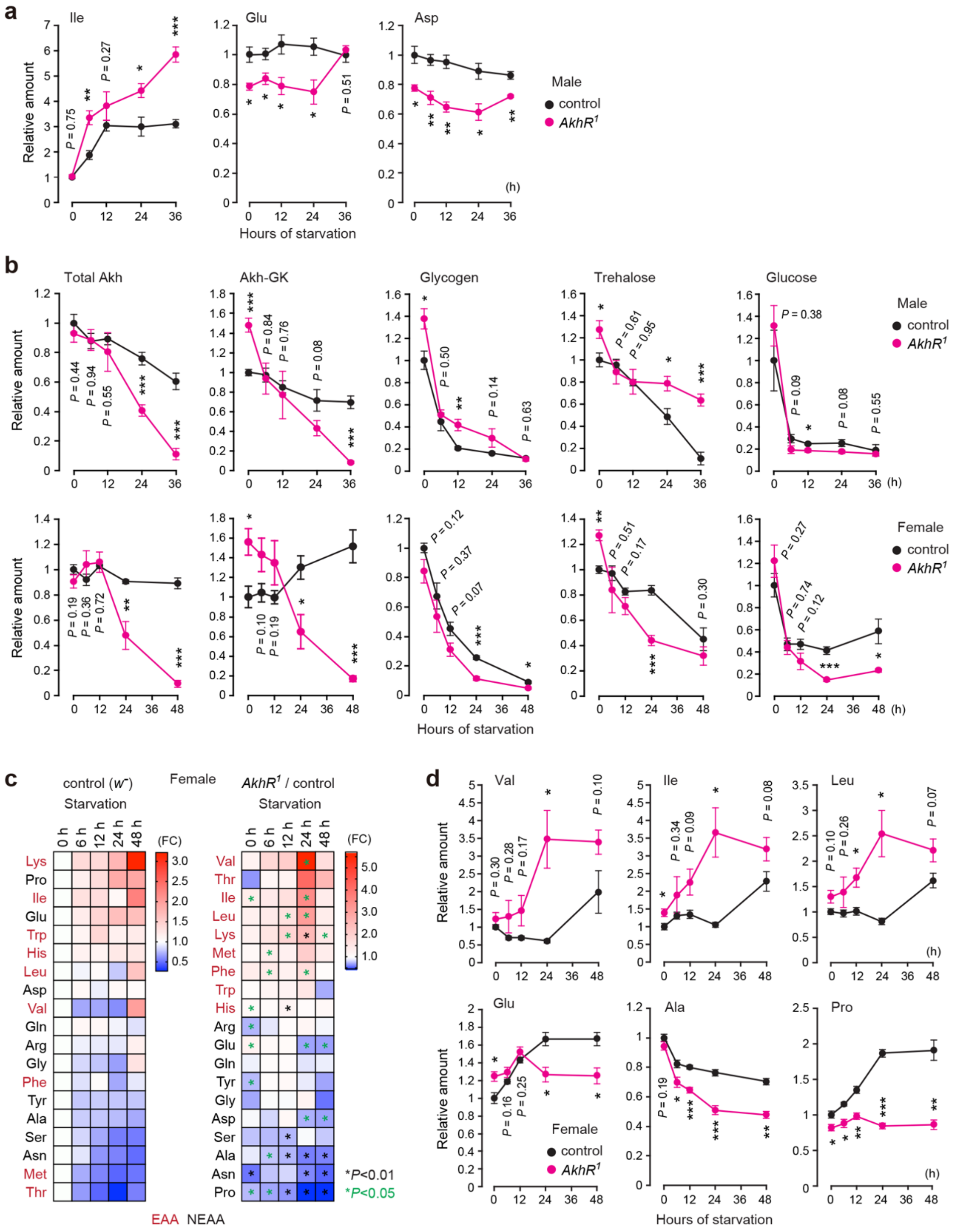

Supplementary Fig. 5: Total Akh and metabolite levels in *AkhR* mutants during starvation, related to Fig. 4.

a Relative amounts of selected amino acids in control and *AkhR* mutant males during starvation. Fold changes are shown relative to the average value in control flies at 0

hours (h), set to 1. **b** Relative amounts of total Akh, Akh-GK, and metabolites in control and *AkhR* mutants during starvation. **c** Heatmaps of amino-acid fold changes during starvation in female flies. Control: fold change relative to 0 h in control. *AkhR* mutants: fold change relative to time-matched control. Red and blue indicate increased or decreased metabolites, respectively. FC, fold change; EAA, essential amino acids; NEAA, non-essential amino acids. **d** Relative amounts of selected amino acids in control and *AkhR* mutant females during starvation. Results are presented as mean ± SEM (**a**, **b, d**); n = 4 (**a**–**d**) batches. Statistical tests: unpaired two-tailed Welch's *t*-test (**a**, **c**, **d**), unpaired two-tailed Student's *t*-test (**b**); *p < 0.05; **p < 0.01; ***p < 0.001 (except in **c**). Source data are provided as a Source Data file, which also includes exact P values.

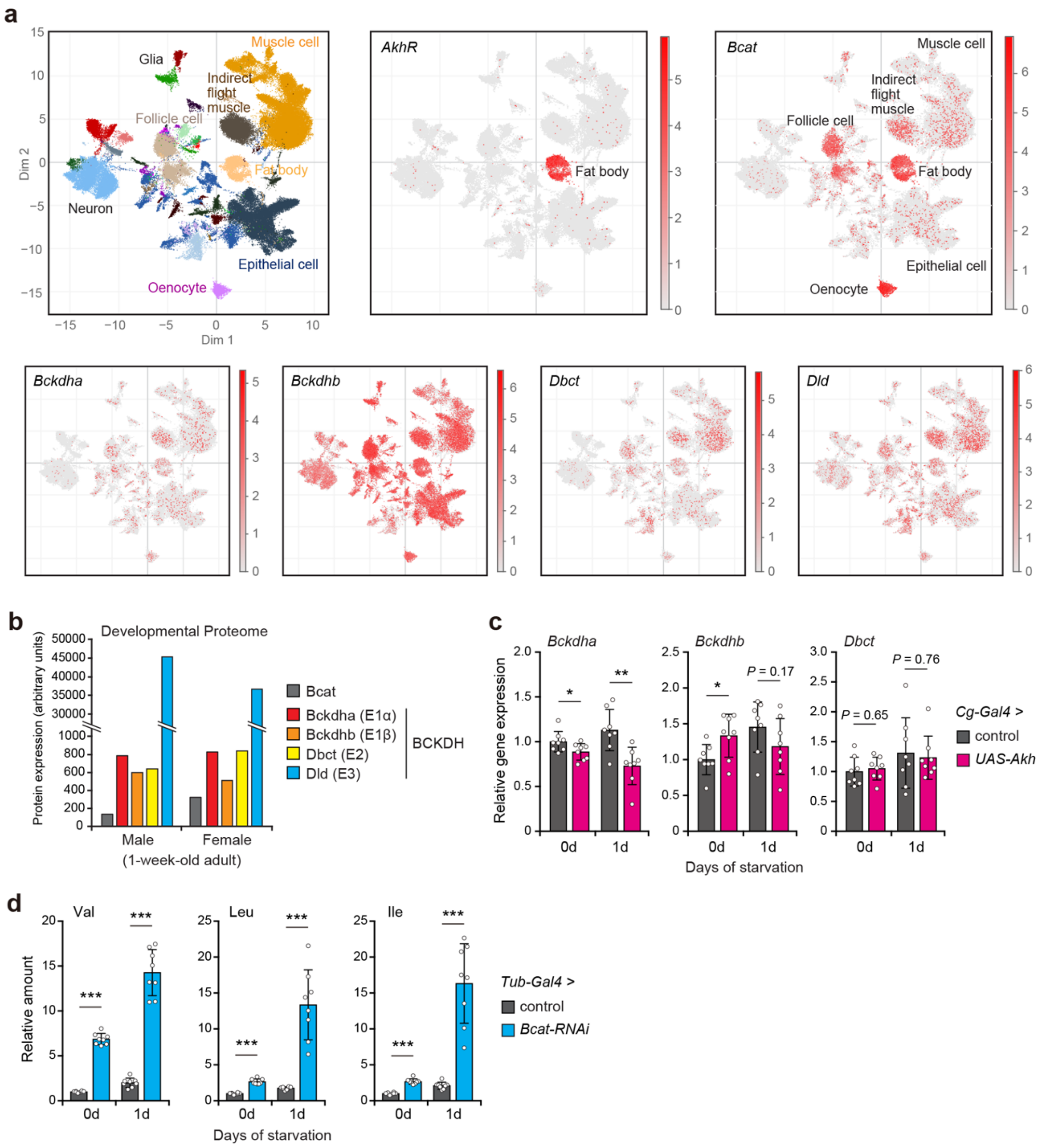

Supplementary Fig. 6: mRNA and protein expression of BCAA catabolic enzymes, related to Fig. 4.

a UMAP plots from the Fly Cell Atlas scRNA-seq data (stringent 10x dataset from the fly body) showing expression of *AkhR*, *Bcat*, and components of the branched-chain α-keto acid dehydrogenase (BCKDH) complex. Each color indicates a distinct cell cluster. Bckdha and Bckdhb encode the E1α and E1β subunits, respectively; Dbct encodes the E2 (dihydrolipoamide branched-chain transacylase) subunit; and Dld encodes E3 (dihydrolipoamide dehydrogenase) subunit. **b** Protein expression levels of Bcat and BCKDH components in one-week-old adult males and females from the Developmental

Proteome Database. Note that the E1 and E2 subunits are specific to the BCKDH complex, whereas the E3 subunit Dld is shared among multiple dehydrogenase complexes (e.g., pyruvate dehydrogenase) and thereby shows high overall abundance. **c** Relative mRNA levels of BCKDH components in male flies overexpressing *Akh* under fed (0d) or 1-day starvation (1d) conditions. **d** Relative BCAA levels in flies with ubiquitous *Bcat* knockdown using *Tub-Gal4* during starvation. Results are presented as mean ± SD; n = 8 (**c**, **d**) batches. Statistical tests: unpaired two-tailed Student's *t*-test (**c**), unpaired two-tailed Welch's *t*-test (**d**); *p < 0.05; **p < 0.01; ***p < 0.001. Source data are provided as a Source Data file, which also includes exact P values.

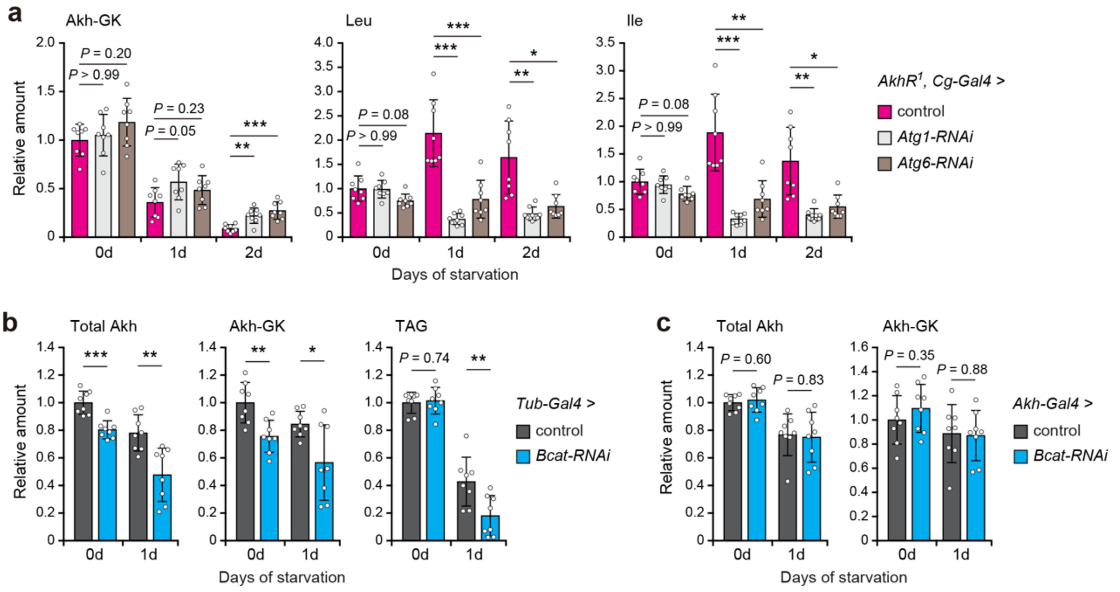

Supplementary Fig. 7: BCAA catabolism in the fat body remotely suppresses Akh secretion, related to Fig. 6.

a Relative amounts of Akh-GK and BCAAs in *AkhR* mutants during starvation with fat body-specific autophagy inhibition. 0d, fed; 1d, 1 day starved; 2d, 2 days starved. **b** Relative amounts of total Akh, Akh-GK, and TAG in flies with ubiquitous *Bcat* knockdown during starvation. **c** Relative amounts of total Akh and Akh-GK in flies with *Bcat* knockdown in CC cells during starvation. Results are presented as mean ± SD; n = 8 (**a**–**c**) batches. Statistical tests: unpaired two-tailed Welch's *t*-test with Bonferroni correction (**a**), unpaired two-tailed Student's *t*-test (**b**, **c**); *p < 0.05; **p < 0.01; ***p < 0.001. Source data are provided as a Source Data file, which also includes exact P values.

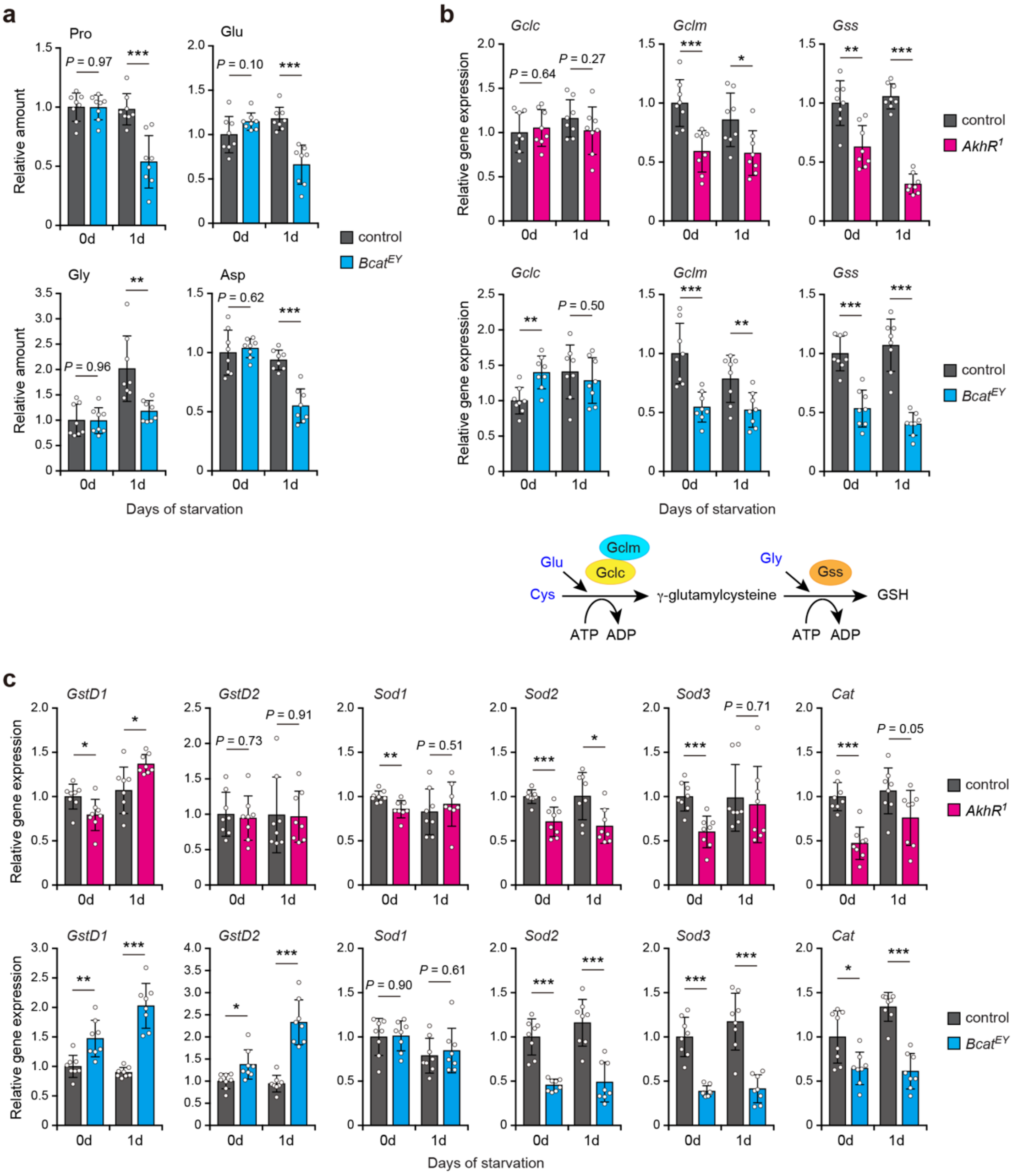

Supplementary Fig. 8: BCAA catabolism supports glutathione synthesis and oxidative-stress responses, related to Fig. 7.

a Relative amounts of selected amino acids in control and *Bcat* mutants under fed (0d) and 1-day starvation (1d) conditions. **b** Relative mRNA expression of glutathione biosynthetic enzymes during starvation. Gclc, glutamate–cysteine ligase catalytic subunit; Gclm, modifier subunit; Gss, glutathione synthetase. Schematic of glutathione synthesis highlighting these enzymes. **c** Relative mRNA expression of oxidative stress–response genes during starvation, including GstD1/GstD2 (glutathione S-transferases),

Sod1–Sod3 (superoxide dismutases), and Cat (catalase). Results are presented as mean ± SD; n = 8 (**a**–**c**) batches. Statistical tests: unpaired two-tailed Welch's *t*-test (**a**), unpaired two-tailed Student’s *t*-test (**b, c**); *p < 0.05; **p < 0.01; ***p < 0.001. Source data are provided as a Source Data file, which also includes exact P values.

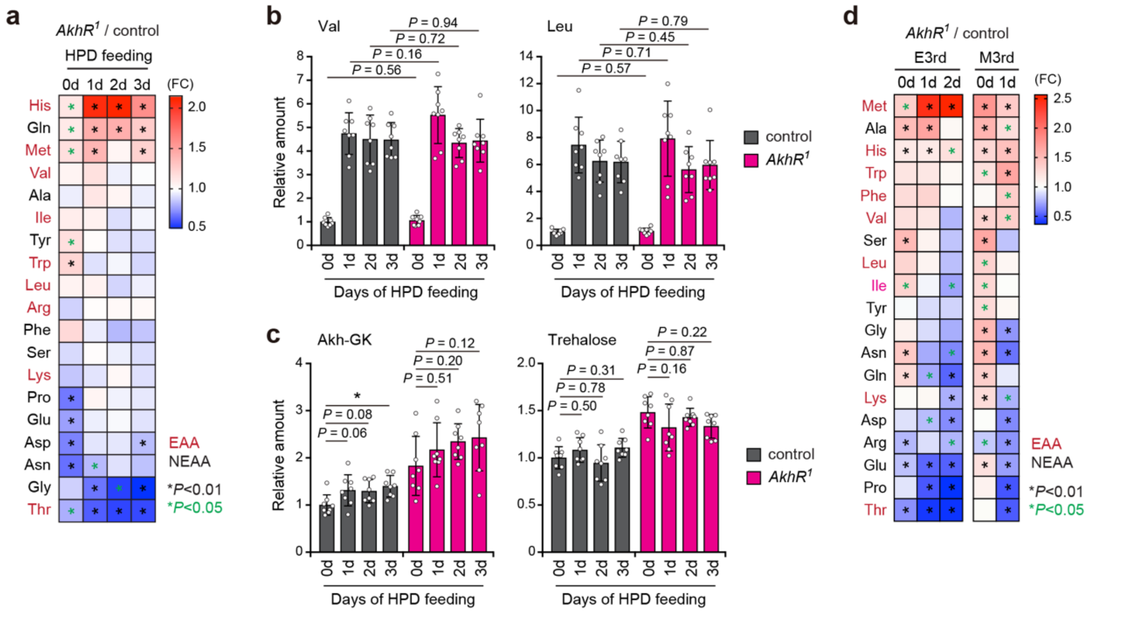

Supplementary Fig. 9: Response to high protein diet in *AkhR* mutants, related to Fig. 8.

a Heatmap of amino acid levels in *AkhR* mutants after feeding a high protein diet (HPD). Red and blue indicate increased and decreased metabolite levels, respectively, relative to control values at each time point. 1d, 1 day; 2d, 2 days; 3d, 3 days; FC, fold change; EAAs, essential amino acids; NEAAs, non-essential amino acids. **b** Relative amounts of valine and leucine in control and *AkhR* mutants after HPD feeding. **c** Relative amounts of Akh-GK and trehalose in control and *AkhR* mutants after HPD feeding. **d** Heatmaps of amino acid levels in *AkhR* mutant larvae during starvation. Red and blue indicate increased and decreased metabolite levels, respectively, relative to control values at each time point. Results are presented as mean ± SD (**b**, **c**); n = 8 (**a**–**c**) or 11 (**d**) batches. Statistical tests: unpaired two-tailed Welch's *t*-test (**a**, **b**, **d**), one-way ANOVA with Dunnett’s post hoc test (**c**); *p < 0.05 (except in **a** and **d**). Source data are provided as a Source Data file, which also includes exact P values.

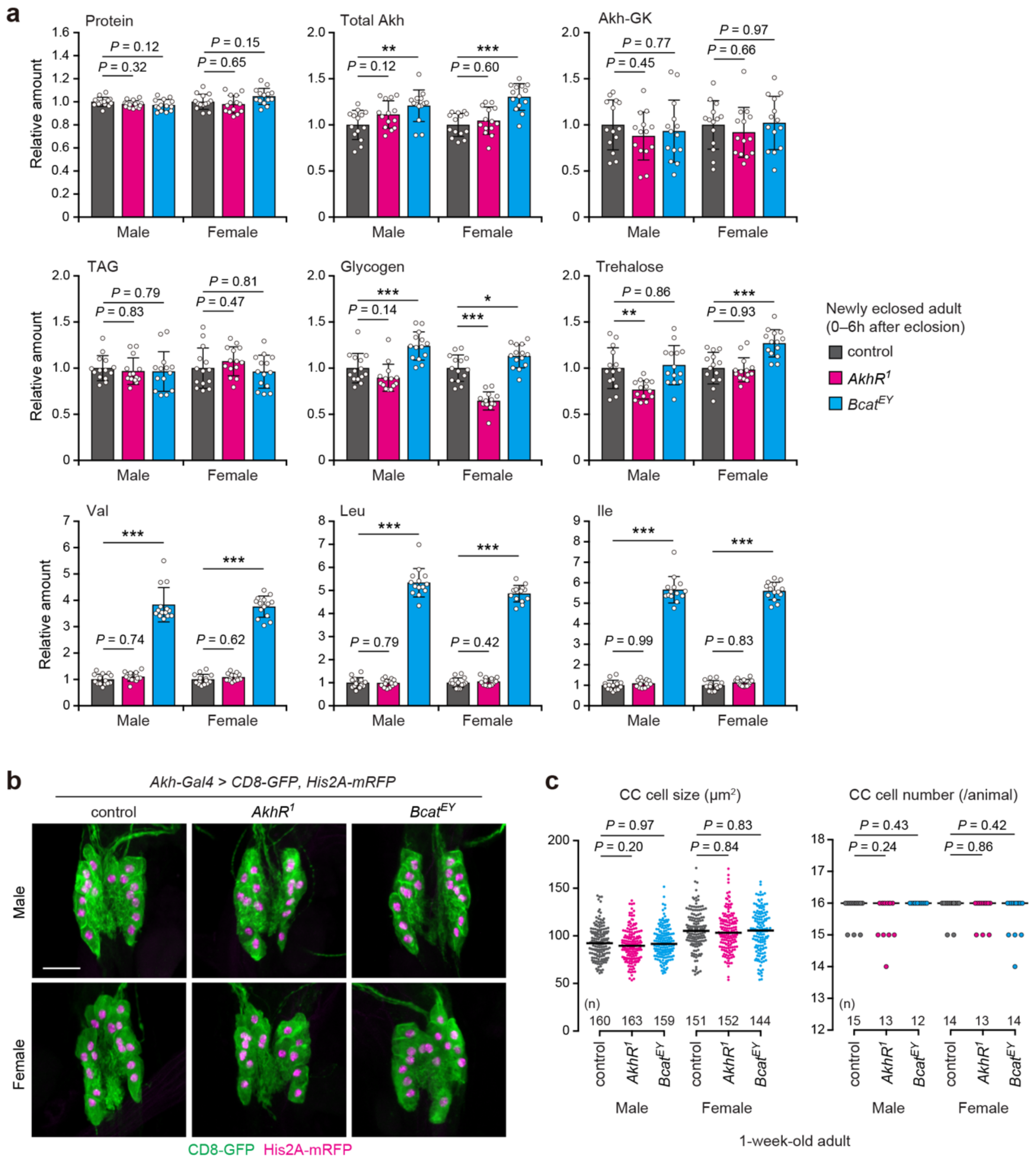

Supplementary Fig. 10: Akh and metabolite levels in newly eclosed adult and CC cell quantification, related to Fig. 8.

a Akh and selected metabolite levels in newly eclosed *AkhR* and *Bcat* mutants (0–6 h after eclosion). **b** Representative images of CC cell cluster in *AkhR* and *Bcat* mutants expressing *Akh-Gal4* driven CD8-GFP and His2A-mRFP. One-week-old males and females are shown. Scale bar: 20 μm. **c**, Quantification of CC cell size and number. Cell size was quantified as the cross-sectional area of cell bodies based on CD8-GFP signals, and cell number was counted as the number of His2A-mRFP+ cells. Horizontal lines represent group medians. Results are presented as mean ± SD (**a**); n = 14 (**a**) batches.

Statistical tests: one-way ANOVA with Dunnett's post hoc test (**a**, **c**); *p < 0.05; **p < 0.01; ***p < 0.001. Source data are provided as a Source Data file, which also includes exact P values.

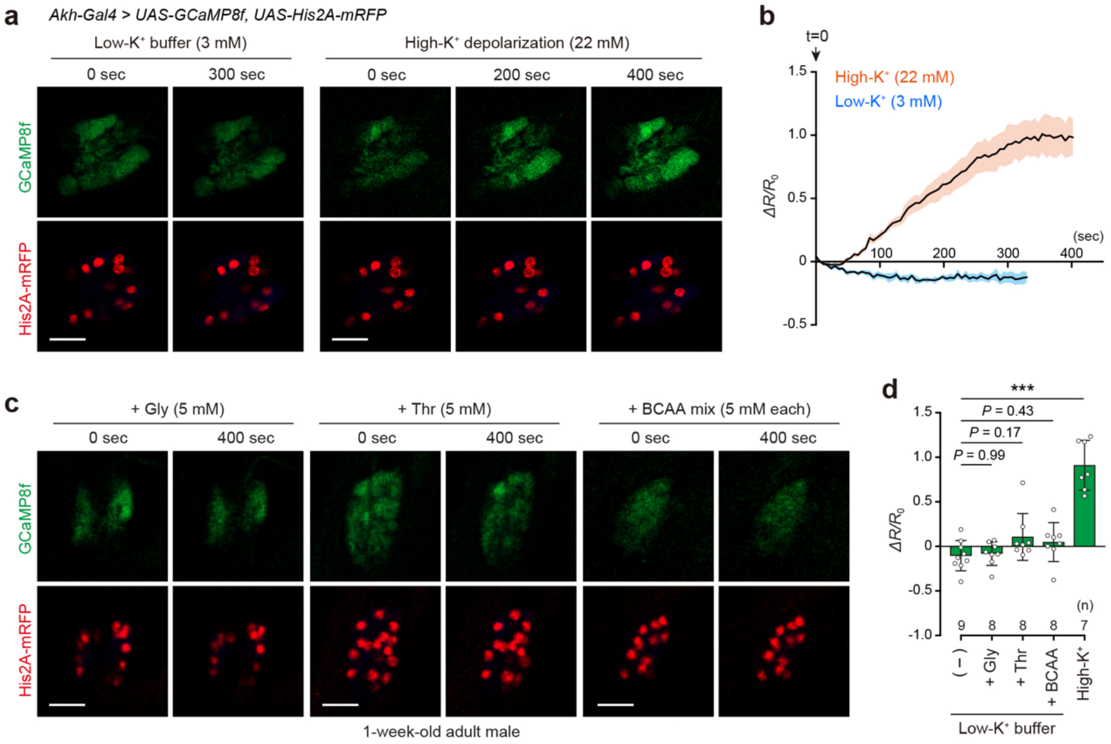

Supplementary Fig. 11: Ex vivo calcium imaging of adult CC cells, related to Fig. 8.

a Representative Ca^{2+} activity in CC cell clusters. Adult tissue samples containing CC cells, which express GCaMP8f and His2A-mRFP, were dissected from one-week-old adult males and maintained in adult hemolymph-like solution (AHL) containing low-K^+ (3 mM) or high-K^+ (22 mM). **b** Time-course data of Ca^{2+} activity. Fluorescence changes are shown as $\Delta R/R_0$, where $R(t) = F_G(t)/F_R(t)$ (GCaMP8f fluorescence normalized to the His2A-mRFP signal) and R_0 is the mean $R(t)$ during the 0–0.5 min window immediately following solution exchange. Results are presented as mean ± SEM (shaded region; n = 4 animals). **c** Representative Ca^{2+} activity in CC cell clusters in AHL containing the indicated amino acids. **d** Quantification of Ca^{2+} activity in CC cell clusters 5 min after addition of the indicated amino acids or high-K^+ solution. Results are presented as mean ± SD; n indicates the number of animals. Statistical tests: one-way ANOVA with

Dunnett's post hoc test; ***p < 0.001. Scale bars: 20 µm. Source data are provided as a Source Data file, which also includes exact P values.

Standard diet +

		g/L	mM		
BCAA diet	Valine	27.5	234.7	Wako	#228-00082
	Leucine	7.5	57.2	Wako	#124-00852
	Isoleucine	15	114.4	Wako	#121-00862
	SUM	50			

		g/L	mM		
EAA diet	Phenylalanine	13	78.7	Wako	#161-01302
	Histidine	10	64.5	Wako	#084-00682
	Lysine	19	130	Wako	#121-01462
	Methionine	8	53.6	Wako	#133-01602
	Arginine	8	45.9	Wako	#017-04612
	Threonine	20	167.9	Wako	#204-01322
	Valine	28	239	Wako	#228-00082
	Tryptophan	5	24.5	Wako	#204-03382
	SUM	111			

		g/L	mM		
NEAA diet	Alanine	17.5	196.4	Wako	#010-01042
	Cysteine	0.5	4.1	Wako	#033-05272
	Aspartic acid	8.5	63.9	Wako	#013-04832
	Glycine	16	213.1	Wako	#073-00732
	Asparagine	8.5	64.3	Wako	#019-04812
	Proline	7.5	65.1	Wako	#161-04602
	Glutamine	12.5	85.5	Wako	#074-00522
	Serine	9.5	90.4	Wako	#199-00402
	SUM	80.5			

Supplementary Table 1.

Composition of amino-acid-supplemented diet.

RpL32 sense	CAGTCGGATCGATATGCTAAGCTG
RpL32 antisense	TAACCGATGTTGGGCATCAGATAC
Akh sense	CGTCCAGTGTCAATTGACCTTCTC
Akh antisense	AGCAGCATTTCGTTGGAGGTCTTG
AkhR sense	TTCAACTCGTTCAGGAGCGACTTTG
AkhR antisense	AGATGAACATGATCAGCGGAAAGGC
bmm sense	CTTCAGGGTGGTGAACGAAGCTCG
bmm antisense	TGCCGGAGAATCCGGGTATGAAGC
Bcat sense	ACCACAAGAGTTTGGGCGGATGGC
Bcat antisense	TGGGATCAATGCCGATCAGTGTGG
Bckdha sense	CAATTTACCGGGTGATGGACCAGG
Bckdha antisense	ATGAGATCCCGCATCTCCAAGGCG
Bckdhb sense	GTTGGGGTACACAAGTCCATGTGC
Bckdhb antisense	AGGCTCGAAGACATGGGGAAACGG
Dbct sense	TCAAGGGAGTGCGCAAGGCCATGC
Dbct antisense	TGTGGGCACCCTTGAAGACCAGCG
Gclc sense	TGAACAGAAGGTGGCACGTGTGGC
Gclc antisense	ATAACGCACTCGTCCTTGGCCAGC
Gclm sense	GATTAGCACGGGCAACATCATCGC
Gclm antisense	GATTCACAGACTCTGTGGAGTGGC
Gss1/2 sense	GACGCCAATACGGCCGTGCTGCGC
Gss1/2 antisense	GTGCATCAGCCGGTTGATGATCGG
GstD1 sense	CGCCTGTACTTCGACATGGGAACG
GstD1 antisense	TCGTACCACCTGTTCACATTGGCG
GstD2 sense	TCCGCACTGGAAAGCCCGGATCGG
GstD2 antisense	GGCATCGAACAACGCCTTCATCGC
Cat sense	TGGAGCAGATCGCCTTCAGTCCCG
Cat antisense	GGCATTCCTGGGGACCGTTGAACG
Sod1 sense	TTAACGGCGATGCCAAGGGCACGG
Sod1 antisense	TGGCCTCAATGTTGCCCAGATCGC
Sod2 sense	GCCATCGAGTCGCAGTGGAAGAGC
Sod2 antisense	TGGAAGCGGCACGAGATGTCATCC
Sod3 sense	GTTGTTAGCCTGGCACTCTGTGCC
Sod3 antisense	TGAATGTGGAAGCCGTGCTTGCCC

Supplementary Table 2.

List of oligonucleotides used for qRT-PCR analysis.
