# Supplementary figures and images for: Inter-organ metabolic feedback via BCAA catabolism regulates glucagon-like hormone secretion in Drosophila
Source: Nat Commun. 2026 May 9;17:6278. doi: 10.1038/s41467-026-72677-1 (PMC13376891; doi:10.1038/s41467-026-72677-1)

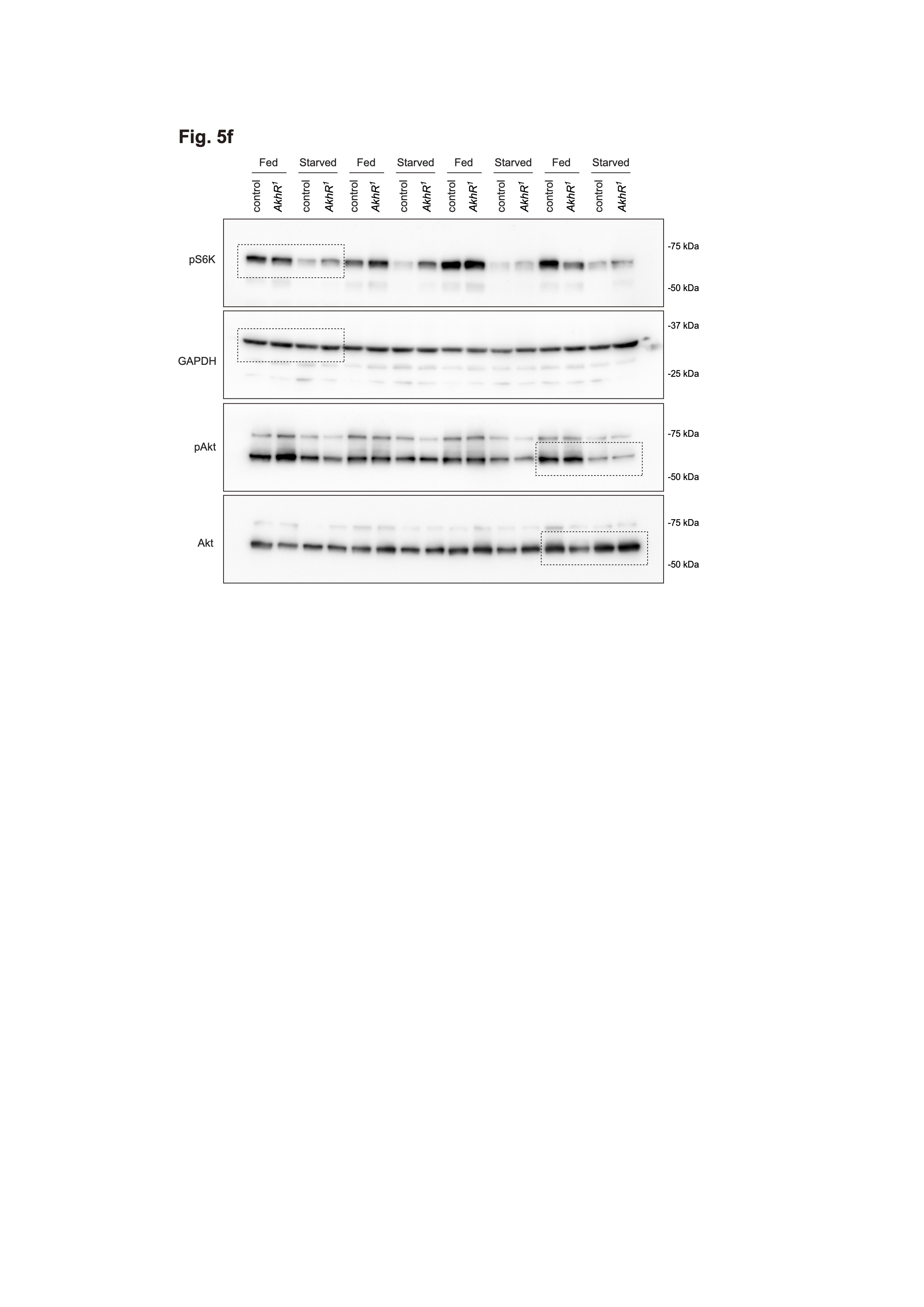

Supplement: Supplementary file 5 — Source Data (uncropped blots) [file 41467_2026_72677_MOESM5_ESM.jpg]
