# Supplementary material for: Inter-organ metabolic feedback via BCAA catabolism regulates glucagon-like hormone secretion in Drosophila
Source: Nat Commun. 2026 May 9;17:6278. doi: 10.1038/s41467-026-72677-1 (PMC13376891; doi:10.1038/s41467-026-72677-1)
Supplement: Supplementary file 3 — Transparent Peer Review file [file 41467_2026_72677_MOESM3_ESM.pdf]

nature portfolio

Peer Review File

Inter-organ metabolic feedback via BCAA catabolism regulates glucagon-like hormone secretion in Drosophila

Corresponding Author: Professor Takashi Nishimura

This file contains all reviewer reports in order by version, followed by all author rebuttals in order by version.

Version 0:

Reviewer comments:

Reviewer #1

(Remarks to the Author)
This is a really interesting article; overall, the authors identify that BCAA catabolism is controlled by Akh, a glucagon like peptide, by inducing the expression of the first step of BCAA catabolism, with downstream effects on BCAA levels and glutathione and oxidative stress. However, there are strong gaps in the mechanisms explored, and I find the overall writing very confusing - I think cause and effect are not well distinguished in many of the proposed mechansisms. Some of the things I was thinking about that suggest to me that revision is required:

1. My main critique is that the proposed mechanism of action of Akh makes no sense biochemically. Bcat is the first step of BCAA catabolism, but Bcat is freely reversible and is not the rate limiting step; that is the next step in BCAA catabolism, regulated by BCKDH. As such, the roughly 2x difference in Bcat gene expression resulting from Akh loss should have no biologically significant effect. IF Akh is working as proposed, it must also be regulating the fly homologues of Bckdh or Bckdk; and the authors should examine the gene and protein expression of the entire BCAA catabolic pathway to be comprehensive. Flux could also be examined with stable isotopes to give a thorough assessment.

2. I was surprised that mTOR was not discussed, as the massive increase in BCAA levels should be more than sufficient to effect mTOR via Sestrin binding, and 2-3 changes in Thr and Met would likely impact mTOR as well via SAMTOR and other mechanisms. As antibodies for mTOR substrates exist in flies this could be easily shown; and likely there should be massive inhibition of autophagy. This is particularly important as the authors propose the Akh is induced by autophagy derived-BCAAS in the first place.

3. As a more minor issue, figures have very small "*" indicating significance, making it very difficult to interpret the small figures; this is made even harder as the authors have plotted SD instead of SEM.

Reviewer #2

(Remarks to the Author)
Nishimura et al. successfully measured the exact circulating amount of the Drosophila hormone Akh, which is expected to have a glucagon-like function, by LC-MS/MS. Using this high-resolution assay, they proposed new hypotheses about the metabolic functions of Akh that were not studied in detail before. The authors show that Akh secreted from CC activates the Akh receptor (AkhR), which is mainly expressed in the fat body, and that a negative feedback operates: metabolism driven by AkhR then suppresses Akh secretion. They also show that, under starvation, body BCAAs increase, and the CC is activated by BCAAs, directly or indirectly, to promote Akh secretion. AkhR function in the fat body activates BCAA catabolism and produces GSH, which may prevent a collapse of redox balance caused by activated lipid and protein metabolism and may maintain redox homeostasis. They cross-checked this possibility across different feeding conditions. However, many data in this paper are indirect or superficial to support the claims. Critically, the data presentation is not efficient, so readers who are not experts in metabolism will find it hard to follow the logic. Therefore, my judgment is Major revision. The authors need to deliver the meaning of the various experiments more concisely. Also, some data directly contradict the authors' main claims; additional experiments and explanations are essential for publication.
Major concerns:
1. The paper repeats measurements of Akh levels and many metabolites in AkhR mutants, but a more consistent and logical explanation for the observed values is needed. In Fig. 7h, AkhR activates bcat, which drives BCAA catabolism and

promotes GSH production, reducing the amount of BCAAs released from the fat body. Because BCAAs activate the CC and trigger Akh secretion, the reduction of BCAA release by AkhR signaling acts as a negative feedback to suppress Akh secretion. This seems to be the paper's main message. But the manuscript keeps using AkhR mutants while bringing in different mechanisms each time, instead of keeping to this consistent explanation, which significantly lowers focus. This makes the paper more complex than necessary; a fundamental correction of the figure flow is needed.
2. In Fig. 4a, the changes in amino-acid levels with starvation are shown for control and AkhR1. The basic pattern—BCAAs increase and NEAAs decrease with starvation—appears in both groups. This outcome directly conflicts with the main hypothesis (AkhR-mediated inhibition of BCAA release and the negative feedback on Akh secretion). While two events could happen at the same time in detail, this result confuses the story. A fundamental improvement is needed.
3. The authors show decreased BCAA levels in Atg1 and Atg6 mutants under starvation, but they do not explain clearly how autophagy acts in the proposed BCAA–Akh interaction. Fig. 5b presents an experiment that could inform this point, yet the final model does not show the role of autophagy.
4. In Fig. 6h, ROS levels in AkhR mutants do not increase; they decrease. The authors explain this as a result of decreased overall metabolism in AkhR mutants, but this is not sufficient. A fundamental explanation is needed for why bcat mutants and AkhR mutants differ.
5. The authors also present parts on carbohydrate and lipid metabolism, but these sections distract from the paper's core message: inter-organ communication via BCAAs and Akh–AkhR signaling. These data should be moved to the Supplement. In their place, add experiments that test the BCAA-based hypothesis. As the authors admit, they have not determined whether BCAAs directly activate the CC or act indirectly. A simple ex vivo calcium imaging test on CC can address this. Adding such data would better highlight the core of this paper.

Reviewer #3

(Remarks to the Author)
This paper investigates the regulation and function of the key insect starvation hormone, Akh. A significant literature already exists linking glucagon-like Akh to lipid and carbohydrate metabolism but this paper provides a link to BCAA catabolism. It develops a mass spec method to directly measure Akh and determines that it is subject to negative feedback from its own downstream signalling. The authors also find that BCAA catabolism is regulated by Akh signaling, fine-tunes Akh secretion and is functionally coupled to GSH synthesis and redox homeostasis during starvation.

The authors are to be commended on the successful development and thorough validation of a quantitative MS assay for mature Akh. This method, when combined with genetics, has allowed the authors to add to our knowledge of Akh regulation and function by making a link between Akh signalling in the fat body and BCAA catabolism. However, the paper covers a lot of ground rather superficially and there are multiple major technical shortcoming with the knockdowns. In addition, there are alternative interpretations of the data that are not consistent with the model and there is a major mechanistic gap that has not been addressed. Specific comments are detailed below.

MAJOR:

1) The He and Perrimon labs provided bioassay evidence (PMID: 39747032) that the BCAAs leucine and isoleucine do not trigger AKH release from the larval CC, whereas other amino acids such as threonine do. Even though the authors do highlight larval-adult differences in Fig7, there is still considerable mechanistic doubt on the author's interpretations of their data and also the adult CC model (Fig7h) in which BCAAs act on the CC to stimulate Akh secretion. It is therefore necessary to provide additional experimental evidence showing whether or not BCAAs act directly on the adult CC to stimulate Akh release, perhaps using an ex vivo/explant system. This mechanistic evidence is important for validating the model in Fig7h.

2) All genetic experiments use either whole-body mutants or targeted manipulations that appear to use Gal4 drivers active throughout development. Therefore the adult effects studied here could come with a possible developmental defect. For example, the immunofluorescence stainings in Fig2B seems to show a change in size/length of the CC between wildtype and Akhr mutant, that could be caused by altered development. Moreover, existing literature suggests that Akh signalling can affect nutrient stores during development (Galikova et al 2015, PMID: 26275422) and that Akh signalling is necessary to adapt to suboptimal nutrient conditions (Hughson et al 2021, PMID: 33708137). Given the possibility of developmental effects, many of the genetic manipulations would need to be repeated with adult-specific knockdowns to be sure that phenotypes are not being misinterpreted.

3) The authors introduce Akh as a major catabolic signal required to mobilise nutrient stores especially from the FB. Yet, the data presented argue that Akh signalling is not necessary to mobilise TAG and glycogen stores. In Fig 3c, where the authors show high significance of increased TAG and glycogen at all/many timepoints of starvation, the actual mobilisation (rate of decrease) is fairly identical for glycogen and only marginally smaller for TAG. The main difference, which may also better explain the longer starvation survival is that AkhR mutants start off with bigger nutrient stores, consistent with the existing literature.

4) The requirement of AkhR in the FB to regulate Akh levels stems mainly from the observation that knockdown of AkhR with Cg-Gal4 recapitulates the phenotype. Cg-Gal4 is known to be expressed in hemocytes as well as fat body (Chen et al 2014, PMID: 25417159), so it would be necessary to confirm this result with a cleaner driver such as Lpp-Gal4.

5) The reduced labelling from 15N-leucine in AkhR mutants together with the no change in GSH total levels in AkhR

mutants could indicate glutathione synthesis from amino group donors other than leucine, rather than the authors conclusion of “impaired BCAA-dependent GSH synthesis”.

6) Bcat mutants are shown to have lower NEAA levels during starvation, which the authors interpret as evidence for production of these from BCAAs. However, comparing the Bcat mutant profile during starvation (Fig 6a) with autophagy mutants (Fig 4g), suggests that EAAs are generally dependent on FB autophagy but NEAAs are less dependent on it and some even increase, including Glu - the primary recipient of the amino group of BCAAs as shown in Fig 6c. The conclusion that BCAAs are required to make NEAAs such as Glu is therefore problematic.

7) Existing literature not really cited by the authors shows that Akh signalling in neurons regulates behavioural responses to starvation such as foraging (Yu et al 2016, Jourjinie et al 2016). Hyperactivity/foraging is very likely an important aspect of energy expenditure during starvation and would be expected to be absent in AkhR mutants. This means that the link between Akh signalling and mobilisation of stored nutrients could be very indirect and not necessarily consistent with the authors conclusions.

8) The interpretations in this paper often rely heavily upon large effect sizes in Akhr mutants. How physiological is the Akh hypersecretion (Akhr mutant) effect being studied if, as the authors show, it does not depend upon the same Ampk mechanism as normal secretion?
Furthermore, the statement (line 333-4) is not supported by the evidence in the paper :" these findings strongly support a positive regulatory role of BCAAs in Akh secretion". To make sense of the Cg>hBCAT2 overexpression results, we need comparisons with flies that are wildtype at the AkhR locus. Currently, it is not possible to see how hBCAT2 functions in the physiological context where Akh negative feedback has not been removed.

9) In Fig 6d there is substantial labelling of many NEAAs from 15N-leucine. This high NEAA labelling may, unfortunately, indicate a profound metabolic rewiring caused by adding a bolus of leucine to the starvation medium. The interpretation therefore has to be more cautious, along the lines of “Leucine can act as a nitrogen donor for NEAA synthesis during starvation”.

MINOR:

The statement that "AkhR is mainly expressed in the fat body” is an overstatement as FlyAtlas 2 shows AkhR expressed highly in several other tissues including heart, eye and carcass.

The model in Fig7h shows that Akhr activation leads to upregulation of Bcat and the evidence indicates that this is at the mRNA level. The authors have not discussed what candidate mechanisms might be responsible for this link.

Line 70-71. The following statement is confusing. "Unlike stored carbohydrates and lipids, amino acids serve dual roles as both structural components and metabolic fuels". Surely lipids contribute to cell membranes - which would be a structural role.

The mating status of the adult flies is not mentioned. important, especially given the sex-specific effects being reported.

Reviewer #4

(Remarks to the Author)
I co-reviewed this manuscript with one of the reviewers who provided the listed reports. This is part of the Nature Communications initiative to facilitate training in peer review and to provide appropriate recognition for Early Career Researchers who co-review manuscripts.

Version 1:

Reviewer comments:

Reviewer #1

(Remarks to the Author)
The authors have robustly responded to the reviews, especially by performing new stable isotope labeling experiments to show that Bcat expression controls BCAA flux and showing mTOR activation. I think they could make a stronger statement regarding the suppression of autophagy by mTOR as this is fairly standard biology. In any case the authors should be applauded for their comprehensive response to the reviewer critiques.

Reviewer #2

(Remarks to the Author)
Overall, the authors appear to have carefully addressed the major concerns raised in the previous round of review and have performed the requested experiments in a diligent manner. This manuscript is of high significance, particularly due to the

novelty of the quantitative Akh measurement approach and the depth of the metabolic analyses enabled by this method. The study provides compelling evidence that the Akh–AkhR signaling axis, a glucagon-like endocrine pathway in Drosophila, exerts broad metabolic effects, including regulation of BCAA metabolism, autophagy, and redox homeostasis, and mediates inter-organ communication between the corpora cardiaca (CC) and the fat body.
While the overall quality and importance of the work are clear, the previous version of the manuscript suffered from substantial complexity, with multiple lines of evidence presented in parallel and, in some cases, apparently conflicting observations listed without sufficient contextual explanation. In the revised manuscript, many of these issues have been substantially improved. The data are better organized, and several previously fragmented or confusing sections have been clarified, resulting in a more coherent narrative.
That said, I believe the manuscript would benefit from a few additional, relatively minor revisions that would further improve clarity and accessibility for readers. I therefore recommend minor revision, with the following specific points to be addressed:
1. Rationale for extensive use of AkhR mutant lines
In the rebuttal, the authors provide a clear and convincing explanation for their repeated use of AkhR mutant lines, particularly in the context of revealing the physiological negative feedback structure of the Akh–AkhR axis. However, this rationale is not explicitly stated in the manuscript itself. I recommend that the authors incorporate a brief explanation into the main text, clarifying the primary purpose of using AkhR mutants and how this approach helps expose the underlying feedback regulation.
2. Interpretation of amino acid levels under starvation
In the rebuttal, the authors appropriately note that amino acid levels measured under starvation conditions reflect a net effect of multiple processes, including autophagy/protein degradation, protein synthesis, and downstream metabolic consumption. This important clarification should be explicitly incorporated into the Results section. Without this explanation, the initial presentation of the amino acid data risks confusing readers, as net pool size changes may be mistakenly interpreted as direct readouts of Akh–AkhR signaling or CC–fat body inter-organ communication. A clear statement at the point where these data are first introduced would greatly aid interpretation.
3. Direct testing of BCAA effects on CC cell activity
The authors attempted to directly test whether BCAAs can acutely regulate CC cell activity, as requested in the first revision. Although these experiments yielded negative results, I consider this effort to be meaningful and valuable. I encourage the authors to present these data more fully—properly quantified and statistically analyzed—in a supplementary figure. Moreover, the interpretation of these negative results, currently discussed in the rebuttal, should be further elaborated and incorporated into the Discussion. Demonstrating that this possibility was directly tested, even with negative outcomes, strengthens the overall rigor of the study.
If these three points are adequately addressed, I believe that the manuscript will be suitable for publication in Nature Communications and will make a strong contribution to the field.

Reviewer #3

(Remarks to the Author)
The revised manuscript now satisfactorily addresses several of our major points (2-5 and 7-9). However, three major points (1, 3, 6) have not been adequately addressed and/or continue to cast serious doubt upon the authors interpretations and large parts of their mechanistic model in Fig. 7h (now 8h):

Major point 1) Along with another reviewer, we raised the important point that there was no solid evidence to support the CC model (Fig7h) in which BCAAs act on the CC to stimulate Akh secretion. The authors have now done well to conduct new ex vivo calcium imaging and Tor signaling experiments to address this issue. Unfortunately, however, these new experiments failed to provide any evidence that BCAAs act on the CC to stimulate Akh signalling. So the most parsimonious explanation, in line with a previous study (PMID: 39747032), is that the CC does not respond to BCAAs. This part of the Fig 7h, now 8h, model is therefore incorrect.

Major point 3) In light of our comments here, the authors seem to have reinterpreted their data such that Akh signaling is no longer required for mobilization of TAG (and glycogen) stores. This part of the Fig 7h, now 8h, model is therefore questionable. The authors replacement of the text with “regulates/modulates” rather than “is required for” is still an unsubstantiated claim.

Major point 6) This criticism has not been adequately addressed. In both the original and revised manuscript there continues to be no solid evidence for yet another key part of the Fig 7h model, namely that NEAAs (and GSH) are synthesized from BCAAs via autophagy and in response to Akh signaling. Fig 5c shows that autophagy mutants lower BCAA levels but increase Glu levels. Thus, it cannot be ruled out that other nitrogen donors exist and can be utilised to make NEAAs and possibly GSH, which the authors don’t test.

In conclusion, the authors responses to major points 1,3 and 6 do not provide sufficient missing evidence to plug numerous mechanistic gaps in the Fig. 8h model. On balance, we therefore regretfully conclude that what remains intact in the model is, at best, borderline in terms of any substantial mechanistic advance.

Reviewer #4

(Remarks to the Author)
I co-reviewed this manuscript with one of the reviewers who provided the listed reports. This is part of the Nature

Communications initiative to facilitate training in peer review and to provide appropriate recognition for Early Career Researchers who co-review manuscripts.

Version 2:

Reviewer comments:

Reviewer #2

(Remarks to the Author)
After carefully reviewing the authors' responses to the second round of revision, I find that the manuscript has reached a level suitable for publication. The authors have addressed the reviewers' comments with diligence and thoroughness, providing thoughtful point-by-point responses. I therefore recommend that this manuscript be accepted for publication in Nature Communications.

Reviewer #3

(Remarks to the Author)
The authors have addressed our criticisms as much as is possible with textual changes but without significant extra experiments. I really hope that this will help to future proof the main conclusions of the manuscript.

Reviewer #4

(Remarks to the Author)
I co-reviewed this manuscript with one of the reviewers who provided the listed reports. This is part of the Nature Communications initiative to facilitate training in peer review and to provide appropriate recognition for Early Career Researchers who co-review manuscripts.

Open Access This Peer Review File is licensed under a Creative Commons Attribution 4.0 International License, which permits use, sharing, adaptation, distribution and reproduction in any medium or format, as long as you give appropriate credit to the original author(s) and the source, provide a link to the Creative Commons license, and indicate if changes were made.
In cases where reviewers are anonymous, credit should be given to 'Anonymous Referee' and the source.
The images or other third party material in this Peer Review File are included in the article's Creative Commons license, unless indicated otherwise in a credit line to the material. If material is not included in the article's Creative Commons license and your intended use is not permitted by statutory regulation or exceeds the permitted use, you will need to obtain permission directly from the copyright holder.
To view a copy of this license, visit https://creativecommons.org/licenses/by/4.0/

We are grateful to all reviewers for taking the time to review our manuscript and provide us constructive comments. We have taken all the comments into consideration and have made appropriate changes to the manuscript. Our point-by-point response appears below (in **blue**). We hope that the revised manuscript is now suitable for publication in *Nature Communications*.

Reviewer #1 (Remarks to the Author):
This is a really interesting article; overall, the authors identify that BCAA catabolism is controlled by Akh, a glucagon like peptide, by inducing the expression of the first step of BCAA catabolism, with downstream effects on BCAA levels and glutathione and oxidative stress. However, there are strong gaps in the mechanisms explored, and I find the overall writing very confusing - I think cause and effect are not well distinguished in many of the proposed mechanisms. Some of the things I was thinking about that suggest to me that revision is required:

1. My main critique is that the proposed mechanism of action of Akh makes no sense biochemically. Bcat is the first step of BCAA catabolism, but Bcat is freely reversible and is not the rate limiting step; that is the next step in BCAA catabolism, regulated by BCKDH. As such, the roughly 2x difference in Bcat gene expression resulting from Akh loss should have no biologically significant effect. IF Akh is working as proposed, it must also be regulating the fly homologues of Bckdh or Bckdk; and the authors should examine the gene and protein expression of the entire BCAA catabolic pathway to be comprehensive. Flux could also be examined with stable isotopes to give a thorough assessment.

Response: Thank you for raising this important issue. We agree that Bcat is reversible and is not generally considered the rate-limiting step in BCAA catabolism, whereas flux is often controlled at the BCKDH step. To address this concern directly, we expanded our analysis to include multiple BCKDH components and added stable-isotope flux data demonstrating functional consequences of Akh-dependent Bcat regulation during starvation.

First, based on the single-cell Fly Cell Atlas database (Li et al., 2022), components of BCKDH (E1α/Bckdha, E1β/Bckdhb, E2/Dbct, and E3/Dld) are broadly expressed across tissues (**Fig. S6a**). In contrast, *Bcat* expression is relatively restricted to specific tissues, including the fat body. Although the regulatory phosphorylation site is conserved in *Drosophila* Bcat, most insects, including *Drosophila*, lack clear orthologs of the kinase BCKDK and the phosphatase PPM1K (Marygold, 2024). Moreover, there is currently no evidence that *Drosophila* Bcat is

regulated by phosphorylation. Notably, as shown in **Fig. S6b**, the Developmental Proteome database (Casas-Vila et al., 2017) indicates that Bcat protein abundance is lower than that of BCKDH components in one-week-old adult flies (approximately 4.5–6 times lower in males). These observations are consistent with the idea that, in tissues where *Bcat* is limiting, modulation of *Bcat* expression could influence overall BCAA catabolic throughput. Our expression analysis shows that *Bckdhb* and *Dbct*, but not *Bckdha*, mRNA levels were up-regulated by starvation, yet the induction was modest compared with *Bcat* (**Fig. 4f**). *AkhR* mutants failed to induce starvation-dependent *Bckdhb*. However, *Akh* overexpression was not sufficient to induce *Bckdhb* expression (**Fig. S6c**), suggesting that Akh signaling does not regulate BCKDH expression. Together, these results indicate that Bcat is the primary Akh-responsible node at the expression level, while BCKDH components are comparatively stable at least transcriptionally in our experimental context.

Second, we clarify that the net direction of the Bcat reaction depends strongly on substrate/product availability. In general, glutamate is abundant relative to α-ketoglutarate, whereas branched-chain keto acids (BCKAs) are much lower than BCAAs. In our LC-MS/MS measurements, BCKAs were at or below the detection limit even under starvation, suggesting that substrate/product availability is strongly biased toward the BCAA side. Thus, although Bcat is freely reversible in vitro, starvation-induced up-regulation of *Bcat* is expected to increase pathway throughput toward downstream BCKDH-dependent catabolism, thereby influencing overall BCAA catabolic flux.

Third, to test whether the ~2-fold reduction in *Bcat* expression in *AkhR* mutants is biologically meaningful, we performed flux analysis using 13C-labeled leucine (**Fig. 7c**). Consistent with our 15N-labeling results, the fraction of 13C-labeled TCA cycle intermediates (citrate, aconitate, fumarate, and malate) was decreased in *AkhR* mutants and was more severely reduced in *Bcat* mutants (**Fig. 7e**). These results provide direct functional evidence that Akh-dependent regulation of *Bcat* expression is critical for BCAA catabolism during starvation.

In summary, we expanded the revised manuscript to (i) document expression changes in multiple BCKDH components, (ii) clarify the substrate-dependent biochemical basis for a nutritionally regulated Bcat step, and (iii) provide stable-isotope flux evidence demonstrating functional consequences of Akh-dependent Bcat regulation. We believe that these additions significantly strengthen the revised manuscript.

2. I was surprised that mTOR was not discussed, as the massive increase in BCAA levels should

be more than sufficient to effect mTOR via Sestrin binding, and 2-3 changes in Thr and Met would likely impact mTOR as well via SAMTOR and other mechanisms. As antibodies for mTOR substrates exist in flies this could be easily shown; and likely there should be massive inhibition of autophagy. This is particularly important as the authors propose the Akh is induced by autophagy derived-BCAAs in the first place.

Response: To address this issue, we examined the phosphorylation level of S6K in *AkhR* mutants by Western blotting. In control flies, pS6K levels in abdominal carcasses were significantly decreased upon starvation (**Fig. 5f, g**), indicating reduced mTOR activity. In contrast, as the reviewer pointed out, *AkhR* mutants exhibited higher pS6K levels than control flies under starved conditions, although these levels remained lower than in fed conditions. Notably, pAkt levels in *AkhR* mutants were comparable to those in control flies. Together, these results suggest that mTOR activity is partially maintained in *AkhR* mutants during starvation, most likely reflecting elevated BCAA levels rather than changes in insulin/PI3K signaling. Because autophagy-derived amino acids can contribute to mTORC1 reactivation, thereby forming a negative-feedback loop that limits autophagy during prolonged starvation (Deleyto-Seldas and Efeyan, 2021), our results are consistent with the possibility that autophagy is partly attenuated in *AkhR* mutants under starved conditions. However, we note that we did not directly quantify autophagy flux in this study; therefore, we have revised the text to describe this interpretation cautiously.

Finally, we agree that mTOR could, in principle, provide a mechanistic link between elevated amino acids and endocrine outputs. We address the potential relationship between BCAA levels and Akh secretion in our response to Reviewer #2, comment 5 (see below), where we discuss this point in the appropriate context.

3. As a more minor issue, figures have very small "*" indicating significance, making it very difficult to interpret the small figures; this is made even harder as the authors have plotted SD instead of SEM.

Response: Thank you for the comment. We have revised all figures by increasing the font size of the asterisks indicating statistical significance. To improve readability in dot plots, we also added horizontal comparison bars and placed the asterisks above these bars, which clearly distinguish the significance markers from the plotted data points. These changes make the figures easier to interpret, particularly when panels are displayed at reduced size.

Reviewer #2 (Remarks to the Author):

Nishimura et al. successfully measured the exact circulating amount of the Drosophila hormone Akh, which is expected to have a glucagon-like function, by LC-MS/MS. Using this high-resolution assay, they proposed new hypotheses about the metabolic functions of Akh that were not studied in detail before. The authors show that Akh secreted from CC activates the Akh receptor (AkhR), which is mainly expressed in the fat body, and that a negative feedback operates: metabolism driven by AkhR then suppresses Akh secretion. They also show that, under starvation, body BCAAs increase, and the CC is activated by BCAAs, directly or indirectly, to promote Akh secretion. AkhR function in the fat body activates BCAA catabolism and produces GSH, which may prevent a collapse of redox balance caused by activated lipid and protein metabolism and may maintain redox homeostasis. They cross-checked this possibility across different feeding conditions.

However, many data in this paper are indirect or superficial to support the claims. Critically, the data presentation is not efficient, so readers who are not experts in metabolism will find it hard to follow the logic. Therefore, my judgment is Major revision. The authors need to deliver the meaning of the various experiments more concisely. Also, some data directly contradict the authors' main claims; additional experiments and explanations are essential for publication.

Major concerns:

1. The paper repeats measurements of Akh levels and many metabolites in AkhR mutants, but a more consistent and logical explanation for the observed values is needed. In Fig. 7h, AkhR activates bcat, which drives BCAA catabolism and promotes GSH production, reducing the amount of BCAAs released from the fat body. Because BCAAs activate the CC and trigger Akh secretion, the reduction of BCAA release by AkhR signaling acts as a negative feedback to suppress Akh secretion. This seems to be the paper's main message. But the manuscript keeps using AkhR mutants while bringing in different mechanisms each time, instead of keeping to this consistent explanation, which significantly lowers focus. This makes the paper more complex than necessary; a fundamental correction of the figure flow is needed.

Response: Thank you for these constructive comments. We agree that the manuscript should present a more consistent, hypothesis-driven "working model" and align the figure flow with that model to improve focus and readability. Our rationale for using *AkhR* mutants throughout the study is precisely to unmask the physiological negative-feedback architecture that operates in wild-type flies. In wild-type conditions, the feedback loop between the fat body and CC cells buffers both Akh secretion and downstream metabolite levels, making it difficult to observe

large effect sizes. Thus, the feedback regulation itself is a central physiological mechanism that we aim to define in this manuscript.

We also agree with the reviewer that the current presentation can appear "data-driven" in a way that introduces multiple mechanisms without a single guiding explanation. To address this concern, we revised the manuscript to (i) state the working model earlier, (ii) explicitly connect each dataset to specific elements of the model, and (iii) reorganize the figure flow to follow the causal logic of the feedback loop. In the revised version, we present a unified model in which AkhR–Bcat–BCAA catabolism in the fat body reduces BCAA release, thereby providing negative feedback to the CC cells and suppressing Akh secretion. Notably, the new data generated during revision strengthened the causal chain underlying this model. Importantly, we were able to reposition several datasets that previously read as somewhat disconnected observations, most notably the autophagy results, so that they now directly test and reinforce specific steps of the feedback model. For example, inhibiting fat body autophagy (*Atg1* knockdown) suppressed the starvation-induced increase in BCAA levels and led to a significant reduction in circulating Akh during starvation (see below, Reviewer #2, comment 3), providing direct support for the step linking fat body-derived nutrients to Akh output. Together with our flux and signaling analyses (e.g., 13C-leucine tracing and pS6K measurements), these additions help consolidate the Results around a single feedback framework and reduce the need to invoke separate, parallel explanations.

Finally, we note that Akh signaling is well characterized to regulate lipid and carbohydrate metabolism during starvation in insects, and these pathways can influence systemic endocrine outputs. Rather than introducing these metabolites as separate mechanisms, we have revised the text to explain how they fit within (or are secondary to) the core feedback model we identify here, in which fat body BCAA metabolism feeds back to the CC cells to restrain Akh secretion. We believe these revisions substantially improve the manuscript's clarity and focus without sacrificing the breadth of the dataset, which we still expect will be a valuable resource for the community.

2. In Fig. 4a, the changes in amino-acid levels with starvation are shown for control and AkhR1. The basic pattern—BCAAs increase and NEAAs decrease with starvation—appears in both groups. This outcome directly conflicts with the main hypothesis (AkhR-mediated inhibition of BCAA release and the negative feedback on Akh secretion). While two events could happen at the same time in detail, this result confuses the story. A fundamental improvement is needed.

Response: Thank you for the comment. We apologize that the presentation and interpretation of **Fig. 4a** were not sufficiently clear in the original manuscript. We agree that time-course changes in amino-acid abundance (i.e., pool sizes), as summarized in the heat map, do not directly report metabolic flux, because amino-acid levels during starvation reflect the net balance between production (e.g., autophagy/protein degradation) and consumption (e.g., protein synthesis and downstream metabolism). Therefore, a shared qualitative trend in pool sizes does not necessarily contradict a model in which flux differs between genotypes.

Importantly, although the qualitative pattern (BCAAs increase and NEAAs decrease upon starvation) is observed in both control and *AkhR* mutants, the magnitude of these changes differs between genotypes: *AkhR* mutants show a further increase in BCAAs and a further decrease in NEAAs compared with controls (**Fig. 4a, b**). This quantitative divergence is consistent with our model that AkhR signaling promotes BCAA catabolism via Bcat induction. In controls, starvation induces substantial amino-acid production (likely dominated by autophagy/protein degradation), and BCAA production can exceed BCAA catabolic capacity, which can result in a net increase in BCAA pool even when catabolism is active. In *AkhR* mutants, reduced Bcat expression is expected to further limit BCAA catabolic throughput, leading to greater net BCAA accumulation.

Similarly, NEAA pools can decrease in both genotypes during starvation because NEAAs generated from multiple sources (including BCAA catabolism) can be rapidly consumed and further metabolized, and net consumption may exceed net production under these conditions. Thus, the shared qualitative trend does not contradict the model: rather, the stronger quantitative shift in *AkhR* mutants is consistent with an AkhR-dependent contribution of BCAA catabolism to amino-acid homeostasis. We have revised the Results text and Discussion to clarify this point and to avoid over-interpreting steady-state pool sizes as direct evidence of flux.

In addition, to improve data presentation and reduce confusion, we now show NEAA profiles together with BCAA profiles for direct comparison (**Fig. 4b**). We also added a schematic of the working model and clearly indicate which prediction is tested in each figure panel (**Fig. 5a**). These revisions were made specifically to align the figure flow with the central feedback model and to make the interpretation of **Fig. 4a** more transparent.

3. The authors show decreased BCAA levels in Atg1 and Atg6 mutants under starvation, but they do not explain clearly how autophagy acts in the proposed BCAA–Akh interaction. Fig. 5b presents an experiment that could inform this point, yet the final model does not show the role

of autophagy.

Response: To clarify the relationship between autophagy-derived BCAAs and Akh secretion, we analyzed circulating Akh levels in starved flies in which fat body autophagy was inhibited by *Atg1* knockdown. In the original manuscript, we showed that inhibition of fat body autophagy suppresses the starvation-induced increase in BCAA levels and lipolysis (**Fig. 5d**). Building on these indirect observations, we directly measured circulating Akh levels and found that they were significantly reduced during starvation when fat body autophagy was inhibited (**Fig. 5e**). Together, these data support a model in which fat body autophagy contributes to the starvation-induced rise in BCAA levels, which in turn promotes Akh secretion and downstream lipolysis.

We agree that the role of autophagy should be made more explicit and easier to locate for readers. Accordingly, although autophagy was already included in the final model (**Fig. 8h**), we added an additional working-model schematic at an earlier figure (**Fig. 5a**) to highlight how autophagy contributes to the BCAA-Akh interaction during starvation.

Finally, we note that autophagy-BCAA-Akh relationship is embedded in a feedback architecture: elevated BCAAs in *AkhR* mutants could potentially attenuate autophagy via mTOR signaling. Consistent with this possibility, we show that mTOR activity (assessed by pS6K) is higher in *AkhR* mutants than in control flies under starvation (**Fig. 5f, g**). We have revised the text to clarify how this potential feedback is accommodated in the model. Further discussion of the mTOR-related aspects of this feedback is provided in our response to Reviewer #1, comment 2.

4. In Fig. 6h, ROS levels in AkhR mutants do not increase; they decrease. The authors explain this as a result of decreased overall metabolism in AkhR mutants, but this is not sufficient. A fundamental explanation is needed for why bcat mutants and AkhR mutants differ.

Response: We agree that the divergent ROS phenotypes between *AkhR* mutants and *Bcat* mutants require a clearer explanation. Although the full mechanistic basis remains to be determined, we performed additional analyses to better define the differences between the two genotypes. We also note that the ROS readout (**Fig. 7g, h**) reflects a tissue-level fluorescence signal (CellROX in the fat body), whereas protein carbonyls and MDA (**Fig. 7f**) report whole-organism oxidative damage. These readouts do not necessarily change in parallel, and thus apparent differences can reflect both tissue specificity and the distinct biology captured by each

assay.

Because Akh signaling has been shown to regulate antioxidant gene expression partly through the transcription factor Foxo (Kodrik et al., 2015), we analyzed the expression of several oxidative stress-related genes. Under fed conditions, *AkhR* mutants showed decreased expression of all superoxide dismutases (*Sod1–3*) and catalase (*Cat*), whereas under starvation only *Sod2* was significantly decreased (**Fig. S8c**). In contrast, *Bcat* mutants exhibited a stronger and broader reduction of antioxidant gene expression: *Sod2*, *Sod3*, and *Cat* were significantly decreased under both fed and starved conditions. These expression patterns suggest that *Bcat* mutants have a more pronounced impairment in organismal antioxidant capacity than *AkhR* mutants, which could contribute to the higher ROS levels observed in *Bcat* mutants.

Consistent with increased oxidative stress in *Bcat* mutants, we also observed strong up-regulation of *GstD1* and *GstD2*, canonical oxidative stress-response genes, which matches the observed increase in ROS levels. By contrast, the comparatively modest changes in antioxidant gene expression under starvation in *AkhR* mutants may help explain why ROS levels do not increase in this genotype. In addition, because *Akh* mutants have been reported to be sensitive to paraquat exposure (Galikova et al., 2015), we interpret the Akh pathway as contributing to stress resistance in a context-dependent manner.

We therefore revised the Results to clarify that the reduced ROS signal in *AkhR* mutants is likely influenced by both (i) altered redox/antioxidant gene regulation and (ii) reduced endogenous ROS production associated with decreased overall metabolic activity, whereas *Bcat* mutants show stronger evidence of compromised antioxidant capacity and oxidative-stress responses.

5. The authors also present parts on carbohydrate and lipid metabolism, but these sections distract from the paper's core message: inter-organ communication via BCAAs and Akh–AkhR signaling. These data should be moved to the Supplement. In their place, add experiments that test the BCAA-based hypothesis. As the authors admit, they have not determined whether BCAAs directly activate the CC or act indirectly. A simple ex vivo calcium imaging test on CC can address this. Adding such data would better highlight the core of this paper.

Response: Thank you for the comment. We agree that the core message of this study is inter-organ communication via BCAAs and Akh–AkhR signaling. Regarding the presentation of carbohydrate and lipid metabolism data, we address this point in detail in our response to Reviewer #2, comment 1.

To address whether adult CC cells directly respond to BCAAs, we performed ex vivo calcium imaging as suggested. As a positive control, we found that depolarization triggered by elevated potassium significantly increased GCaMP signals (**Figure R1 for reviewer**), indicating that ex vivo calcium imaging worked in our experimental conditions. However, CC cells did not respond to elevated BCAA levels under these conditions. Importantly, we also failed to observe calcium signals upon addition of threonine or glycine, which have been reported to stimulate larval CC cells (Ahmad et al., 2025).

Figure R1 for reviewer

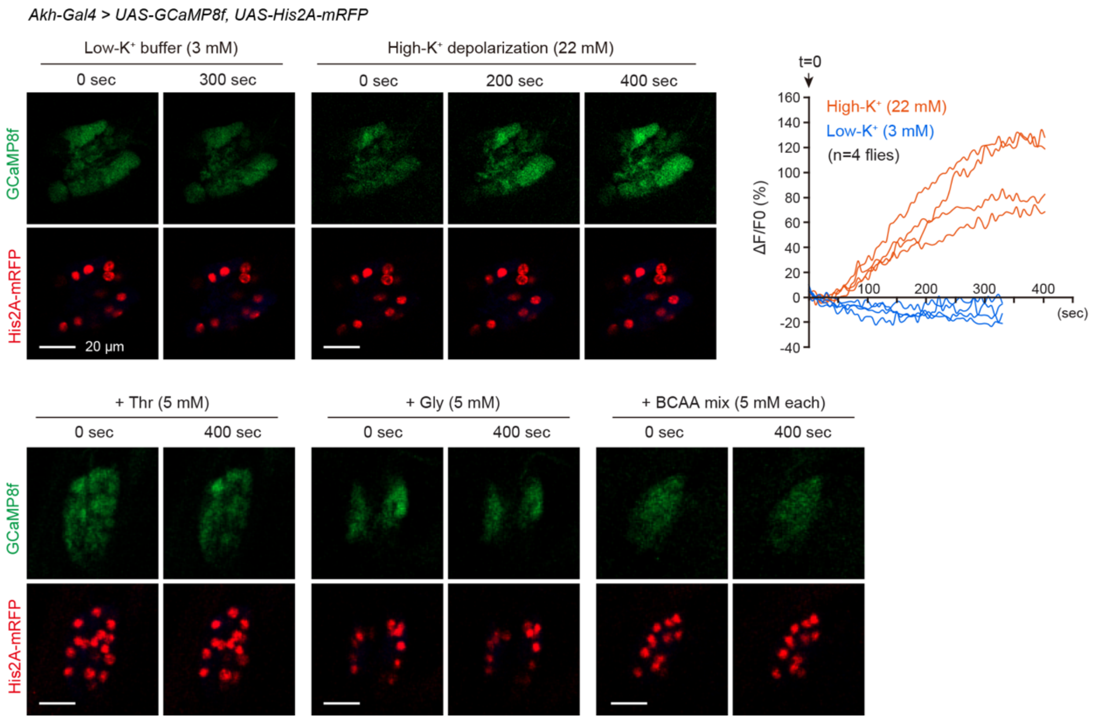

We note that ex vivo calcium imaging has been extensively performed in larval CC cells (Kim and Rulifson, 2004; Braco et al., 2012; Guntur et al., 2015; Pauls et al., 2021; Ahmad et al., 2025), whereas, to our knowledge, there are no established protocols or published studies demonstrating robust stimulus-evoked calcium responses in adult CC cells. In this study, we revealed stage-dependent differences in CC cell responses to starvation (see also our response to Reviewer #3, comment 2), and thus it is possible that our culture conditions are not optimal for detecting amino-acid–dependent activation in adult CC cells. Moreover, it remains unknown whether a direct action of BCAAs on CC cells, if it exists, would necessarily involve an immediate calcium signal. Therefore, the absence of a detectable calcium response in adult CC

cells should be interpreted cautiously, and it does not allow us to conclude that BCAAs act only indirectly on the adult CC cells.

We also explored whether mTOR signaling, a well-known mediator of leucine sensing, could mediate amino-acid effects in adult CC cells. However, temporal manipulation of mTOR activity in adult CC cells markedly affected Akh mRNA and peptide levels, confounding secretion assays by altering Akh production. To acutely suppress mTOR activity during starvation, we performed rapamycin-feeding experiments. Rapamycin feeding only slightly suppressed Akh hypersecretion during starvation in *AkhR* mutants (**Fig. 5h**), yet total Akh and Akh-GK levels still depleted almost completely after two days of starvation. These results suggest that mTOR inhibition is not sufficient to account for the Akh hypersecretion phenotype in *AkhR* mutants, at least under our experimental conditions. Together, these additional data help constrain possible mechanisms by which BCAAs influence Akh secretion, although we agree that the current evidence does not yet establish the precise directness and signaling modality of BCAA action on adult CC cells. We therefore describe this point cautiously in the revised manuscript and highlight it as an important question for future work.

Reviewer #3 (Remarks to the Author):
This paper investigates the regulation and function of the key insect starvation hormone, Akh. A significant literature already exists linking glucagon-like Akh to lipid and carbohydrate metabolism but this paper provides a link to BCAA catabolism. It develops a mass spec method to directly measure Akh and determines that it is subject to negative feedback from its own downstream signalling. The authors also find that BCAA catabolism is regulated by Akh signaling, fine-tunes Akh secretion and is functionally coupled to GSH synthesis and redox homeostasis during starvation.

The authors are to be commended on the successful development and thorough validation of a quantitative MS assay for mature Akh. This method, when combined with genetics, has allowed the authors to add to our knowledge of Akh regulation and function by making a link between Akh signalling in the fat body and BCAA catabolism. However, the paper covers a lot of ground rather superficially and there are multiple major technical shortcoming with the knockdowns. In addition, there are alternative interpretations of the data that are not consistent with the model and there is a major mechanistic gap that has not been addressed. Specific comments are detailed below.

MAJOR:

1) The He and Perrimon labs provided bioassay evidence (PMID: 39747032) that the BCAAs leucine and isoleucine do not trigger AKH release from the larval CC, whereas other amino acids such as threonine do. Even though the authors do highlight larval-adult differences in Fig7, there is still considerable mechanistic doubt on the author's interpretations of their data and also the adult CC model (Fig7h) in which BCAAs act on the CC to stimulate Akh secretion. It is therefore necessary to provide additional experimental evidence showing whether or not BCAAs act directly on the adult CC to stimulate Akh release, perhaps using an ex vivo/explant system. This mechanistic evidence is important for validating the model in Fig7h.

Response: Thank you for raising this important point and for highlighting the prior larval CC bioassay study (Ahmad et al., 2025). In response, we performed ex vivo calcium imaging in adult CC cells to test whether BCAAs directly evoke an acute activation signal (**Figure R1 for reviewer**). While high K+ robustly increased GCaMP signals (positive control), we did not detect a calcium response to elevated BCAAs under our adult CC imaging conditions. We also did not observe responses to threonine or glycine, which have been reported to stimulate larval CC cells (Ahmad et al., 2025), suggesting that adult CC cells may differ in responsiveness and/or that current culture conditions are not optimized to capture amino-acid–evoked responses in adults. Importantly, the absence of an acute calcium signal does not by itself rule out direct regulation via other signaling modalities. We therefore revised the manuscript to frame the directness of BCAA action on adult CC cells as an open question (See also our response to Reviewer #2, comment 5 for additional details).

2) All genetic experiments use either whole-body mutants or targeted manipulations that appear to use Gal4 drivers active throughout development. Therefore the adult effects studied here could come with a possible developmental defect. For example, the immunofluorescence stainings in Fig2B seems to show a change in size/length of the CC between wildtype and Akhr mutant, that could be caused by altered development. Moreover, existing literature suggests that Akh signalling can affect nutrient stores during development (Galikova et al 2015, PMID: 26275422) and that Akh signalling is necessary to adapt to suboptimal nutrient conditions (Hughson et al 2021, PMID: 33708137). Given the possibility of developmental effects, many of the genetic manipulations would need to be repeated with adult-specific knockdowns to be sure that phenotypes are not being misinterpreted.

Response: To address the reviewer's concern that developmental effects could contribute to the adult phenotypes, we performed additional analyses and adult-specific manipulations.

First, we conducted adult-specific knockdown of *AkhR*, and the results are described below (see comment 4).

Second, we performed quantitative analyses of CC cell morphology to examine potential developmental defects. In one-week-old adults, CC cell number and cell size in *AkhR* and *Bcat* mutants were indistinguishable from controls (**Fig. S10b and c**), indicating that these genotypes do not cause detectable developmental defects in CC cells. Regarding the apparent difference in the overall size/length of the CC cell cluster in **Fig. 2b**, we note that the CC cluster is attached to one side of the esophagus, and its apparent size in the X–Y plane is highly sensitive to mounting orientation; thus, variation in sample orientation during preparation can affect the perceived cluster length/area in immunofluorescence images.

Third, because the reviewer also raised the possibility of developmental effects on metabolism, we examined Akh levels and nutrient stores in newly eclosed adults. In newly eclosed *AkhR* mutants, total protein, total Akh, and Akh-GK levels were comparable to controls (**Fig. S10a**), suggesting that the decreased total Akh levels and increased Akh-GK levels observed in one-week-old adults (**Fig. 2a and S3b**) arise during adulthood rather than from developmental defects. Similarly, newly eclosed *Bcat* mutants showed normal total protein and Akh-GK levels, while total Akh was elevated (**Fig. S10a**); this contrasts with the marked reduction of total Akh and Akh-GK observed in one-week-old adults (**Fig. 6c**), again supporting an adult-onset phenotype.

We also assessed TAG levels at eclosion and found that TAG levels in newly eclosed *AkhR* and *Bcat* mutants were indistinguishable from controls (**Fig. S10a**). Thus, the increased TAG in *AkhR* mutants and decreased TAG in *Bcat* mutants observed in adults (**Fig. 3c and 6e**) represent adult-onset metabolic phenotypes. Notably, BCAA levels were already elevated in newly eclosed *Bcat* mutants (**Fig. S10a**), suggesting that elevated BCAAs per se are not sufficient to alter TAG levels during development. Together, these results support our conclusion that the AkhR-dependent feedback regulation described in this study is primarily adult-specific.

We believe that these additional results strengthen our conclusions and validate an adult-onset role of Akh signaling and BCAA-dependent regulation of Akh output.

3) The authors introduce Akh as a major catabolic signal required to mobilise nutrient stores especially from the FB. Yet, the data presented argue that Akh signalling is not necessary to

mobilise TAG and glycogen stores. In Fig 3c, where the authors show high significance of increased TAG and glycogen at all/many timepoints of starvation, the actual mobilisation (rate of decrease) is fairly identical for glycogen and only marginally smaller for TAG. The main difference, which may also better explain the longer starvation survival is that AkhR mutants start off with bigger nutrient stores, consistent with the existing literature.

Response: We thank the reviewer for this comment and agree with the interpretation. Our data indicate that Akh signaling is not strictly required for mobilization of TAG and glycogen stores during starvation, as the overall rate of decline is similar between genotypes (**Fig. 3c**). Rather, the most prominent difference is that *AkhR* mutants start with larger nutrient stores, which is consistent with prior literature and may contribute to their extended starvation survival.

We also agree that lipolysis and glycogenolysis are not regulated solely by Akh signaling. Previous studies have shown that Akh signaling and the bmm lipase can act redundantly to promote lipolysis in the adult fat body (Gronke et al., 2007). Glycogen breakdown is likewise regulated by multiple layers of control, including hormone-dependent regulation of glycogenolytic enzymes via phosphorylation as well as allosteric regulation that reflects intracellular metabolic state (e.g., energy charge and glucose availability) (Roach et al., 2012). Accordingly, in this manuscript we intend to state that Akh signaling modulates these catabolic processes, rather than being necessary for mobilization per se. To avoid confusion, we revised the text and figure interpretation to use more precise wording (e.g., “regulates/modulates” rather than “is required for”).

4) The requirement of AkhR in the FB to regulate Akh levels stems mainly from the observation that knockdown of AkhR with Cg-Gal4 recapitulates the phenotype. Cg-Gal4 is known to be expressed in hemocytes as well as fat body (Chen et al 2014, PMID: 25417159), so it would be necessary to confirm this result with a cleaner driver such as Lpp-Gal4.

Response: To address this concern regarding the specificity of *Cg-Gal4*, we performed adult-stage, fat body-specific knockdown experiments using two independent fat body drivers, *Lpp-Gal4* and *r4-Gal4*. Transient (adult-onset) knockdown of *AkhR* after eclosion led to a reduction in total Akh levels upon starvation (**Fig. S3e**), recapitulating the conclusion that fat body AkhR is required to regulate starvation-induced Akh output. These results indicate that Akh signaling in the fat body remotely regulates Akh secretion from the CC cells.

5) The reduced labelling from 15N-leucine in AkhR mutants together with the no change in

GSH total levels in AkhR mutants could indicate glutathione synthesis from amino group donors other than leucine, rather than the authors conclusion of "impaired BCAA-dependent GSH synthesis".

Response: We would like to clarify that our conclusion is based on the starvation conditions, as we performed 15N-leucine tracing into GSH only under starvation. Therefore, the observation that total GSH levels are unchanged in *AkhR* mutants under fed conditions is not the relevant comparison for interpreting the reduced 15N-leucine labeling. Consistent with the tracing data, we found that total GSH levels in *AkhR* mutants are reduced specifically under starvation. Thus, although other amino donors may contribute to GSH synthesis, our 15N-leucine tracing data support a starvation-specific impairment in BCAA-dependent input into GSH synthesis in *AkhR* mutants.

6) Bcat mutants are shown to have lower NEAA levels during starvation, which the authors interpret as evidence for production of these from BCAAs. However, comparing the Bcat mutant profile during starvation (Fig 6a) with autophagy mutants (Fig 4g), suggests that EAAs are generally dependent on FB autophagy but NEAAs are less dependent on it and some even increase, including Glu - the primary recipient of the amino group of BCAAs as shown in Fig 6c. The conclusion that BCAAs are required to make NEAAs such as Glu is therefore problematic.

Response: We agree that interpretation of steady-state amino-acid levels during starvation is not straightforward, because amino-acid abundances reflect the net balance between production (e.g., autophagy/protein degradation) and consumption (e.g., protein synthesis and downstream metabolism). We also agree that NEAAs, including glutamate, are not generated exclusively from BCAA catabolism.

Accordingly, our intention is not to claim that BCAAs are strictly required to produce NEAAs such as glutamate. Thus, changes in glutamate abundance can reflect shifts in multiple inputs and uses of glutamate, not only BCAA-derived nitrogen transfer. Rather, our data support that Bcat-dependent BCAA catabolism contributes to NEAA homeostasis under starvation, and that this contribution is diminished in *Bcat* mutants. For example, glutamate can be supplied from multiple metabolic inputs, including TCA cycle–derived α-ketoglutarate (which can be generated from carbohydrate-derived carbon, e.g., glucose) and transamination reactions involving amino groups from sources other than BCAAs. Therefore, a direct one-to-one comparison of amino-acid profiles between *Bcat* mutants and fat body autophagy mutants is

challenging, because these perturbations affect amino-acid production/consumption through distinct mechanisms.

7) Existing literature not really cited by the authors shows that Akh signalling in neurons regulates behavioural responses to starvation such as foraging (Yu et al 2016, Jourjine et al 2016). Hyperactivity/foraging is very likely an important aspect of energy expenditure during starvation and would be expected to be absent in AkhR mutants. This means that the link between Akh signalling and mobilisation of stored nutrients could be very indirect and not necessarily consistent with the authors conclusions.

Response: We agree that Akh signaling regulates not only metabolic processes but also starvation-associated behaviors, including activity/foraging, via neuronal pathways. Therefore, metabolic phenotypes observed in *AkhR* mutants during starvation can, in principle, reflect a combination of tissue-autonomous metabolic changes and indirect effects mediated by altered behavior and energy expenditure. We have revised the manuscript to discuss this point explicitly and to cite the relevant literature.

Importantly, however, several key conclusions in our study are based on endocrine and tissue-specific readouts (e.g., total/circulating Akh levels and fat body-specific manipulations), which are not easily explained solely by changes in foraging behavior. We therefore frame behavioral contributions as an important consideration/limitation rather than as an alternative explanation that negates the fat body feedback mechanism described here.

8) The interpretations in this paper often rely heavily upon large effect sizes in Akhr mutants. How physiological is the Akh hypersecretion (Akhr mutant) effect being studied if, as the authors show, it does not depend upon the same Ampk mechanism as normal secretion? Furthermore, the statement (line 333-4) is not supported by the evidence in the paper :" these findings strongly support a positive regulatory role of BCAAs in Akh secretion". To make sense of the Cg>hBCAT2 overexpression results, we need comparisons with flies that are wildtype at the AkhR locus. Currently, it is not possible to see how hBCAT2 functions in the physiological context where Akh negative feedback has not been removed.

Response: Thank you for these insightful comments. Our rationale for using *AkhR* mutants is that they genetically remove the negative-feedback arm of Akh signaling in the fat body, thereby unmasking the underlying feedback architecture that buffers endocrine output in wild-type animals. In wild-type flies, this feedback is expected to dampen changes in circulating Akh,

making effect sizes smaller and more difficult to resolve. Thus, the hypersecretion phenotype in *AkhR* mutants should be viewed as a consequence of disrupting feedback control, rather than as a claim that wild-type physiology operates at the same magnitude. This logic is analogous to well-established endocrine negative-feedback systems in mammals, where disruption of hormone action at the target tissue can lead to compensatory elevations in upstream hormone output (e.g., loss of end-organ feedback in the hypothalamic–pituitary axes).

To address the reviewer's point, we examined *Cg>hBcat2* flies in wild-type background to evaluate the effect of enhanced BCAA catabolism under intact negative feedback. hBcat2 overexpression significantly reduced BCAA levels, especially under starvation (**Fig. 5b**). Notably, lipolysis during starvation was significantly suppressed, suggesting reduced Akh signaling. However, hBcat2 overexpression in the fat body also markedly reduced total Akh levels under fed conditions, presumably due to chronic lowering of BCAA availability. Because this manipulation alters basal Akh production/peptide stores, it becomes difficult to interpret the starvation phenotype solely in terms of changes in secretion.

As an alternative approach, we analyzed circulating Akh levels in starved flies in which fat body autophagy was inhibited by *Atg1* knockdown. Unlike hBcat2 overexpression, total Akh levels were not reduced under fed conditions in this manipulation (**Fig. 5d**). Consistent with our original observation that inhibition of fat body autophagy suppresses the starvation-induced increase in BCAA levels and lipolysis, we found that circulating Akh levels were significantly reduced during starvation (**Fig. 5e**). These results support the conclusion that fat body-derived nutrients promote starvation-induced Akh secretion and downstream lipolysis. Because multiple amino acids are altered in this condition (**Fig. 5c**), the specific contribution of BCAAs versus other amino acids remains to be determined.

9) In Fig 6d there is substantial labelling of many NEAAs from 15N-leucine. This high NEAA labelling may, unfortunately, indicate a profound metabolic rewiring caused by adding a bolus of leucine to the starvation medium. The interpretation therefore has to be more cautious, along the lines of "Leucine can act as a nitrogen donor for NEAA synthesis during starvation".

Response: We agree with this comment. We acknowledge that providing a bolus of 15N-leucine in an agar-only starvation diet could, in principle, induce metabolic adaptation and affect labeling patterns. To minimize this potential artifact, we designed the tracing protocol in two ways: (1) flies were first starved for 12 h to establish a normal starvation response, and then fed 15N-leucine for 12 h; and (2) we used a relatively low leucine concentration (1 mM),

approximately one-tenth of the leucine concentration in the holidic diet (Piper et al., 2014). Nevertheless, we cannot exclude the possibility that leucine feeding influences metabolic state and labeling outcomes. We therefore revised the text to interpret these data more cautiously.

MINOR:

The statement that "AkhR is mainly expressed in the fat body" is an overstatement as FlyAtlas 2 shows AkhR expressed highly in several other tissues including heart, eye and carcass.

Response: Accordingly, we have revised the manuscript to state that AkhR is enriched in the fat body based on single-cell RNA-seq datasets (Li et al., 2022), rather than attributing this conclusion to FlyAtlas2 (Leader et al., 2018).

The model in Fig7h shows that Akhr activation leads to upregulation of Bcat and the evidence indicates that this is at the mRNA level. The authors have not discussed what candidate mechanisms might be responsible for this link.

Response: Thank you for pointing out this important issue. At present, we have not identified the downstream transcriptional regulators that link AkhR signaling to *Bcat* induction in the fat body. Interestingly, in mammals, glucagon signaling through the hepatic glucagon receptor is known to upregulate the expression of multiple amino acid transporters and enzymes involved in amino-acid catabolism (Solloway et al., 2015; Dean et al., 2017; Kim et al., 2017), although the full set of transcription factors mediating these effects remains incompletely defined. Consistent with the idea that starvation broadly remodels amino-acid metabolism at the transcriptional level, our preliminary RNA-seq analysis of adult abdominal carcasses indicates that starvation induces widespread changes in the expression of amino-acid metabolic enzymes. We therefore speculate that *Bcat* is one of the metabolic genes transcriptionally regulated downstream of Akh signaling during starvation. We have added a brief discussion of this point in the revised manuscript. Identifying the signaling and transcriptional components connecting AkhR activation to *Bcat* expression will be an important direction for future work.

Line 70-71. The following statement is confusing. "Unlike stored carbohydrates and lipids, amino acids serve dual roles as both structural components and metabolic fuels". Surely lipids contribute to cell membranes - which would be a structural role.

Response: We agree that lipids also have structural roles. Accordingly, we have revised the sentence.

The mating status of the adult flies is not mentioned. important, especially given the sex-specific effects being reported.

Response: Thank you for raising this point. For experiments using females, we used adult females housed with males (mated conditions). For experiments using males, we used adult males housed with females. We have now explicitly stated these conditions in the Methods.

Reviewer #4 (Remarks to the Author):
I co-reviewed this manuscript with one of the reviewers who provided the listed reports. This is part of the Nature Communications initiative to facilitate training in peer review and to provide appropriate recognition for Early Career Researchers who co-review manuscripts.

Response: We thank the reviewer for their contribution and for participating in the Nature Communications co-review initiative.

References

Ahmad, M., Wu, S., Luo, S., Shi, W., Guo, X., Cao, Y., Perrimon, N. and He, L. (2025). Dietary amino acids promote glucagon-like hormone release to generate global calcium waves in adipose tissues in Drosophila. *Nat Commun* 16, 247.

Braco, J. T., Gillespie, E. L., Alberto, G. E., Brenman, J. E. and Johnson, E. C. (2012). Energy-dependent modulation of glucagon-like signaling in Drosophila via the AMP-activated protein kinase. *Genetics* 192, 457-466.

Casas-Vila, N., Bluhm, A., Sayols, S., Dinges, N., Dejung, M., Altenhein, T., Kappei, D., Altenhein, B., Roignant, J. Y. and Butter, F. (2017). The developmental proteome of Drosophila melanogaster. *Genome Res.* 27, 1273-1285.

Dean, E. D., Li, M., Prasad, N., Wisniewski, S. N., Von Deylen, A., Spaeth, J., Maddison, L., Botros, A., Sedgeman, L. R., Bozadjieva, N., et al. (2017). Interrupted Glucagon Signaling Reveals Hepatic alpha Cell Axis and Role for L-Glutamine in alpha Cell Proliferation. *Cell Metab.* 25, 1362-1373 e1365.

Deleyto-Seldas, N. and Efeyan, A. (2021). The mTOR-Autophagy Axis and the Control of Metabolism. *Front Cell Dev Biol* 9, 655731.

Galikova, M., Diesner, M., Klepsatel, P., Hehlert, P., Xu, Y., Bickmeyer, I., Predel, R. and Kuhnlein, R. P. (2015). Energy Homeostasis Control in Drosophila Adipokinetic Hormone Mutants. *Genetics* 201, 665-683.

Gronke, S., Muller, G., Hirsch, J., Fellert, S., Andreou, A., Haase, T., Jackle, H. and Kuhnlein, R. P. (2007). Dual lipolytic control of body fat storage and mobilization in Drosophila. *PLoS Biol.* 5, e137.

Guntur, A. R., Gu, P., Takle, K., Chen, J., Xiang, Y. and Yang, C. H. (2015). Drosophila TRPA1 isoforms detect UV light via photochemical production of H2O2. *Proc. Natl. Acad. Sci. U. S. A.* 112, E5753-5761.

Kim, J., Okamoto, H., Huang, Z., Anguiano, G., Chen, S., Liu, Q., Cavino, K., Xin, Y., Na, E., Hamid, R., et al. (2017). Amino Acid Transporter Slc38a5 Controls Glucagon Receptor Inhibition-Induced Pancreatic alpha Cell Hyperplasia in Mice. *Cell Metab.* 25, 1348-1361 e1348.

Kim, S. K. and Rulifson, E. J. (2004). Conserved mechanisms of glucose sensing and regulation by Drosophila corpora cardiaca cells. *Nature* 431, 316-320.

Kodrik, D., Bednarova, A., Zemanova, M. and Krishnan, N. (2015). Hormonal Regulation of Response to Oxidative Stress in Insects-An Update. *Int. J. Mol. Sci.* 16, 25788-25816.

Leader, D. P., Krause, S. A., Pandit, A., Davies, S. A. and Dow, J. A T. (2018). FlyAtlas 2: a new version of the Drosophila melanogaster expression atlas with RNA-Seq, miRNA-Seq and sex-specific data. *Nucleic Acids Res.* 46, D809-D815.

Li, H., Janssens, J., De Waegeneer, M., Kolluru, S. S., Davie, K., Gardeux, V., Saelens, W., David, F. P. A., Brbic, M., Spanier, K., et al. (2022). Fly Cell Atlas: A single-nucleus transcriptomic atlas of the adult fruit fly. *Science* 375, eabk2432.

Marygold, S. J. (2024). The alpha-ketoacid dehydrogenase complexes of Drosophila melanogaster. *MicroPubl Biol* 2024, 10.17912.

Pauls, D., Selcho, M., Raderscheidt, J., Amatobi, K. M., Fekete, A., Krischke, M., Hermann-Luibl, C., Ozbek-Unal, A. G., Ehmann, N., Itskov, P. M., et al. (2021). Endocrine signals fine-tune daily activity patterns in Drosophila. *Curr. Biol.* 31, 4076-4087 e4075.

Piper, M. D., Blanc, E., Leitao-Goncalves, R., Yang, M., He, X., Linford, N. J., Hoddinott, M. P., Hopfen, C., Soultoukis, G. A., Niemeyer, C., et al. (2014). A holidic medium for Drosophila melanogaster. *Nat. Methods* 11, 100-105.

Roach, P. J., Depaoli-Roach, A. A., Hurley, T. D. and Tagliabracci, V. S. (2012). Glycogen and its metabolism: some new developments and old themes. *Biochem. J.* 441, 763-787.

Solloway, M. J., Madjidi, A., Gu, C., Eastham-Anderson, J., Clarke, H. J., Kljavin, N., Zavala-Solorio, J., Kates, L., Friedman, B., Brauer, M., et al. (2015). Glucagon Couples Hepatic Amino Acid Catabolism to mTOR-Dependent Regulation of alpha-Cell Mass. *Cell Rep.* 12, 495-510.

We are grateful to all reviewers for taking the time to review our manuscript and provide constructive comments. We have taken all comments into consideration and have made appropriate changes to the manuscript. Our point-by-point response appears below (in **blue text**). We hope that the revised manuscript is now suitable for publication in *Nature Communications*.

Reviewer #1 (Remarks to the Author):

The authors have robustly responded to the reviews, especially by performing new stable isotope labeling experiments to show that Bcat expression controls BCAA flux and showing mTOR activation. I think they could make a stronger statement regarding the suppression of autophagy by mTOR as this is fairly standard biology. In any case the authors should be applauded for their comprehensive response to the reviewer critiques.

Response: We thank the reviewer for the positive assessment.

Reviewer #2 (Remarks to the Author):

Overall, the authors appear to have carefully addressed the major concerns raised in the previous round of review and have performed the requested experiments in a diligent manner. This manuscript is of high significance, particularly due to the novelty of the quantitative Akh measurement approach and the depth of the metabolic analyses enabled by this method. The study provides compelling evidence that the Akh–AkhR signaling axis, a glucagon-like endocrine pathway in Drosophila, exerts broad metabolic effects, including regulation of BCAA metabolism, autophagy, and redox homeostasis, and mediates inter-organ communication between the corpora cardiaca (CC) and the fat body.

While the overall quality and importance of the work are clear, the previous version of the manuscript suffered from substantial complexity, with multiple lines of evidence presented in parallel and, in some cases, apparently conflicting observations listed without sufficient contextual explanation. In the revised manuscript, many of these issues have been substantially improved. The data are better organized, and several previously fragmented or confusing sections have been clarified, resulting in a more coherent narrative.

That said, I believe the manuscript would benefit from a few additional, relatively minor revisions that would further improve clarity and accessibility for readers. I therefore recommend

minor revision, with the following specific points to be addressed:

1. Rationale for extensive use of AkhR mutant lines
In the rebuttal, the authors provide a clear and convincing explanation for their repeated use of AkhR mutant lines, particularly in the context of revealing the physiological negative feedback structure of the Akh–AkhR axis. However, this rationale is not explicitly stated in the manuscript itself. I recommend that the authors incorporate a brief explanation into the main text, clarifying the primary purpose of using AkhR mutants and how this approach helps expose the underlying feedback regulation.

Response: Thank you for the constructive comments. We accordingly added the following sentences to the Discussion section (lines 547-551).
"Our rationale for using *AkhR* mutants throughout the study was to unmask the physiological negative-feedback architecture that operates in wild-type flies. In wild-type conditions, the feedback loop between the fat body and CC cells buffers both Akh secretion and downstream metabolite levels, making it difficult to observe large effect sizes."

2. Interpretation of amino acid levels under starvation
In the rebuttal, the authors appropriately note that amino acid levels measured under starvation conditions reflect a net effect of multiple processes, including autophagy/protein degradation, protein synthesis, and downstream metabolic consumption. This important clarification should be explicitly incorporated into the Results section. Without this explanation, the initial presentation of the amino acid data risks confusing readers, as net pool size changes may be mistakenly interpreted as direct readouts of Akh–AkhR signaling or CC–fat body inter-organ communication. A clear statement at the point where these data are first introduced would greatly aid interpretation.

Response: Accordingly, we added the following clarification regarding amino acid levels to the Results section (lines 278-281).
"Because these measurements report whole-body amino-acid abundances (i.e., pool sizes), they reflect the net balance between production (e.g., autophagy/protein degradation) and consumption (e.g., protein synthesis and downstream metabolism). Therefore, these data do not directly report metabolic flux."

3. Direct testing of BCAA effects on CC cell activity
The authors attempted to directly test whether BCAAs can acutely regulate CC cell activity, as

requested in the first revision. Although these experiments yielded negative results, I consider this effort to be meaningful and valuable. I encourage the authors to present these data more fully—properly quantified and statistically analyzed—in a supplementary figure. Moreover, the interpretation of these negative results, currently discussed in the rebuttal, should be further elaborated and incorporated into the Discussion. Demonstrating that this possibility was directly tested, even with negative outcomes, strengthens the overall rigor of the study.

Response: Thank you for the suggestion. We accordingly added the corresponding supplementary Figure (**Fig. S11)** to the Results section (lines 524-532).
"Lastly, we performed ex vivo calcium imaging in adult CC cells to test whether BCAAs directly evoke an acute activation signal. While high K+ robustly increased GCaMP signals, we detected no significant calcium response to elevated BCAAs under our adult ex vivo CC imaging conditions (**Fig. S11a-d**). We also detected no significant responses to glycine or threonine, which have been reported to stimulate larval CC cells (Ahmad et al., 2025). These results indicate that, under our assay conditions, adult CC cells do not show an acute calcium response to these amino acids. This difference could reflect stage-specific properties of CC cells and/or limitations of the current ex vivo conditions for capturing amino-acid-evoked responses in adults."

In addition, we added the following discussion (lines 603-611).
"Our ex vivo imaging results do not support acute BCAA induced calcium activation in adult CC cells under the conditions tested. We note that ex vivo calcium imaging has been extensively performed in larval CC cells (Ahmad et al., 2025; Braco et al., 2012; Guntur et al., 2015; Kim and Rulifson, 2004; Pauls et al., 2021), whereas there are no established protocols or published studies demonstrating robust stimulus-evoked calcium responses in adult CC cells. Because CC cell responses to starvation are likely stage-dependent, the absence of detectable amino acid induced calcium responses in our adult ex vivo assay could reflect stage-specific properties of CC cells and/or limitations of the current assay conditions. Importantly, the absence of an acute calcium signal does not by itself rule out direct regulation via other signaling modalities."

Reviewer #3 (Remarks to the Author):

The revised manuscript now satisfactorily addresses several of our major points (2-5 and 7-9). However, three major points (1, 3, 6) have not been adequately addressed and/or continue to cast serious doubt upon the authors interpretations and large parts of their mechanistic model in Fig. 7h (now 8h):

Major point 1) Along with another reviewer, we raised the important point that there was no solid evidence to support the CC model (Fig7h) in which BCAAs act on the CC to stimulate Akh secretion. The authors have now done well to conduct new ex vivo calcium imaging and Tor signaling experiments to address this issue. Unfortunately, however, these new experiments failed to provide any evidence that BCAAs act on the CC to stimulate Akh signalling. So the most parsimonious explanation, in line with a previous study (PMID: 39747032), is that the CC does not respond to BCAAs. This part of the Fig 7h, now 8h, model is therefore incorrect.

Response: We accordingly revised the proposed model (**Fig. 8h**) by indicating a question mark on the dashed arrow between BCAAs and CC cells.

We also revised the corresponding figure legend (**Fig. 8h**) to avoid misinterpretation:
"Increased BCAAs during starvation are proposed to promote Akh secretion and act as metabolic cues for catabolism regulation, although the site and mechanism remain unresolved." (lines 1370-1372)

In addition, we specified in the Discussion that CC cells show no acute response to BCAAs under our ex vivo conditions:
"Our ex vivo imaging results do not support acute BCAA induced calcium activation in adult CC cells under the conditions tested." (lines 603-604)
"In addition, our data are consistent with an indirect route in which BCAAs influence Akh secretion via intermediate metabolic and/or neural pathways." (lines 617-619)

We emphasize that the central conclusion of the manuscript does not depend on a direct BCAA-to-CC interaction. Rather, our data establish a feedback architecture linking fat body amino acid metabolism and Akh signaling, while leaving open the precise mechanism by which nutrient status modulates CC output.

Major point 3) In light of our comments here, the authors seem to have reinterpreted their data such that Akh signaling is no longer required for mobilization of TAG (and glycogen) stores. This part of the Fig 7h, now 8h, model is therefore questionable. The authors replacement of the text with “regulates/modulates” rather than “is required for” is still an unsubstantiated claim.

Response: This reviewer raised the concern regarding the function of Akh signaling in lipolysis during starvation. Akh signaling has been widely implicated in lipid mobilization during

starvation across insects, including *Drosophila* (e.g., Chatterjee and Perrimon, 2021; Heier and Kuhnlein, 2018). Importantly, previous work in *Drosophila* has demonstrated that *AkhR* mutant fat body cells lack an early lipolysis response but exhibit strong TAG cleavage activity after extended food deprivation due to the presence of starvation-induced lipase bmm (Gronke et al., 2007). Thus, redundancy and compensation with the bmm pathway can mask large effect sizes in *AkhR* mutant backgrounds. Consistent with this framework, we note that combined disruption of Akh signaling and the bmm pathway (i.e., *AkhR*; *bmm* double mutants) results in impaired lipid mobilization and reduced starvation resistance, supporting the notion that these two pathways act in parallel to promote TAG mobilization during starvation (Gronke et al., 2007). Accordingly, our model (**Fig. 8h**) depicts lipolysis as being driven by both an Akh-dependent pathway and the bmm pathway.

We have already discussed this issue in the Discussion:
"Fat body lipolysis is regulated by two distinct mechanisms: Akh-dependent signaling and the starvation-induced lipase bmm pathway (Gronke et al., 2005; Wat et al., 2020). Although these systems function redundantly, they also communicate in a compensatory manner to ensure lipid mobilization (Gronke et al., 2007)." (lines 644-647)

To further satisfy the reviewer's concern, we added the following sentence to the figure legend (**Fig. 8h**) to avoid misinterpretation:
"Of note, Akh signaling and bmm act in parallel and can compensate for each other to support lipid mobilization during starvation."(lines 1372-1373)

Major point 6) This criticism has not been adequately addressed. In both the original and revised manuscript there continues to be no solid evidence for yet another key part of the Fig 7h model, namely that NEAAs (and GSH) are synthesized from BCAAs via autophagy and in response to Akh signaling. Fig 5c shows that autophagy mutants lower BCAA levels but increase Glu levels. Thus, it cannot be ruled out that other nitrogen donors exist and can be utilised to make NEAAs and possibly GSH, which the authors don’t test.

Response: We agree with the reviewer that our data cannot rule out the possibility that other nitrogen donors exist and can be used to support the synthesis of NEAAs and GSH. Accordingly, we clarified this point in the Discussion section (lines 656-660):
"Our findings provide an additional mechanism by which Akh promotes redox homeostasis through induction of BCAA catabolism, thereby supporting the synthesis of NEAAs and GSH. These results do not exclude additional nitrogen donors that could also support NEAA synthesis

and redox buffering."

We also revised the figure legend (**Fig. 8h**) to avoid misinterpretation:
"BCAA catabolism contributes to both energy and redox homeostasis by maintaining TCA cycle flux and supporting GSH synthesis in part through NEAA production, though additional nitrogen sources can also contribute." (lines 1362-1370)

In conclusion, the authors responses to major points 1,3 and 6 do not provide sufficient missing evidence to plug numerous mechanistic gaps in the Fig. 8h model. On balance, we therefore regretfully conclude that what remains intact in the model is, at best, borderline in terms of any substantial mechanistic advance.

Response: We appreciate the reviewer's overall assessment and understand the concern that Fig. 8h could be overinterpreted as a fully resolved mechanism. We therefore revised Fig. 8h and its legend to clearly distinguish supported relationships from unresolved links, and we incorporated additional discussion to delineate the scope and limitations of our conclusions. We believe that the revised manuscript presents a rigorously supported framework for Akh-dependent inter-organ feedback during starvation while explicitly noting the remaining open mechanistic questions.

Importantly, we would like to clarify that the principal advance of this study does not lie in establishing every individual molecular step, but rather in defining a previously uncharacterized endocrine feedback architecture linking fat body amino acid metabolism and Akh signaling during starvation. Our data provide direct quantitative measurements of circulating Akh, stable isotope evidence for BCAA contribution to redox metabolism, genetic epistasis analyses, and tissue-specific perturbations that collectively support this feedback framework.

While we fully acknowledge that the precise mechanisms of how nutrient-derived cues influence CC output remain to be elucidated, we believe that identifying and experimentally validating this inter-organ feedback structure represents a substantial conceptual advance.

Reviewer #4 (Remarks to the Author):
I co-reviewed this manuscript with one of the reviewers who provided the listed reports. This is part of the Nature Communications initiative to facilitate training in peer review and to provide appropriate recognition for Early Career Researchers who co-review manuscripts.

Response: We thank the reviewer for their contribution and for participating in the Nature Communications co-review initiative.

References

Ahmad, M., Wu, S., Luo, S., Shi, W., Guo, X., Cao, Y., Perrimon, N. and He, L. (2025). Dietary amino acids promote glucagon-like hormone release to generate global calcium waves in adipose tissues in Drosophila. *Nat Commun* 16, 247.

Braco, J. T., Gillespie, E. L., Alberto, G. E., Brenman, J. E. and Johnson, E. C. (2012). Energy-dependent modulation of glucagon-like signaling in Drosophila via the AMP-activated protein kinase. *Genetics* 192, 457-466.

Chatterjee, N. and Perrimon, N. (2021). What fuels the fly: Energy metabolism in Drosophila and its application to the study of obesity and diabetes. *Sci Adv* 7, eabg4336.

Gronke, S., Mildner, A., Fellert, S., Tennagels, N., Petry, S., Muller, G., Jackle, H. and Kuhnlein, R. P. (2005). Brummer lipase is an evolutionary conserved fat storage regulator in Drosophila. *Cell Metab.* 1, 323-330.

Gronke, S., Muller, G., Hirsch, J., Fellert, S., Andreou, A., Haase, T., Jackle, H. and Kuhnlein, R. P. (2007). Dual lipolytic control of body fat storage and mobilization in Drosophila. *PLoS Biol.* 5, e137.

Guntur, A. R., Gu, P., Takle, K., Chen, J., Xiang, Y. and Yang, C. H. (2015). Drosophila TRPA1 isoforms detect UV light via photochemical production of H2O2. *Proc. Natl. Acad. Sci. U. S. A.* 112, E5753-5761.

Heier, C. and Kuhnlein, R. P. (2018). Triacylglycerol Metabolism in Drosophila melanogaster. *Genetics* 210, 1163-1184.

Kim, S. K. and Rulifson, E. J. (2004). Conserved mechanisms of glucose sensing and regulation by Drosophila corpora cardiaca cells. *Nature* 431, 316-320.

Pauls, D., Selcho, M., Raderscheidt, J., Amatobi, K. M., Fekete, A., Krischke, M., Hermann-Luibl, C., Ozbek-Unal, A. G., Ehmann, N., Itskov, P. M., et al. (2021). Endocrine signals fine-tune daily activity patterns in Drosophila. *Curr. Biol.* 31, 4076-4087 e4075.

Wat, L. W., Chao, C., Bartlett, R., Buchanan, J. L., Millington, J. W., Chih, H. J., Chowdhury, Z. S., Biswas, P., Huang, V., Shin, L. J., et al. (2020). A role for triglyceride lipase brummer in the regulation of sex differences in Drosophila fat storage and breakdown. *PLoS Biol.* 18, e3000595.
